# Supplementary material for: Thermogenic Differentiation of Human Adipocyte Precursors in Culture: A Systematic Review
Source: Cells. 2025 Dec 2;14(23):1907. doi: 10.3390/cells14231907 (PMC12691314; doi:10.3390/cells14231907)
Supplement: Supplementary file 1 [file cells-14-01907-s001.zip › cells-3771228-supplementary.pdf]

Supplementary Table S1. Preferred Reporting Items for Systematic Reviews and Meta-Analyses checklist.

Section and Topic	Item #	Checklist item	Location where item is reported
**TITLE**			
Title	1	Identify the report as a systematic review.	1
**ABSTRACT**			
Abstract	2	See the PRISMA 2020 for Abstracts checklist.	1
**INTRODUCTION**			
Rationale	3	Describe the rationale for the review in the context of existing knowledge.	1,2
Objectives	4	Provide an explicit statement of the objective(s) or question(s) the review addresses.	2
**METHODS**			
Eligibility criteria	5	Specify the inclusion and exclusion criteria for the review and how studies were grouped for the syntheses.	2
Information sources	6	Specify all databases, registers, websites, organisations, reference lists and other sources searched or consulted to identify studies. Specify the date when each source was last searched or consulted.	2
Search strategy	7	Present the full search strategies for all databases, registers and websites, including any filters and limits used.	Supplementary Table S2
Selection process	8	Specify the methods used to decide whether a study met the inclusion criteria of the review, including how many reviewers screened each record and each report retrieved, whether they worked independently, and if applicable, details of automation tools used in the process.	2,3
Data collection process	9	Specify the methods used to collect data from reports, including how many reviewers collected data from each report, whether they worked independently, any processes for obtaining or confirming data from study investigators, and if applicable, details of automation tools used in the process.	2,3
Data items	10a	List and define all outcomes for which data were sought. Specify whether all results that were compatible with each outcome domain in each study were sought (e.g. for all measures, time points, analyses), and if not, the methods used to decide which results to collect.	3
	10b	List and define all other variables for which data were sought (e.g. participant and intervention characteristics, funding sources). Describe any assumptions made about any missing or unclear information.	3
Study risk of bias assessment	11	Specify the methods used to assess risk of bias in the included studies, including details of the tool(s) used, how many reviewers assessed each study and whether they worked independently, and if applicable, details of automation tools used in the process.	3
Effect measures	12	Specify for each outcome the effect measure(s) (e.g. risk ratio, mean difference) used in the synthesis or presentation of results.	3
Synthesis methods	13a	Describe the processes used to decide which studies were eligible for each synthesis (e.g. tabulating the study intervention characteristics and comparing against the planned groups for each synthesis (item #5)).	3
	13b	Describe any methods required to prepare the data for presentation or synthesis, such as handling of missing summary statistics, or data conversions.	-
	13c	Describe any methods used to tabulate or visually display results of individual studies and syntheses.	3
	13d	Describe any methods used to synthesize results and provide a rationale for the choice(s). If meta-analysis was performed, describe the model(s), method(s) to identify the presence and extent of statistical heterogeneity, and software package(s) used.	-
	13e	Describe any methods used to explore possible causes of heterogeneity among study results (e.g. subgroup analysis, meta-regression).	-
	13f	Describe any sensitivity analyses conducted to assess robustness of the synthesized results.	-
Reporting bias assessment	14	Describe any methods used to assess risk of bias due to missing results in a synthesis (arising from reporting biases).	NA
Certainty	15	Describe any methods used to assess certainty (or confidence) in the body of evidence for an outcome.	NA

Section and Topic	Item #	Checklist item	Location where item is reported
assessment			
**RESULTS**			
Study selection	16a	Describe the results of the search and selection process, from the number of records identified in the search to the number of studies included in the review, ideally using a flow diagram.	3
	16b	Cite studies that might appear to meet the inclusion criteria, but which were excluded, and explain why they were excluded.	Supplementary Table S5
Study characteristics	17	Cite each included study and present its characteristics.	Supplementary Table S3
Risk of bias in studies	18	Present assessments of risk of bias for each included study.	Supplementary Table S6
Results of individual studies	19	For all outcomes, present, for each study: (a) summary statistics for each group (where appropriate) and (b) an effect estimate and its precision (e.g. confidence/credible interval), ideally using structured tables or plots.	NA
Results of syntheses	20a	For each synthesis, briefly summarise the characteristics and risk of bias among contributing studies.	NA
	20b	Present results of all statistical syntheses conducted. If meta-analysis was done, present for each the summary estimate and its precision (e.g. confidence/credible interval) and measures of statistical heterogeneity. If comparing groups, describe the direction of the effect.	NA
	20c	Present results of all investigations of possible causes of heterogeneity among study results.	NA
	20d	Present results of all sensitivity analyses conducted to assess the robustness of the synthesized results.	NA
Reporting biases	21	Present assessments of risk of bias due to missing results (arising from reporting biases) for each synthesis assessed.	NA
Certainty of evidence	22	Present assessments of certainty (or confidence) in the body of evidence for each outcome assessed.	NA
**DISCUSSION**			
Discussion	23a	Provide a general interpretation of the results in the context of other evidence.	5
	23b	Discuss any limitations of the evidence included in the review.	8
	23c	Discuss any limitations of the review processes used.	8
	23d	Discuss implications of the results for practice, policy, and future research.	8
**OTHER INFORMATION**			
Registration and protocol	24a	Provide registration information for the review, including register name and registration number, or state that the review was not registered.	2
	24b	Indicate where the review protocol can be accessed, or state that a protocol was not prepared.	-
	24c	Describe and explain any amendments to information provided at registration or in the protocol.	NA
Support	25	Describe sources of financial or non-financial support for the review, and the role of the funders or sponsors in the review.	14
Competing interests	26	Declare any competing interests of review authors.	14
Availability of data, code and other materials	27	Report which of the following are publicly available and where they can be found: template data collection forms; data extracted from included studies; data used for all analyses; analytic code; any other materials used in the review.	-

Supplementary Table S2. Search strategies with appropriated keywords and MeSH terms.

Database (search date)	Search
PubMed (October 30, 2023; updated on March 26, 2025)<br>N = 3,050	#1: “Adipocyte, Beige” OR “Beige Adipocyte” OR “Beige Adipocytes” OR “Beige Brite Adipocytes” OR “Adipocyte, Beige Brite” OR “Adipocytes, Beige Brite” OR “Beige Brite Adipocyte” OR “Brite Adipocyte, Beige” OR “Brite Adipocytes, Beige” OR “Brite Fat Cells” OR “Brite Fat Cell” OR “Cell, Brite Fat” OR “Cells, Brite Fat” OR “Fat Cell, Brite” OR “Fat Cells, Brite” OR “Beige Brite Cells” OR “Beige Brite Cell” OR “Brite Cell, Beige” OR “Brite Cells, Beige” OR “Cell, Beige Brite” OR “Cells, Beige Brite” OR “Adipocytes, Brite” OR “Adipocyte, Brite” OR “Brite Adipocyte” OR “Brite Adipocytes” OR “Beige Fat Cells” OR “Beige Fat Cell” OR “Cell, Beige Fat” OR “Cells, Beige Fat” OR “Fat Cell, Beige” OR “Fat Cells, Beige”<br>#2: “Adipocyte, Brown” OR “Brown Adipocyte” OR “Brown Fat Cells” OR “Brown Fat Cell” OR “Cell, Brown Fat” OR “Cells, Brown Fat” OR “Fat Cell, Brown” OR “Fat Cells, Brown” OR “Brown Adipocytes”<br>#3: human<br>#4: ((#1) OR (#2)) AND (#3)
Scopus (October 30, 2023; updated on March 26, 2025)<br>N = 2,306	TITLE-ABS-KEY (“Adipocyte, Beige” OR “Beige Adipocyte” OR “Beige Adipocytes” OR “Beige Brite Adipocytes” OR “Adipocyte, Beige Brite” OR “Adipocytes, Beige Brite” OR “Beige Brite Adipocyte” OR “Brite Adipocyte, Beige” OR “Brite Adipocytes, Beige” OR “Brite Fat Cells” OR “Brite Fat Cell” OR “Cell, Brite Fat” OR “Cells, Brite Fat” OR “Fat Cell, Brite” OR “Fat Cells, Brite” OR “Beige Brite Cells” OR “Beige Brite Cell” OR “Brite Cell, Beige” OR “Brite Cells, Beige” OR “Cell, Beige Brite” OR “Cells, Beige Brite” OR “Adipocytes, Brite” OR “Adipocyte, Brite” OR “Brite Adipocyte” OR “Brite Adipocytes” OR “Beige Fat Cells” OR “Beige Fat Cell” OR “Cell, Beige Fat” OR “Cells, Beige Fat” OR “Fat Cell, Beige” OR “Fat Cells, Beige” OR “Adipocyte, Brown” OR “Brown Adipocyte” OR “Brown Fat Cells” OR “Brown Fat Cell” OR “Cell, Brown Fat” OR “Cells, Brown Fat” OR “Fat Cell, Brown” OR “Fat Cells, Brown” OR “Brown Adipocytes”) AND TITLE-ABS-KEY (human)
Web of Science (October 30, 2023; updated on March 26, 2025)<br>N = 1,070	TS=(“Adipocyte, Beige” OR “Beige Adipocyte” OR “Beige Adipocytes” OR “Beige Brite Adipocytes” OR “Adipocyte, Beige Brite” OR “Adipocytes, Beige Brite” OR “Beige Brite Adipocyte” OR “Brite Adipocyte, Beige” OR “Brite Adipocytes, Beige” OR “Brite Fat Cells” OR “Brite Fat Cell” OR “Cell, Brite Fat” OR “Cells, Brite Fat” OR “Fat Cell, Brite” OR “Fat Cells, Brite” OR “Beige Brite Cells” OR “Beige Brite Cell” OR “Brite Cell, Beige” OR “Brite Cells, Beige” OR “Cell, Beige Brite” OR “Cells, Beige Brite” OR “Adipocytes, Brite” OR “Adipocyte, Brite” OR “Brite Adipocyte” OR “Brite Adipocytes” OR “Beige Fat Cells” OR “Beige Fat Cell” OR “Cell, Beige Fat” OR “Cells, Beige Fat” OR “Fat Cell, Beige” OR “Fat Cells, Beige” OR “Adipocyte, Brown” OR “Brown Adipocyte” OR “Brown Fat Cells”

	OR “Brown Fat Cell” OR “Cell, Brown Fat” OR “Cells, Brown Fat” OR “Fat Cell, Brown” OR “Fat Cells, Brown” OR “Brown Adipocytes”) AND TS=(human)
Google Scholar (October 30, 2023; updated on March 26, 2025)<br><br>Results: 100	(“brown adipocyte” OR “beige adipocyte”) AND (human)

Supplementary Table S3. Characteristics of the included studies (n = 117).

Study, year, country, study objective [reference in the manuscript]	Cell type, growth medium, confluency at induction	Differentiation protocol	Comparator	Outcomes	Results
Batrow et al., 2025<br>France [11]	ADSCs (pubic region, SC, child) and SVF from WAT (abdominal SC)<br><br>Growth medium<br>DMEM<br>10% FBS<br>15 mM HEPES<br>60 mg/mL penicillin<br>50 mg/mL streptomycin<br>2.5 ng/mL FGF2 (until confluency)<br><br>Induction 2 d after confluency	Beige adipocytes<br>DMEM-F12-Ham's<br>10 µg/mL transferrin<br>**D1-D2**<br>0.5 mM IBMX<br>1 µM dexa<br>10 nM insulin<br>0.2 nM T3<br>**D2-D4**<br>0.5 mM IBMX<br>1 µM dexa<br>10 nM insulin<br>0.2 nM T3<br>0.1 µM Rosi<br>**D4-D9**<br>Medium<br>**D14-D18**<br>0.1 µM Rosi	White adipocytes<br>DMEM-F12<br>10 µg/mL transferrin<br>**D1-D2**<br>0.5 mM IBMX<br>1 µM dexa<br>10 nM insulin<br>0.2 nM T3<br>**D2-D4**<br>0.5 mM IBMX<br>1 µM dexa<br>10 nM insulin<br>0.2 nM T3<br>100 nM Rosi<br>**D4-D18**<br>Medium	mRNA (UCP1, CPT1M, CIDEA)<br><br>Protein (UCP1)	Adipose-derived stem cells<br>Beige vs white<br>↑ UCP1, CPT1M, CIDEA mRNA<br>↑ UCP1 protein<br><br>SVF from adipose tissue<br>Beige vs white<br>↑ UCP1 mRNA
Desai et al., 2024<br>US [12]	Progenitor cells from WAT explants (abdominal SC)<br><br>Growth medium: NR<br><br>Induction at 100% confluency	DMEM<br>10% FBS<br>**D1-D3**<br>0.5 mM IBMX<br>0.25 µM dexa<br>5 µg/mL insulin<br>**D3-D6**<br>Replacement of half of the medium with DMEM + 10% FBS every other day<br>**D6-D10**<br>DMEM + 10% FBS<br>± 1 µM Rosi (3 to 48 h before harvesting)<br>± 1 µM FSK (3 to 48 h before harvesting)<br>± 1 µM Rosi and/or FSK 78 h before harvesting	Cells treated vs non-treated with Rosi<br><br>Cells treated vs non-treated with FSK<br><br>Cells treated vs non-treated with FSK and/or Rosi	mRNA (UCP1)<br><br>Protein (UCP1)<br><br>OCR (Seahorse®)	Cells treated vs non-treated with Rosi<br>↑ UCP1 mRNA<br><br>Cells treated vs non-treated with FSK<br>↑ UCP1 mRNA<br><br>Cells treated vs non-treated with forkskolin and/or Rosi<br>↑ UCP1 mRNA (Rosi, FSK, Rosi + FSK > vehicle)<br>↑ UCP1 protein (Rosi + FSK > Rosi and FSK > vehicle)<br>↑ ATP and proton leak-linked respiration (Rosi, FSK, Rosi+ FSK vs vehicle)<br>↑ maximal respiration (Rosi, FSK vs vehicle)
Díez-Sainz et al., 2024<br>Spain [13]	ADSCs<br><br>Growth medium<br>DMEM (low glucose)<br>L-GlutaMAX<br>10% FBS<br>15 mM HEPES<br>2.5 ng/mL FGF2 (until confluency)<br><br>Induction 2 d post-confluency	Brown/brite adipocytes<br>DMEM-F12-Ham's (low glucose)<br>L-GlutaMAX<br>L-Glutamine<br>1% penicillin/streptomycin<br>15 mM HEPES<br>10 µg/mL transferrin<br>**D1-D2**	White adipocytes<br>DMEM-F12-Ham's (low glucose)<br>L-GlutaMAX<br>L-Glutamine<br>1% penicillin/streptomycin<br>15 mM HEPES<br>10 µg/mL transferrin<br>**D1-D2**<br>0.5 mM IBMX	mRNA (UCP1, CPT1M, CIDEA, ADRB3)<br><br>Protein (UCP1)	Brown/brite vs white adipocytes<br>↑ UCP1, CPT1M, CIDEA mRNA<br>↔ ADRB3 mRNA<br>↑ UCP1 protein

		0.5 mM IBMX<br>1 μM dexa<br>10 nM insulin<br>**D2-D4**<br>0.5 mM IBMX<br>1 μM dexa<br>10 nM insulin<br>0.1 μM Rosi<br>**D4-D9**<br>10 nM insulin<br>0.2 nM T3<br>0.1 μM Rosi<br>**D9-D14**<br>10 nM insulin<br>0.2 nM T3<br>**D14-D18**<br>0.1 μM Rosi<br>10 nM insulin<br>0.2 nM T3	1 μM dexa<br>10 nM insulin<br>0.2 nM T3<br>**D2-D4**<br>0.5 mM IBMX<br>1 μM dexa<br>10 nM insulin<br>0.2 nM T3<br>100 nM Rosi<br>**D4-D18**<br>10 nM insulin<br>0.2 nM T3<br>100 nM Rosi		
Wu et al., 2024<br>US [14]	SVF from adipose tissue (source NR)<br><br>Growth mdium<br>DMEM (high glucose)<br>10% FBS<br><br>Induction at 100% confluency	Beige adipocytes<br>Growth medium +<br>**D1-D14**<br>33 μM biotin<br>17 μM pantothenate<br>0.5 mM IBMX<br>0.1 μM dexa<br>500 nM insulin<br>2 nM T3<br>30 μM indomethacin	-	mRNA (UCP1, UCP2, UCP3, PGC1α, PRDM16, TFAM)	Beige adipocytes<br>Detectable UCP1, UCP2, UCP3, PGC1α, PRDM16, TFAM mRNA
Colson et al., 2023<br>France, Germany [15]	MADS cells (SC WAT from children)<br><br>Growth medium<br>DMEM<br>10% FBS<br>15 mM HEPES<br>2.5 ng/mL FGF2<br>60 mg/mL penicillin<br>50 mg/mL streptomycin<br><br>Induction 2d after confluency	White to beige adipocyte conversion<br>DMEM-F12-Ham's<br>**D1-D4**<br>10 μg/mL transferrin<br>0.5 mM IBMX<br>1 μM dexa<br>10 nM insulin<br>0.2 nM T3<br>**D2-D9**<br>0.1 μM Rosi<br>**D14-D18**<br>0.1 μM Rosi (for white to beige conversion)	White adipocytes<br>DMEM/Ham's-F12<br>**D1-D4**<br>10 μg/mL transferrin<br>10 nM insulin<br>0.2 nM T3<br>1 uM dexa<br>500 μM IBMX<br>**D2-D9**<br>100 nM Rosi<br>**D14-D18**<br>No Rosi	mRNA (UCP1)<br><br>Protein (UCP1)	Beige vs white<br>↑ UCP1 mRNA<br>↑ UCP1 protein
Giroud et al., 2023<br>Germany [16]	SVF from WAT (SC)<br><br>Growth medium<br>EGM2<br>Induction 2 d after confluency<br><br>Adipose-derived stem cells (infant SC)<br>Growth medium<br>DMEM<br>10% FBS<br>10 mM HEPES	SVF from adipose tissue (SC)<br>DMEM-F12-Ham's<br>5 mM HEPES<br>2 mM L-glutamine<br>100 μg/mL normocin<br>10 μg/mL tranferrin<br>**D1-D7**<br>0.1 mM IBMX<br>1 μM dexa	SVF from adipose tissue (SC)<br>Cells treated vs non-treated with NE<br><br>Adipose-derived stem cells and SVF from adipose tissue (SC)<br>White adipocyte differentiation<br>DMEM-F12-Ham's<br>10 μg/mL transferrin	mRNA (UCP1, CIDEA, PGC1α)	SVF from adipose tissue (SC)<br>Cells treated vs non-treated with NE<br>↑ UCP1, CIDEA, PGC1α mRNA<br>Thermogenic vs white adipocyte differentiation<br>↑ UCP1 mRNA<br><br>Adipose-derived stem cells<br>↑ UCP1 mRNA

	2.5 ng/mL FGF2 (until confluency)<br>50 mg/mL penicillin<br>50 mg/mL streptomycin<br><br>Induction 2 d after confluency	860 nM insulin<br>0.2 nM T3<br>0.1 µM Rosi<br>**D7-D9**<br>0.1 mM IBMX<br>1 µM dexa<br>0.2 nM T3<br>0.1 µM Rosi<br>± 1 µM NE for 6 h before harvesting<br><br>Adipose-derived stem cells and SVF from adipose tissue (SC)<br>Thermogenic differentiation<br>DMEM-F12-Ham's<br>10 µg/mL apo-transferrin<br>**D1-D2**<br>1 mM IBMX<br>1 mM dexa<br>10 nM insulin<br>0.2 nM T3<br>100 nM Rosi<br>**D2-D9**<br>10 nM insulin<br>0.2 nM T3<br>1 µM Rosi<br>**D9-D14**<br>10 nM insulin<br>0.2 nM T3<br>± 1 µM FSK or NE for 6 h before harvesting	**D1-D2**<br>1 mM IBMX<br>1 mM dexa<br>10 nM insulin<br>0.2 nM T3<br>100 nM Rosi<br>**D2-D9**<br>10 nM insulin<br>0.2 nM T3<br>1 µM Rosi<br>**D9-D14**<br>10 nM insulin<br>0.2 nM T3<br>1 µM Rosi<br>± 1 µM FSK for 6 h before harvesting		↑ UCP1 mRNA in differentiated white adipocytes treated with NE vs non-treated<br>↔ UCP1 mRNA in differentiated thermogenic adipocytes treated with NE vs non-treated
Palani et al., 2023<br>Denmark [17]	SVF from adipose tissue (visceral, perirenal, abdominal SC, supraclavicular)<br><br>Growth medium<br>DMEM-F12<br>10% FBS<br>1% penicillin/streptomycin<br><br>Proliferation medium (pre-induction)<br>DMEM-F12<br>10% FBS<br>1% penicillin/streptomycin<br>1 nM FGF1<br><br>Induction 2d after confluency	Adipocyte differentiation<br>Growth medium without FBS and FGF1<br>+<br>**D1-D3**<br>0.54 mM IBMX<br>0.1 µM dexa<br>100 nM insulin<br>2 nM T3<br>0.2 µM Rosi<br>**D3-D6**<br>0.1 µM dexa<br>100 nM insulin<br>2 nM T3<br>0.2 µM Rosi<br>**D6-D12**<br>0.1µM dexa<br>100 nM insulin<br>2 nM T3	Cells differentiated from SVF from different adipose depots	Single-cell RNA seq (BATLAS)	Different mRNA expression (white and brown genes) between mature adipocytes differentiated from SVF of perirenal, supraclavicular, visceral and sc abdominal depots

Shon et al., 2023<br>South Korea [18]	ADSCs (Biosolution)<br><br>Growth medium<br>10% FBS<br>GlutaMAX<br>DMEM-F12<br>1% penicillin/streptomycin<br><br>Induction 2 d after confluency	Beige adipocytes<br>DMEM-F12<br>18.5 mM glucose<br>15 mM HEPES<br>25 mM $NaHCO_3$<br>100 units/mL penicillin<br>100 µg/mL streptomycin<br>33 µM biotin<br>17 µM pantothenate<br>**D1-D7**<br>0.5 mM IBMX<br>0.1 µM dexa<br>100 nM insulin<br>1 µM Rosi<br>2 nM T3<br>**D7-D15**<br>100 nM insulin<br>1 µM Rosi<br>2 nM T3<br><br>Or<br>White adipocytes<br>+ 1 µM NE for 6 h before harvesting	White adipocytes<br>DMEM-F12<br>18.5 mM glucose<br>15 mM HEPES<br>25 mM $NaHCO_3$<br>100 units/mL penicillin<br>100 µg/mL streptomycin<br>33 µM biotin<br>17 µM pantothenate<br>**D1-D3**<br>0.5 mM IBMX<br>100 nM dexa<br>100 nM insulin<br>1 µM Rosi<br>2 nM T3<br>**D3-D7**<br>0.5 mM IBMX<br>100 nM dexa<br>100 nM insulin<br>2 nM T3<br>**D7-D15**<br>100 nM insulin<br>1 µM Rosi<br>2 nM T3	mRNA (UCP1, CIDEA, CITED1, DIO2)	Beige or NE-stimulated white adipocytes vs white adipocytes<br>↑ UCP1, CIDEA, CITED1, DIO2 mRNA (both protocols induced thermogenic genes)
Suchacki et al., 2023<br>UK [19]	SVF from adipose tissue (DN, neck SC)<br><br>Growth medium<br>DMEM<br>10% FBS<br><br>Confluency at induction: NR	Growth medium<br>**D1-D7**<br>0.5 mM IBMX<br>0.5 µM dexa<br>20 nM insulin<br>1 nM T3<br>125 µM indomethacin<br>**D7-D14**<br>20 nM insulin<br>1 nM T3	Adipocytes differentiated from DN vs SC neck adipose tissue SVF	mRNA (UCP1)<br><br>Protein (UCP1)	Adipocytes differentiated from DN vs SC neck adipose tissue SVF<br>↑ UCP1 mRNA<br>↑ UCP1 protein
Vámos et al., 2023<br>Hungary [20]	SVF from WAT (abdominal SC)<br><br>Growth medium<br>DMEM-F12<br>10% FBS<br>17 µM pantothenate<br>33 µM biotin<br>100 U/mL penicillin/streptomycin<br><br>Induction at 100% confluency	Beige adipocytes<br>Growth medium without FBS +<br>10 µg/mL transferrin<br>**D1-D3**<br>0.5 mM IBMX<br>25 nM dexa<br>20 nM insulin<br>0.2 nM T3<br>2 µM Rosi<br>**D3-D25**<br>20 nM insulin<br>0.2 nM T3<br>20 nM human insulin<br>0.5 µM Rosi<br>**D14**<br>500 µM cAMP for 4 h	White adipocytes<br>Growth medium without FBS +<br>10 µg/mL transferrin<br>**D1-D3**<br>0.5 mM IBMX<br>25 nM dexa<br>20 nM insulin<br>200 pM T3<br>2 µM Rosi<br>100 nM cortisol<br>**D3-D25**<br>20 nM human insulin<br>200 pM T3<br><br>Inactive beige adipocytes<br>Growth medium without FBS +<br>10 µg/mL transferrin<br>**D1-D3**	mRNA (UCP1, SLC7A10/ASC-1, SHMT1, GPT2, UCP2, PM20D1, CIDEA, CITED1, CKMT1, CKMT2, CPT2)<br><br>Protein (UCP1, SLC7A10/ASC-1, SHMT1, GPT2)<br><br>OCR, ECAR (Seahorse®)	Active beige vs white and inactive beige adipocytes<br>↑ UCP1, UCP2, PM20D1, CIDEA, CITED, CKMT1, CKMT2, CPT2 mRNA<br>↑ UCP1, SLC7A10/ASC, SHMT1, GPT2, complex I, complex II, complex IV protein<br>↔ Complex III, complex V protein protein<br>↑ cAMP stimulated uncoupled respiration and ECAR<br><br>Inactive beige vs white adipocytes<br>↔ UCP1, UCP2, PM20D1, CIDEA, CITED, CKMT1, CKMT2, CPT2 mRNA<br>↑ UCP1, SLC7A10/ASC, SHMT1, GPT2, complex I, complex II, complex III, complex IV, complex V protein<br>↔ cAMP stimulated uncoupled respiration and ECAR

			0.5 mM IBMX<br>25 nM dexa<br>20 nM insulin<br>200 pM T3<br>2 µM Rosi<br>**D3-D14**<br>20 nM human insulin<br>200 pM T3<br>500 nM Rosi<br>**D14**<br>500 µM cAMP (4h)<br>**D14-D29**<br>20 nM human insulin<br>200 pM T3		
Vinnai et al., 2023<br>Hungary [21]	SVF from adipose tissue (SC and DN)<br><br>Growth medium<br>Serum free DMEM-F12<br>33 µM biotin<br>17 µM pantothenate<br>100 U/mL penicillin/streptomycin<br><br>Induction at 100% confluency	Higher browning capacity differentiation cocktail<br>Growth medium +<br>10 µg/mL transferrin<br>**D1-D3**<br>0.5 mM IBMX<br>1 µM dexa<br>850 nM insulin<br>0.2 nM T3<br>**D3-D11**<br>850 nM insulin<br>0.2 nM T3<br>0.5 µM Rosi	Regular adipogenic differentiation medium +<br>10 µg/mL transferrin<br>**D1-D3**<br>0.25 mM IBMX<br>25 nM dexa<br>0.1 µM cortisol<br>20 nM insulin<br>0.2 nM T3<br>2 µM Rosi<br>**D3-D11**<br>0.1 uM cortisol<br>20 nM insulin<br>0.2 nM T3<br><br>SC vs DN	mRNA (UCP1, PGC1α, CKMT2, CIDEA, DIO2, TBX1, CITED1)<br><br>Protein (UCP1, PGC1α)<br><br>OCR, ECAR (Seahorse®)	Higher browning capacity differentiation medium vs regular adipogenic differentiation medium<br>↑ PGC1α mRNA (SC), DIO2 mRNA (SC and DN), TBX1 mRNA (SC and DN), CITED mRNA (SC and DN)<br>↑ UCP-1 and PGC1α protein (SC and DN)<br><br>Higher browning capacity differentiation medium: DN vs SC<br>↑ UCP-1 mRNA
Wu et al., 2023<br>China, US [22]	SVF from WAT (SC)<br><br>Growth medium<br>DMEM-F12<br>10% FBS<br>1% penicillin/streptomycin<br><br>Induction at 100% confluency	**D1-D2**<br>Growth medium +<br>0.5 mM IBMX<br>1 µM dexa<br>1 µg/mL insulin<br>60 µM indomethacin<br>1 nM T3<br>1 µM Rosi<br>**D2-D6**<br>1 µg/mL insulin<br>1 nM T3<br>1 µM Rosi<br>**D6-D10**<br>5% BSA (to withdrawal beige adipogenesis stimuli)	-	mRNA (UCP1, PGC1α, PRDM16, CIDEA)<br><br>Protein (UCP1)	Detectable UCP1, PGC1α, PRDM16, CIDEA mRNA<br><br>Detectable UCP1 protein expression
Di Maio et al., 2022<br>Italy, US [23]	BM mesenchymal stromal cells (Lonza, Italy)<br><br>Growth medium<br>DMEM (low glucose)<br>10% FBS<br>3 ng/mL bFGF<br>1% penicillin/streptomycin<br>1% L-glutamine	Brown adipocyte induction medium<br>**D1-D14**<br>DMEM (high glucose)<br>1% penicillin/streptomycin<br>10% horse serum<br>0.5 mM IBMX	White adipogenic induction medium<br>**D1-D14**<br>DMEM (high glucose)<br>10% horse serum<br>1% penicillin/streptomycin<br>0.5 mM IBMX<br>1 mM dexa	mRNA (UCP1)<br><br>Protein (UCP1)<br><br>Mitochondrial content (MitoTracker®)<br><br>OCR (Seahorse®)	White adipocytes: Rosi vs vehicle<br>↑ UCP1 mRNA<br>↑ UCP1 protein<br>↑ % of UCP1-positive cells<br>↑ mitochondrial content<br>↑ uncoupled mitochondrial respiration<br><br>Brown vs white adipocytes

	Induction at 70-80% confluency	1 mM dexa<br>10 µg/mL insulin<br>200 µM indomethacin<br>**D14-D21**<br>DMEM-F12<br>10% FBS<br>20 nM insulin<br>200 µM ascorbic acid<br>0.2 nM T3<br>1 µM CL316243	10 µg/mL insulin<br>200 µM indomethacin<br>**D14-D21**<br>Previous medium + 43 nM Rosi<br><br>Rosi vs vehicle for differentiated white adipocytes		↑ UCP1 protein
Farrar et al., 2022<br>US [24]	SVF from breast adipose tissue<br><br>Growth medium<br>DMEM-F12<br>Glutamax<br>2% FBS<br>5 mM L-glutamine<br>5 ng/mL hFGF<br>5 µg/mL human insulin<br>1 µg/mL hydrocortisone<br>50 µg/mL L-ascorbic acid<br>200 IU/mL penicillin<br>0.2 mg/mL streptomycin<br>25 µg/mL amphotericin B<br>100 µg/mL normacin<br><br>Induction 2 d after confluency	Beige adipocyte<br>Growth medium +<br>**D1-D2**<br>0.5 mM IBMX<br>5 µM dexa<br>5 µg/mL insulin<br>0.5 µM Rosi<br>125 µM indomethacin<br>1 nM T3<br>**D2-D14**<br>5 µg/mL insulin<br>0.5 µM Rosi<br>1 nM T3	White adipocyte<br>Growth medium +<br>**D1-D2**<br>0.5 mM IBMX<br>1 µM dexa<br>5 µg/mL insulin<br>125 µM indomethacin<br>**D2-D14**<br>5 µg/mL insulin	mRNA (UCP1)	Beige vs white adipocyte<br>↑ UCP1 mRNA
Fu et al., 2022<br>China [25]	SVF from WAT (SC and visceral, great omentum)<br><br>Growth medium<br>DMEM-F12<br>10% FBS<br>100 U/mL penicillin/streptomycin<br><br>Confluency state at induction: NR	Beige adipocyte<br>Growth medium +<br>**D1-D14**<br>0.5 mM IBMX<br>0.1 µM dexa<br>10 µg/mL insulin<br>200 µM indomethacin<br>**D14-D18**<br>50 nM irisin	White adipocyte<br>Growth medium +<br>**D1-D18**<br>0.5 mM IBMX<br>0.1 µM dexa<br>10 µg/mL insulin<br>200 µM indomethacin<br><br>Sc vs visceral adipocytes	mRNA (UCP1, PRDM16, CIDEA)<br><br>Protein (UCP1)	Beige vs white adipocytes<br>SC<br>↑ UCP1, CIDEA, PRDM16 mRNA<br>↑ UCP1 protein<br><br>Beige vs white adipocytes<br>Visceral<br>↔ UCP1, CIDEA, PRDM16 mRNA<br>↔ UCP1 protein
Guillemet et al., 2022<br>France [26]	Pre-adipocytes (SC, overweight young female)<br><br>Growth medium<br>DMEM<br>FBS 10%<br><br>Confluency at induction: NR	Brown/beige adipocytes<br>Growth medium +<br>**D1/D10-12**<br>0.25 mM IBMX<br>0.1 µM dexa<br>50 nM insulin<br>0.1 µM Rosi<br>± 0.1 µM isoproterenol or 1 µM FSK (last 2 h)	Non-differentiated pre-adipocytes	Protein (UCP1)	Brown/beige adipocytes vs non-differentiated pre-adipocytes<br>↑ UCP1 protein<br><br>Brown/beige adipocytes stimulated vs non-stimulated with isoproterenol or FSK: no data for thermogenic markers
He et al., 2022<br>Germany, India, Sweden [27]	SVF and adipocytes from WAT (abdominal SC)<br><br>Growth medium<br>Adipocytes (ceiling culture)<br>DMEM (high glucose)<br>10% NCS	Beige adipocytes<br>Growth medium +<br>**D1-D3 (induction)**<br>0.5 mM IBMX<br>10 µM dexa<br>0.5 µg/mL insulin<br>2 nM T3	Ceiling culture (dedifferentiated adipocytes from mature adipocytes)<br>Growth medium<br>DMEM (high-glucose)<br>10% NCS<br>4 mM L-glutamine	mRNA (UCP1, PRDM16)<br><br>Protein (UCP1)	Beige adipocytes<br>Detectable UCP1 and PRDM16 mRNA<br>Detectable UCP1 protein<br><br>Beige adipocytes vs ceiling culture + Rosi<br>↔ UCP1 mRNA<br>↓ PRDM16 mRNA

	4 mM L-glutamine<br>50 U/mL penicillin<br>50 U/mL streptomycin<br>SVF frin adipose tissue Human ADSCs medium (Cyagen Biosciences)<br>10% FBS<br>2 mM L-glutamine<br>50 U/mL penicillin<br>50 U/mL streptomycin<br><br>Induction at 100% confluency	1 µM Rosi<br>**D4 (maintenance)**<br>Growth media<br>0.5 µg/mL insulin<br>(cycles of induction and maintenance were repeated three to four times, totaling 12 to 16 days)<br>2 µM CL316,243 (8-12 h before harvesting)	50 U/mL penicillin<br>50 U/mL streptomycin<br>± 1 uM Rosi		↔ UCP1 protein
Herbers et al, 2022<br>Finland [28]	SVF from WAT (abdominal SC)<br><br>Growth medium<br>DMEM-F12<br>5% human serum<br>1% penicillin/streptomycin<br>1% GlutaMax<br><br>Induction 2 d after confluency	Adipocyte differentiation<br>DMEM-F12<br>3% human serum<br>1% penicillin/streptomycin<br>33 µM biotin<br>17 µM pantothenate<br>**Protocol 1 (P1)**<br>**D1-D7**<br>0.5 mM IMBX<br>1 µM dexa<br>100 nM insulin<br>1 µM Rosi<br>2 nM T3<br>10 µg/mL transferrin<br>**D7-D21**<br>1 µM dexa<br>100 nM insulin<br>**Protocol 2 (P2)**<br>**D1-D7**<br>0.5 mM IMBX<br>1 µM dexa<br>100 nM insulin<br>1 µM Rosi<br>**D7-D21**<br>1 µM dexa<br>100 nM insulin<br>**Protocol 3 (P3)**<br>**D1-D7**<br>0.5 mM IMBX<br>1 µM dexa<br>100 nM insulin<br>100 nM Rosi<br>**D7-D21**<br>1 µM dexa<br>100 nM insulin<br>**Protocol 4 (P4)**<br>**D1-D7**<br>0.5 mM IMBX<br>1 µM dexa<br>850 nM insulin<br>125 µM indomethacin<br>**D7-D21**<br>Medium only	Differentiated vs non-differentiated cells<br><br>Different differentiation protocols	mRNA (UCP1, UCP2, CIDEA)<br><br>Protein (UCP1)<br><br>Mitochondrial content (mtDNA, mitochondrial number/TEM)<br><br>OCR (Seahorse®)	UCP1, UCP2, CIDEA mRNA<br>↑ P4 vs P1, P2, P3, P4, non-differentiated cells<br>↔ P1, P2, P3 vs non-differentiated cells<br>PGC1α mRNA<br>↑ P4 vs P3<br>PGC1β mRNA<br>↑ P4 vs P2<br><br>UCP1 protein<br>↑ P4 vs P1, P2, P3, P4, non-differentiated cells<br>↔ P1, P2, P3 vs non-differentiated cells<br><br>↑ mtDNA (P1, P4 vs non-differentiated cells)<br><br>↑ Mitochondrial number (P4 vs non-differentiated cells)<br><br>OCR<br>↔ Basal respiration (P1 vs P2 vs P3 vs P4 vs P5)<br>↔ ATP production (P1 vs P2 vs P3 vs P4 vs P5)<br>↑ Maximal respiration (P1 vs P3, P5)<br>↑ Proton leak (P1 vs P3)<br>↑ Spare capacity (P1 vs P3, P5)<br><br>↑ Coupling efficiency (P3 vs P1, P4)

Lin et al., 2022<br>China [29]	SVF from omental adipose tissue<br><br>Growth medium<br>DMEM-F12<br>10% FBS<br>1% penicillin/streptomycin<br><br>Induction 2 d after confluency	Beige adipocytes medium +<br>10 µg/mL transferrin<br>**D1-D3**<br>0.5 mM IMBX<br>1 µM dexa<br>850 nM insulin<br>0.2 nM T3<br>**D3-D14**<br>850 nM insulin<br>0.2 nM T3<br>0.5 µM Rosi	Beige adipocytes vs non-differentiated cells from SVF from omental adipose tissue	Protein (UCP1, PRDM16)<br><br>Mitochondrial content (MitoTracker®)	Beige adipocytes vs non-differentiated cells from SVF from omental adipose tissue<br>↑ UCP1, PRDM protein<br>↑ mitochondrial content
Nagy et al., 2022<br>Hungary [30]	SVF cells from pericardial adipose tissue<br><br>Growth medium<br>DMEM-F12-Ham's<br>10% FBS<br>1% penicillin/streptomycin<br>33 µM biotin<br>17 µM pantothenate<br><br>Induction at 100% confluency	Beige adipocytes<br>Serum-free growth medium +<br>10 µg/mL transferrin<br>**D1-D3**<br>0.5 mM IMBX<br>1 µM dexa<br>850 nM insulin<br>0.2 nM T3<br>**D3-D14**<br>850 nM insulin<br>0.2 nM T3<br>0.5 µM Rosi	White adipocytes<br>Serum-free growth medium +<br>10 µg/mL transferrin<br>**D1-D3**<br>0.5 mM IMBX<br>25 nM dexa<br>20 nM insulin<br>200 pM T3<br>2 µM Rosi<br>**D3-D14**<br>200 pM T3<br>20 nM insulin<br>100 nM hydrocortisone	mRNA (UCP1, TBX1)<br><br>Protein (UCP1, PGC1α, TOMM20a)<br><br>OCR (Seahorse®)	Beige vs white adipocytes<br>ns ↑ UCP1 and TBX1 mRNA<br>↑ UCP1 protein<br>↔ PGC1α protein<br>↑ TOMM20a (mitochondrial content)<br>↑ uncoupled mitochondrial respiration
Ngono Ayissi et al., 2022<br>France [31]	SVF from WAT (SC)<br><br>Growth medium<br>DMEM (high-glucose)<br>10% FBS<br>2 mM glutamine<br>100 U/mL penicillin/streptomycin<br>HEPES 1 uM<br><br>Confluency state at induction: NR	Beige adipocyte<br>Growth medium +<br>**D1-D7**<br>0.5 mM IBMX<br>1 µM dexa<br>1000 nM insulin<br>1 µM Rosi<br>50 µM indomethacin<br>2 nM T3<br>**D7-D14**<br>1000 nM insulin<br>1 µM Rosi<br>2 nM T3	White adipocyte<br>Growth medium +<br>**D1-D7**<br>500 uM IBMX<br>1 µM dexa<br>1 µM insulin<br>1 uM Rosi<br>**D7-D14**<br>1 µM insulin	mRNA (UCP1, TMEM26, CD137, PRDM16, CITED1, DIO2)<br><br>Protein (PGC1α)<br><br>OCR (Seahorse®)	Beige vs white adipocytes<br>↑ CD137, PRDM16, DIO2, UCP1 mRNA<br>↔ TMEM26, CITED1 mRNA<br>↑ PGC1a protein<br>↑ basal respiration, proton leak and maximal respiration
Niemann et al., 2022<br>Germany [32]	SVF from adipose tissue (supraclavicular) and MADSCs<br><br>Growth medium<br>DMEM-F12<br>10% FBS<br>1% penicillin/streptomycin<br>1 nM acidic FGF1<br><br>Induction 2d after confluency	Growth medium without FGF1 +<br>10 µg/mL transferrin<br>**D1-D3**<br>0.54 mM IBMX<br>0.1 µM dexa<br>100 nM insulin<br>2 nM T3<br>0.2 µM Rosi<br>**D3-D12**<br>0.1 µM dexa<br>100 nM insulin<br>2 nM T3<br>0.2 µM Rosi	-	mRNA (UCP1)<br><br>OCR (Seahorse®)	Detectable UCP1 mRNA<br>Detectable OCR

Park et al, 2022<br>South Korea [33]	ADSCs (Biosolution)<br><br>Growth medium<br>MEM-alpha<br>10% FBS<br>100 U/mL penicillin<br>100 µg/mL streptomycin<br><br>Induction 2 d after confluency	DMEM-F12<br>18.5 mM glucose<br>15 mM HEPES<br>25 mM $NaHCO_3$<br>100 units/mL penicillin<br>100 µg/mL streptomycin<br>33 µM biotin<br>17 µM pantothenate<br>**D1-D7**<br>0.5 mM IBMX<br>0.1 µM dexa<br>100 nM insulin<br>1000 nM T3<br>1 µM Rosi<br>**D7-D15**<br>100 nM insulin<br>1000 nM T3<br>± NE (concentration NR) for 6 h before harvesting	Adipocytes stimulated vs non-stimulated with NE	mRNA (UCP1, PGC1α, ELOVL3, ELOVL6, DIO2, CIDEA CIDEC, PPARα, EBF2, CITED1, TBX1, SHOX2, TMEM26, P2RX5, PAT2, CAR4)<br><br>Protein (UCP1, PGC1α)<br><br>Mitochondrial content (MitoTracker®, mtDNA)<br><br>OCR (Seahorse®)	Adipocytes stimulated vs non-stimulated with NE<br>↑ UCP1, PGC1α, ELOVL3, ELOVLl6, DIO2, CIDEA, CIDEC, CITED1, TBX1, SHOX2, TMEM26, P2RX5, PAT2, CAR4 mRNA<br>↔ PPARα, EBF2 mRNA<br>↑ UCP1, PGC1α protein<br>↑ mitochondrial content<br>↑ OCR (basal, ATP-production, maximal, proton leak)
Park et al., 2022<br>South Korea [34]	ADSCs (commercial)<br><br>Growth medium<br>DMEM-F12-Ham's<br>10% FBS<br>1% penicillin/streptomycin<br><br>Induction 2 d after confluency	Beige differentiation<br>Growth medium +<br>**D1-D7**<br>0.5 mM IBMX<br>0.1 µM dexamethasone<br>100 nM insulin<br>2 nM T3<br>1 µM Rosi<br>**D7-D12**<br>2 nM T3<br>1 µM Rosi	White differentiation<br>Growth medium +<br>**D1-D7**<br>0.5 mM IBMX<br>100 nM dexamethasone<br>100 nM insulin<br>2 nM T3<br>1 µM Rosi<br>**D7-D12**<br>2 nM T3	mRNA (UCP1, CIDEA, PGC1α, ELOVL3, CIDEC, PAT2, SLC25A20, FABP3, PDK4, CITED1, SAMM50)<br><br>Protein (SAMM50)	Beige vs white adipocyte<br>↑ UCP1, CIDEA, PGC1α, ELOVL3, ELOVL16, CIDEC, PAT2, SLC25A20, FABP3, PDK4, CITED1, SAMM50 mRNA<br><br>↑ SAMM50 protein
Porras et al, 2022<br>US [35]	Microvascular fragments from adipose tissue<br><br>Growth medium<br>DMEM<br>20% FBS<br>1% penicillin/streptomycin<br><br>Confluency at induction: NR	Beige adipocytes<br>DMEM-F12<br>20% FBS<br>1% penicillin/streptomycin<br>**D1-D4**<br>1 µM dexa<br>10 µg/mL insulin<br>1 µM Rosi<br>10 µM FSK<br>120 nM T3<br>**D4-D15**<br>10 µg/mL insulin	White adipocytes<br>DMEM-F12<br>20% FBS<br>1% penicillin/streptomycin<br>**D1-D4**<br>1 µM dexa<br>10 µg/mL insulin<br>125 µM indomethacin<br>10 µM FSK<br>**D4-D15**<br>10 µg/mL insulin	mRNA (UCP1, CIDEA, PGC1α, COX7A)<br><br>OCR, ECAR (Seahorse®)	Beige vs white adipocytes<br>↑ UCP1, CIDEA, PGC1α, COX7A mRNA<br>↑ maximal respiration<br>↔ basal respiration, proton leak, ATP production, spare capacity, non-mitochondrial oxygen consumption, uncoupling efficiency
Takeda et al., 2022<br>Japan [36]	Dermal fibroblasts (35-54 year-old subjects)<br><br>Growth medium<br>DMEM (high glucose)<br>10% FBS<br>100 U/mL penicillin/streptomycin<br><br>Induction at 80-90% confluency	Brown adipocytes<br>Growth medium without FBS +<br>**D1-D21**<br>0.03 mM IBMX<br>0.5 µM dexa<br>6.6 µg/mL insulin<br>100 nM T3	Fibroblasts differentiated without Rosi, FSK, and BMP7	mRNA (UCP1)<br><br>Protein (UCP1)<br><br>OCR (Seahorse®)	Brown adipocytes<br>↑ UCP1 mRNA<br>↑ UPC1 protein<br>↑ OCR (basal, proton leak, maximal, ATP production, spare capacity)

		100 µg/mL ascorbic acid<br>1 mg/mL linoleic acid, oleic acid albumin<br>1 µM Rosi<br>7.5 uM FSK<br>20 µg/mL BMP7			
Vámos et al., 2022<br>Hungary [37]	SVF from WAT (SC)<br><br>Growth medium<br>DMEM-F12<br>10% FBS<br>33 µM biotin<br>17 µM pantothenate<br>100 U/mL penicillin/streptomycin<br><br>Induction at 100% confluency	Beige adipocyte<br>Growth medium without FBS +<br>10 µg/mL transferrin<br>**D1-D4**<br>0.5 mM IBMX<br>1 µM dexa<br>850 nM insulin<br>0.2 nM T3<br>**D4-D14/D21/D28**<br>850 nM insulin<br>0.2 nM T3<br>0.5 µM Rosi	White adipocyte<br>Growth medium without FBS +<br>10 µg/mL transferrin<br>**D1-D4**<br>0.5 mM IBMX<br>25 nM dexa<br>100 nM cortisol<br>20 nM insulin<br>2 µM Rosi<br>200 pM T3<br>**D4-D14/D21/D28**<br>100 nM cortisol<br>20 nM insulin<br>200 pM T3<br><br>Beige to white transition<br>**D1-D14:** beige adipocyte differentiation medium<br>**D14-D28:** white adipocyte differentiation medium	mRNA (UCP1, CIDEA, PGC1α)<br><br>Protein (UCP1)<br><br>Mitochondrial content (mtDNA)<br><br>OCR, ECAR (Seahorse®)	Beige vs white adipocyte<br>↑ UCP1 and CIDEA mRNA<br>↔ PGC1α mRNA<br>↑ UCP1 protein<br>↑ mitochondrial DNA content<br>↑ OCR (basal, stimulated, proton leak)<br>↑ ECAR (basal, stimulated)<br><br>Beige to white transition<br>↓ UCP1 and CIDEA mRNA<br>↓ UCP1 protein<br>↓ mitochondrial content<br>↓ OCR (basal)<br>↔ OCR (stimulated, proton leak)<br>↓ ECAR (basal, stimulated)
Bokhari et al., 2021<br>France, Sweden, US [38]	MADSCs<br><br>Growth medium<br>DMEM<br>10% FBS<br>2.5 ng/mL FGF2<br>100 IU/mL penicillin<br>100 µg/mL streptomycin<br>10 mM HEPES<br><br>Induction 2 d postconfluency	DMEM-F12-Ham's<br>10% FBS<br>100 IU/mL penicillin<br>100 µg/mL streptomycin<br>10 mM HEPES<br>10 µg/mL transferrin<br>**D1-D2**<br>0.5 mM IBMX<br>1 µM dexa<br>10 nM insulin<br>0.2 nM T3<br>**D2-D21**<br>10 nM insulin<br>0.2 nM T3<br>**D2-D9, D14-D18:** Rosi (concentration NR)<br>+ 1 µM CL316243 (acute stimulation)<br>+ noradrenaline (concentration NR, acute stimulation)	Adipocytes not stimulated with CL216243 or noradrenaline	Heat production (isothermal microcalorimetry)	Stimulated vs non-stimulated adipocytes (CL316,243 or noradrenaline)<br>↑ heat flow and accumulated heat
Cero et al, 2021<br>US [39]	SVF from adipose tissue (superficial neck and supraclavicular)<br><br>Growth medium	Adipocyte differentiation<br>Growth medium without FBS	Adipocytes from SVF from superficial neck vs supraclavicular adipose tissue	mRNA (UCP1, PGC1α, PRDM16, ZIC1, DIO2, MTUS1, TBX1, TMEM26, ADRB3)	Adipocytes from SVF from supraclavicular vs superficial neck adipose tissue<br>↑ UCP1, PGC1α, PRDM16, ZIC1, DIO2, MTUS1, TBX1, TMEM26, ADRB3 mRNA

	DMEM-F12<br>10% FBS<br>1% penicillin/streptomycin<br><br>Induction at 100% confluency (cells treated with 4 ng/mL bFGF for 2 days before confluency)	3% human serum<br>1% penicillin/streptomycin<br>33 µM biotin<br>17 µM pantothenate<br>10 µg/mL transferrin<br>**D1-D10**<br>0.5 mM IBMX<br>0.1 µM dexa<br>100 nM insulin<br>5 µM Rosi<br>2 nM T3<br>34 µM BMP7<br>**D10-D20/21**<br>10 nM dexa<br>10 nM insulin	Adipocytes vs non-differentiated cells from SVF from supraclavicular adipose tissue	Protein (UCP1)	↑ UCP1 protein<br><br>Adipocytes vs non-differentiated cells from SVF from supraclavicular adipose tissue<br>↑ UCP1, PGC1α, PRDM16, ZIC1, DIO2, MTUS1, TBX1, TMEM26, ADRB3 mRNA<br>↑ UCP1 protein
Di Maio et al., 2021<br>Italy [40]	BM mesenchymal stromal cells<br><br>Growth medium<br>MEM alpha<br>10% FBS<br>3 ng/mL bFGF<br><br>Induction at 70-80% confluency	Brown adipocytes<br>**D1-14**<br>DMEM (high glucose)<br>10% FBS<br>1% penicillin/streptomycin<br>0.5 mM IBMX<br>1 mM dexa<br>10 µg/mL insulin<br>200 µM indomethacin<br>**D14-D21**<br>DMEM-F12<br>10% FBS<br>20 nM insulin<br>0.2 nM T3<br>200 µM ascorbic acid<br>1 µM CL316,243	White adipocytes<br>**D1-14**<br>DMEM (high glucose)<br>10% FBS<br>1% penicillin/streptomycin<br>0.5 mM IBMX<br>1 mM dexa<br>10 µg/mL insulin<br>200 µM indomethacin	Lipid droplet size<br><br>mRNA (UCP1)<br><br>Oxygen consumption (MitoXpress Xtra assay, Luxcel Biosciences®)	Brown vs white adipocytes<br>↓ mean lipid droplet size<br>↑ UCP1 mRNA<br>↓ ATP production<br>↑ Proton leak
Jiao et al., 2021<br>China [41]	ADSCs (SC WAT)<br><br>Growth medium: NR<br><br>Induction at 80% confluency and after 8 h starvation	**D1-D8**<br>Growth medium +<br>0.5 mM IBMX<br>0.1-0.4 M dexa<br>10 µg/mL insulin<br>2 µM indomethacin	-	mRNA (CIDEA, UCP1, PRDM16)<br><br>Protein (CIDEA)	From D0 to D8<br>↑ CIDEA, UCP1, and PRDM16 mRNA<br>↑ CIDEA, UCP1, and PRDM16 mRNA
Nascimento et al., 2021<br>Netherlands, Switzerland [42]	SVF from adipose tissue (cervical and SC)<br><br>Growth medium: NR<br><br>Induction at 100% confluency	SVF from cervical adipose tissue<br>33 µM biotin<br>17 µM pantothenate<br>10 µg/mL transferrin<br>**D1-D7**<br>0.25 mM IBMX<br>0.1 µM dexa<br>100 nM insulin<br>5 µM Rosi<br>2 nM T3<br>**D7- until lipid accumulation**<br>0.1 µM dexa<br>100 nM insulin	SVF from SC adipose tissue<br>33 µM biotin<br>17 µM pantothenate<br>10 µg/mL transferrin<br>**D1-D7**<br>0.25 mM IBMX<br>100 nM dexa<br>100 nM insulin<br>5 µM Rosi<br>2 nM T3<br>**D7- until lipid accumulation**<br>100 nM dexa<br>100 nM insulin<br>2 nM T3	mRNA (UCP1, PGC1α)<br><br>OCR (Seahorse®)	Adipocytes differentiated from cervical and SC adipose tissue<br>Detectable UCP1 and PGC1α mRNA<br><br>Cervical vs SC<br>↑ uncoupled respiration

		2 nM T3			
Tsagkaraki et al., 2021<br>US [43]	SVF from WAT (SC)<br><br>Growth medium<br>EGM2-MV<br><br>Induction at 100% confluency	NRIP1 disruption in preadipocytes (CRISPR-based)<br>DMEM<br>10% FBS<br>Penicillin/streptomycin<br>**D1-D3**<br>0.5 mM IBMX<br>1 µM dexa<br>1 µg/mL insulin<br>**D3-D10**<br>DMEM<br>10% FBS	Preadipocytes without NRIP1 disruption<br>DMEM<br>10% FBS<br>Penicillin/streptomycin<br>**D1-D3**<br>0.5 mM IBMX<br>1 µM dexa<br>1 µg/mL insulin<br>**D3-D10**<br>DMEM<br>10% FBS	mRNA (UCP1)<br><br>Transcriptome/RNA seq<br><br>Protein (UCP1)	NRIP1 disruption vs no disruption<br>↑ UCP1 mRNA<br>↑ UCP1 protein<br>Different protein coding genes expression (upregulation of genes related to cellular respiration and thermogenesis)
Xia et al., 2021<br>China [44]	Mature white adipocytes from WAT (SC)<br><br>Cells grown in beige adipocyte induction medium<br><br>Confluency at induction: NR	Beige adipocyte<br>**D1-D3 (ceiling culture)**<br>DMEM (high-glucose)<br>10% NCS<br>2.4 nM insulin<br>25 µg/mL sodium ascorbate<br>10 mM HEPES<br>4 mM L-glutamine<br>50 IU/mL penicillin<br>50 µg/mL streptomycin<br>1 µM Rosi	Mature white adipocytes not treated with beige adipocyte induction medium	mRNA (UCP1, PRDM16, P2RX5)<br><br>Mitochondrial content (electron microscopy)	Induced beige adipocytes vs white adipocytes<br>↑ mitochondrial content<br>↑ UCP1, PRDM16, P2RX5 (mRNA)
Halbgebauer et al., 2020<br>Germany [45]	SVF from breast adipose tissue<br><br>Growth medium<br>DMEM-F12-Ham's<br>10% FCS<br>15 mM HEPES<br>15 mM $NaHCO_3$<br>33 µM biotin<br>17 µM pantothenate<br>50 µg/mL gentamicin<br><br>Induction at 80% confluency	Rosi-induced adipocytes<br>Growth medium +<br>10 µg/mL transferrin<br>**D1-D4**<br>0.25 mM IBMX<br>25 nM dexa<br>20 nM insulin<br>100 nM cortisol<br>0.2 nM T3<br>2 µM Rosi<br>**D4-D14**<br>0.25 mM IBMX<br>20 nM insulin<br>100 nM cortisol<br>0.2 nM T3	Indomethacin induced adipocytes<br>Growth medium +<br>10 µg/mL transferrin<br>**D1-D4**<br>0.25 mM IBMX<br>25 nM dexa<br>20 nM insulin<br>100 nM cortisol<br>0.2 nM T3<br>100 µM indomethacin<br>**D4-D14**<br>0.25 mM IBMX<br>20 nM insulin<br>100 nM cortisol<br>0.2 nM T3	mRNA (UCP1, CIDEA, PRMD16)<br><br>Protein (UCP1)<br><br>OCR (Seahorse®)	Rosi vs indomethacin-induced adipocytes<br>↑ UCP1, CIDEA, PRDM16 mRNA<br><br>↑ protein UCP1<br><br>↑ OCR (basal, proton leak, maximal, and cAMP-stimulated)
Kroon et al., 2020<br>Sweden, US [46]	SVF from adipose tissue (abdominal SC)<br><br>Growth medium<br>Proliferation medium 1 (Zenbio)<br>3% FBS Gold<br>1% penicillin/streptomycin<br>1 nM bFGF	Brown adipocytes<br>Basal medium 1 (ZenBio)<br>3% FBS Gold<br>1% penicillin/streptomycin<br>1 nM bFGF<br>**D1-D7**	White adipocytes<br>Basal medium 1 (ZenBio)<br>3% FBS Gold<br>1% penicillin/streptomycin<br>1 nM bFGF<br>**D1-D7**<br>0.5 mM IBMX<br>100 nM dexa	mRNA (UCP1, CIDEA)	Brown vs white adipocytes<br>↑ UCP1 and CIDEA mRNA

	Induction at 100% confluency	0.5 mM IBMX<br>0.1 µM dexa<br>20 nM insulin<br>1 nM T3<br>10 ng/mL BMP4<br>1 µM Rosi<br>**D7-D12**<br>0.1 µM dexa<br>20 nM insulin<br>1 nM T3<br>10 ng/mL BMP4<br>1 µM Rosi	20 nM insulin<br>1 nM T3<br>10 ng/mL BMP4<br>**D7-D12**<br>100 nM dexa<br>20 nM insulin<br>1 nM T3<br>10 ng/mL BMP4		
Li et al., 2020<br>China [47]	SVF from WAT (SC)<br><br>Growth medium<br>DMEM-F12-Ham's<br>10% FBS<br>1% penicillin/streptomycin<br>0.01% FGF<br><br>Confluency at induction: NR	Brown adipocytes<br>Growth medium +<br>White adipocytes<br>Growth medium +<br>**D1-D3**<br>0.5 mM IBMX<br>1 µM dexa<br>850 nM insulin<br>1 µM Rosi<br>1 nM T3<br>**D3-D12**<br>0.5 mM IBMX<br>850 nM insulin<br>1 µM Rosi<br>1 nM T3<br>1 nM CL316,243	White adipocytes<br>Growth medium +<br>**D1-D3**<br>0.5 mM IBMX<br>1 µM dexa<br>0.85 µM insulin<br>200 µM indomethacin<br>**D3-D12**<br>0.5 mM IBMX<br>1 µM dexa<br>0.85 µM insulin<br>200 µM indomethacin	mRNA (UCP1, PRMD16, PGC1α, CIDEA)<br><br>Protein (UCP1)<br><br>OCR (Seahorse®)	Brown vs white adipocytes<br>↑ UCP1, PRMD16, PGC1α, and CIDEA mRNA<br>↑ UCP1 protein<br>↑ OCR
Markan et al., 2020<br>US [48]	Mesenchymal stromal cells (BM, SC WAT, umbilical cord)<br><br>Growth medium<br>MEM-alpha<br>15% FBS<br>1% penicillin/streptomycin<br>1% L-glutamine<br><br>Induction at 70-80% confluency	DMEM (high glucose)<br>15% FBS<br>1% penicillin/streptomycin<br>**D1-D10**<br>0.5 mM IBMX<br>1 µM dexa<br>5 µg/mL insulin	Non-differentiated cells	mRNA (TBX1)	↑ TBX1 mRNA
Michurina et al., 2020<br>Russia [49]	SVF from WAT (abdominal SC)<br><br>Growth medium<br>DMEM-F12<br>10% FBS<br>Penicillin/streptomycin<br>2 mM glutamine<br><br>Induction at 80% confluency	Beige adipocyte<br>**D1-D21**<br>Growth medium +<br>0.25 mM IMBX<br>0.5 mM dexa<br>0.5 µg/mL insulin<br>1 µM Rosi<br>1 nM T3<br>100 µM isoproterenol	White adipocyte<br>**D1-D2**<br>Growth medium +<br>10% NCS<br>**D2-D21**<br>Growth medium +<br>10% FBS<br>0.25 uM IMBX<br>0.5 mM dexa<br>100 nM insulin<br>2 uM Rosi	mRNA (UCP1, PGC1α)<br><br>Protein (UCP1)	Beige vs white adipocytes<br>↑ UCP1 mRNA<br>↔ PGC1α mRNA<br>↑ UCP1
Moon et al., 2020<br>South Korea [50]	ADSCs (Lonza)<br><br>Growth medium<br>DMEM-F12	Brown adipocytes<br>Growth medium +<br>**D1-D4**<br>0.5 mM IBMX	White adipocytes<br>Growth medium +<br>**D1-D4**<br>0.5 mM IBMX	mRNA (UCP1)<br><br>Protein (UCP1, PRDM16)	Brown and white adipocytes<br>Detectable UCP1 mRNA<br><br>Brown vs white adipocytes

	10% FBS<br><br>Induction at 100% confluency	2 µg/mL dexa<br>5 µg/mL insulin<br>0.5 µM Rosi<br>1 nM T3<br>125 µM indomethacin<br>**D4-D10/12**<br>5 µg/mL insulin<br>1 µM Rosi<br>1 nM T3	2 µg/mL dexa<br>5 µg/mL insulin<br>0.5 µM Rosi<br>1 nM T3<br>125 µM indomethacin<br>**D4-D10/12**<br>10 µg/mL insulin		↑ UCP1 and PRMD16 protein
Nascimento et al., 2020<br>Germany, Netherlands [51]	SVF from adipose tissue (DN/BAT and WAT/depot NR)<br><br>Growth medium: NR<br><br>Induction at 100% confluency	Differentiation of SVF from BAT<br>Growth medium +<br>33 µM biotin<br>17 µM pantothenate<br>**D1-D7**<br>0.25 mM IBMX<br>0.1 µM dexa<br>100 nM insulin<br>5 µM Rosi<br>2 nM T3<br>**D7-D12**<br>0.1 µM dexa<br>100 nM insulin<br>2 nM T3	Differentiation of SVF from WAT<br>Growth medium +<br>33 µM biotin<br>17 µM pantothenate<br>**D1-D7**<br>0.25 mM IBMX<br>100 nM dexa<br>100 nM insulin<br>5 µM Rosi<br>2 nM T3<br>**D7-D12**<br>100 nM dexa<br>100 nM insulin<br>2 nM T3	OCR (Seahorse®)	Adipocytes derived from BAT vs WAT<br>↑ NE induced OCR
Qiu et al., 2020<br>China [52]	SVCs from WAT (SC)<br><br>Growth medium<br>DMEM-F12<br><br>Induction at 100% confluency	Grown medium +<br>**D1-D2**<br>10% FCS<br>0.5 mM IMBX<br>5 µM dexa<br>5000 nM insulin<br>1 µM Rosi<br>1 nM T3<br>125 µM indomethacin<br>DMEM/F-12 medium<br>**D2- D14**<br>5000 nM insulin<br>1 µM Rosi<br>1 nM T3<br><br>**D1-D14**<br>± 1 µM NE, cells collected at days D 0, 2, 5, 8, 11, 14	Normal weight vs overweight donors<br><br>Control vs NE<br><br>Differetiated (D0) vs undifferentiated cells (D14)	mRNA (PGC1α, PRDM16, UCP1)<br><br>Protein (UCP1)<br><br>OCR (Seahorse®)	From D0 to D14<br>↑ PGC1α, PRDM16, and UCP1 mRNA (higher in normal-weight and NE treatment)<br><br>Normal weight vs overweight<br>↑ UCP-1 protein induction with NE<br>↑ OCR
Saha et al., 2020<br>US [53]	Primary preadipocytes (SC, Zen-bio)<br><br>Growth medium<br>DMEM-F12<br>10% FBS<br>100 IU/mL penicillin<br>100 ug/mL streptomycin<br><br>Induction at 100% confluency	Brown adipocytes<br>Growth medium +<br>**D1-D8**<br>0.25 mM IBMX<br>0.5 µM dexa<br>100 nM insulin<br>3 µM Rosi<br>**D8-D12**<br>1 µM Rosi	White adipocytes<br>Growth medium +<br>**D1-D8**<br>0.25 mM IBMX<br>500 nM dexa<br>100 nM insulin<br>3 uM Rosi<br>**D8-D12**<br>Vehicle	mRNA (UCP1)	Brown vs white adipocytes<br>↑ UCP1 mRNA

Singh et al., 2020<br>US [54]	ADSCs (Thermo Fisher and Lonza)<br><br>Growth medium<br>DMEM<br>10% FBS<br>Antibiotic-antimycotic (Corning)<br>MEM non-essential amino acids (Corning)<br>Glutagro (Corning)<br>BME (Thermo Fisher)<br><br>Induction at 100% confluency	Beige adipocytes<br>**D1-D21**<br>DMEM-F12<br>2% probumin bovine serum albumin<br>Antibiotic-antimycotic<br>MEM non-essential amino acids<br>Trace elements A, B, and C (Corning)<br>50 µg/mL ascorbic acid<br>10 µg/mL transferrin<br>0.1 mM 2-mercaptoethanol<br>Glutagro<br>200 ng/mL LONG R3 human IGF-1<br>8 ng/mL bFGF<br>100 ng/mL BMP7<br>10 µM Y27632<br>0.5 mM IBMX<br>1 µM dexa<br>2 µM Rosi<br>1000 nM T3	White adipocytes<br>**D1-D21**<br>StemPro adipogenesis differentiation kit (Thermo Fisher)	mRNA (UCP1)<br><br>Protein (UCP1)<br><br>OCR (basal and FSK-stimulated, Seahorse®)	Beige vs non-differentiated ADSCs<br>↑ UCP1 mRNA<br><br>Beige vs white adipocytes<br>↑ OCR (basal and FSK-stimulated)<br>↑ UCP1 (protein)
Szatmári-Tóth et al., 2020<br>Hungary [55]	SVF from WAT (abdominal SC)<br><br>Growth medium<br>DMEM-F12<br>10% FBS<br>100 IU/mL penicillin/streptomycin<br>33 uM biotin<br>17 uM pantothenate<br><br>Induction at 100% confluency	Beige adipocytes<br>Serum-free growth medium +<br>10 µg/mL transferrin<br>**D1-D4**<br>0.5 mM IMBX<br>1 µM dexa<br>850 nM insulin<br>0.2 nM T3<br>**D4-D14**<br>0.5 µM Rosi<br>850 nM insulin<br>0.2 nM T3<br>± 500 µM cAMP (6, 10, 14 h before harvesting)	White adipocytes<br>Serum-free growth medium +<br>10 µg/mL transferrin<br>**D1-D4**<br>500 µM IMBX<br>25 nM dexa<br>20 nM insulin<br>2 µM Rosi<br>200 pM T3<br>100 nM cortisol<br>**D4-D14**<br>20 nM insulin<br>200 pM T3<br>100 nM cortisol<br>± 500 µM cAMP (6, 10, 14 h before harvesting)	mRNA (UCP1, PGC1α)<br><br>Protein UCP1<br><br>Mitochondrial content (mtDNA)	Beige vs white adipocytes<br>Similar basal and cAMP stimulated UCP1, PGC1α mRNA<br>Similar basal and cAMP stimulated mitochondrial content<br><br>↑ UCP1 basal and cAMP-stimulated protein
Tóth et al., 2020<br>Hungary [56]	SVF from WAT (SC)<br><br>Growth medium<br>DMEM-F12-Ham's<br>10% FBS<br>10% EGM2<br>1% biotin<br>1% pantothenate<br>1% streptomycin-penicillin<br><br>Confluency at induction: NR	Brown adipocyte<br>Growth medium without EGM2 +<br>**D1-D14**<br>850 nM insulin<br>0.5 µM Rosi<br>3 nM T3<br>10 µg/mL transferrin	White adipocyte<br>Growth medium without EGM2 +<br>**D1-D4**<br>0.25 mM IBMX<br>25 nM dexa<br>Hydrocortisone (concentration NR)<br>20 nM insulin<br>2 µM Rosi<br>3 nM T3<br>10 µg/mL transferrin<br>**D4-D14**<br>Hydrocortisone (concentration NR)	Transcriptome/RNA seq (ProFAT and BATLAS)	Brown vs white adipocyte<br>Different protein coding genes expression (brown vs white markers)

			20 nM insulin 20 nM<br>3 nM		
Tran et al., 2020<br>Denmark, UK, US [57]	Adipose tissue biopsies (supraclavicular and abdominal SC; carotid perivascular and neck SC)<br><br>SVF from adipose tissue (supraclavicular and abdominal SC)<br>Growth medium<br>DMEM-F12<br>10% FBS<br>1% penicillin-streptomycin<br>1 nM FGF1<br>Induction: 2 d after confluency<br><br>Cell suspensions from capillary growth from adipose explants (carotid perivascular and neck SC)<br>Growth medium<br>EGM2-MV<br><br>Confluency at induction: NR	SVF from adipose tissue<br>Growth medium without FBS and FGF1 +<br>10 µg/mL transferrin<br>**D1-D3**<br>0.54 mM IBMX<br>0.1 µM dexa<br>100 nM insulin<br>0,2 µM Rosi<br>2 nM T3<br>**D3-D12**<br>0.1 µM dexa<br>100 nM insulin<br>0.2 µM Rosi<br>2 nM T3<br>± 10 µM NE for 4 h before cell harvesting<br><br>Progenitor cells from adipose explants<br>DMEM<br>10% FBS<br>**D1-D3**<br>0.5 mM IBMX<br>1 µM dexa<br>1 µg/mL insulin<br>**D3-D10**<br>DMEM<br>10% FBS<br>± 10 µM FSK for 6 h before cell harvesting<br>Medium change: 50% every other day	SVF from adipose tissue<br>Supraclavicular vs abdominal SC<br><br>Carotid perivascular and neck SC: adipocyte vs progenitor, treatment with FSK vs no treatment with FSK	mRNA (UCP1, DIO2 TBX1)	Supraclavicular vs abdominal SC<br>↑ UCP1, DIO2, TBX1 mRNA, enhanced by NE treatment<br><br>Carotid perivascular and neck SC<br>Adipocyte vs progenitor<br>↑ UCP1 mRNA (neck SC)<br>FSK treatment<br>↑ UCP1 mRNA (carotid perivascular and neck SC)
Alessio et al, 2019<br>Italy [58]	BM stromal cells<br><br>Growth medium<br>MEM-alpha<br>10% FBS<br>100 U/mL penicillin<br>100 mg/mL streptomycin<br>1x L-Glutamine<br>3 ng/mL bFGF<br><br>Induction at 70-80% confluency	Adipocyte differentiation<br>**D1-D21**<br>DMEM (high glucose)<br>10% FBS<br>0.5 mM IBMX<br>1 mM dexa<br>10 µg/mL insulin<br>200 µM indomethacin	-	mRNA (UCP1)	Detectable UCP1 mRNA
Hedesan et al., 2019<br>Austria [59]	SVF from WAT (abdominal SC)<br><br>Growth medium<br>DMEM<br>10% FBS	Brown adipocytes<br>Growth medium +<br>**D1-D2**<br>0.5 mM IBMX<br>1 µM dexa	Non-isoproterenol stimulated cells	mRNA (UCP1, DIO2, PGC1α)	Brwon adipocytes vs non-isoproterenol stimulated cells<br>↑ UCP1, DIO2 mRNA<br>↔ PGC1α mRNA

	Induction at 100% confluency	850 nM insulin<br>5 µM Rosi<br>2 nM T3<br>**D2-D6**<br>850 nM insulin<br>5 µM Rosi<br>2 nM T3<br>± 10 µM isoproterenol<br>(6 h before harvesting)			
Jash et al., 2019<br>US [60]	SVF from WAT (abdominal SC)<br><br>Growth medium<br>MEM-alpha<br>10% FBS<br>100 U/mL penicillin<br>100 µg/mL streptomycin<br><br>Induction: 2 days post-confluency	Brite adipocytes<br>DMEM-F12<br>33 µM biotin<br>17 µM pantothenate<br>**D1-D7**<br>0.5 mM IBMX<br>0.1 µM dexa<br>100 nM insulin<br>1 µM Rosi<br>2 nM T3<br>10 µg/mL transferrin<br>**D7-D10**<br>0.1 µM dexa<br>100 nM insulin<br>**D10-D17**<br>1 µg/mL Rosi or 1 nM CL316,243	White adipocytes<br>DMEM-F12<br>33 µM biotin<br>17 µM pantothenate<br>**D1-D7**<br>0.5 mM IBMX<br>100 nM dexa<br>100 nM insulin<br>1 µM Rosi<br>2 nM T3<br>10 µg/mL transferrin<br>**D7-D10**<br>100 nM dexa<br>100 nM insulin<br>**D10-D17**<br>Medium only	mRNA (UCP1, PGC1α, PGC1β, PRDM16, C/EBPβ, CIDEA, DIO2, ELOVL3, P2RX5, CITED, CD137, TBX1, TMEM26, MTUS1, KCNK3, LHX8, ZIC1, PAT2, FBOX31, miR206, miR133β)<br><br>OCR (Seahorse®)	Brite vs white adipocytes (induction with Rosi)<br>↑ UCP1, PGC1α, PGC1β, PRDM16, CIDEA, DIO2, ELOVL33, CITED, CD137, TBX1, TMEM26, MTUS1, KCNK3<br>↔ C/EBPβ, P2RX5, LHX8, ZIC1, PAT2, FBOX31, miR206, miR133β<br><br>Brite vs white adipocytes (induction with CL316,243)<br>↑ OCR
Jespersen et al., 2019<br>Denmark [61]	SVF from adipose tissue (abdominal SC and perirenal)<br><br>Growth medium<br>DMEM-F12<br>10% FBS<br>1% penicillin/streptomycin<br>1 nM FGF1<br><br>Induction 2 d after confluency	Adipocyte differentiation<br>Growth medium without FBS and FGF1<br>+<br>10 µg/mL transferrin<br>**D1-D3**<br>0.54 mM IBMX<br>0.1 µM dexa<br>100 nM insulin<br>0.2 µM Rosi<br>2 nM T3<br>**D3-D6**<br>0.1 µM dexa<br>100 nM insulin<br>0.2 µM Rosi<br>2 nM T3<br>**D6-D12**<br>0.1 µM dexa<br>100 nM insulin<br>2 nM T3<br>+ 10 µM NE for 4 h before harvesting	SC vs perirenal<br><br>NE vs vehicle	mRNA (UCP1, PPGC1α)<br><br>Protein (UCP1)<br><br>OCR (Seahorse ®)	Perirenal vs abdominal SC<br>↑ basal UCP1, PPGC1α mRNA<br>↑ NE-stimulated UCP1, PPGC1α mRNA<br>↔ basal OCR<br>↑ NE-stimulated OCR<br>↑ UCP1 protein
Kim et al., 2019<br>South Korea [62]	ADSCs (Cell Engineering for Origin, Seoul, Korea)<br><br>Growth medium<br>DMEM	Brown adipocytes<br>Growth medium +<br>0.5 mM IBMX<br>0.5 µM dexa<br>20 nM insulin	Non-differentiated cells	mRNA (UCP1, PGC1α, PRDM16)<br><br>Protein (UCP1, PGC1α, CIDEA)	↑ UCP1, PGC1α, PRDM16 mRNA<br>↓ PRDM16 mRNA<br><br>↑ mitochondrial content

	10% FBS<br>125 μM indomethacin<br><br>Confluency at induction: NR	1 nM T3<br>Duration: NR		Mitochondrial content (MitoTracker®)	
Li et al., 2019<br>China, US [63]	SVF from WAT (abdominal SC)<br><br>Growth medium<br>DMEM-F12<br>10% FBS<br>100 IU/mL penicillin/streptomycin<br><br>Induction at 100% confluency	Beige adipocytes<br>Growth medium +<br>**D1-D14**<br>0.5 mM IBMX<br>0.25 μM dexa<br>10 μM insulin<br>**D14-D18**<br>+irisin 50 nM	White adipocytes<br>Growth medium +<br>**D1-D18**<br>0.5 mM IBMX<br>0.25 μM dexa<br>10 μM insulin	mRNA (UCP1, PGC1α, PRDM16)<br><br>Protein (UCP1)<br><br>OCR, ECAR (Seahorse®)	Beige vs white adipocytes<br>↑ UCP1, PGC1α, PRDM16 mRNA<br>↑ UCP1 protein<br>↑ OCR, ECAR
Li et al., 2019<br>China [64]	SVF from adipose tissue (fetal interscapular BAT)<br><br>Growth medium<br>DMEM<br>10% FBS<br>1% penicillin/streptomycin<br>20 ng/mL bFGF<br><br>Induction at 100% confluency	Differentiated brown adipocytes<br>**D1-D4**<br>DMEM/F12<br>0.5 mM IBMX<br>0.5 mM dexa<br>100 nM insulin<br>1 μM Rosi<br>1 nM T3<br>10 μg/mL transferrin<br>17 μM pantothenate<br>33 μM biotin<br>**D4-D6**<br>DMEM/F12<br>100 nM insulin<br>1 nM T3<br>FSK (3, 6, 12 h before harvesting, concentration NR)	Non-FSK stimulated cells	mRNA (UCP1, PGC1α)	FSK-stimulated vs non-FSK stimulated brown adipocytes<br>↑ UCP1, PGC1α mRNA
Min et al., 2019<br>Denmark, US [65]	Sprouting cells from cultured human adipose tissue (neck, abdominal SC)<br><br>Growth medium<br>EBM2<br>EGM2-MV<br>35% FBS<br><br>Induction at 100% confluency	Beige adipocytes<br>**D1-D3**<br>DMEM<br>10% FBS<br>0.5 mM IBMX<br>1 μM dexa<br>1 μg/mL insulin<br>**D3-D14**<br>DMEM<br>10% FBS<br>**D14-D17**<br>DMEM<br>10% FBS<br>10 μM FSK	Non-differentiated cells<br>DMEM<br>10% FBS	mRNA (UCP1, DIO2, CIDEC)	Beige vs non-differentiated cells<br>↑ UCP1, DIO2, CIDEC mRNA
Nagy et al., 2019<br>Hungary [66]	SVF from adipose tissue (pericardial/mediastinal)<br><br>Growth medium<br>DMEM-F12-Ham's<br>10% FBS<br><br>Induction at 100% confluency	Beige adipocytes<br>DMEM-F12-Ham's without FBS<br>**D1-D3**<br>0.5 mM IBMX<br>1 μM dexa<br>850 nM insulin<br>0.2 nM T3<br>10 μg/mL transferrin	White adipocytes<br>DMEM-F12-Ham's without FBS +<br>10 μg/mL of transferrin<br>**D1-D4**<br>0.5 mM IBMX<br>25 nM dexa<br>20 nM insulin<br>2 μM Rosi	No and size of lipid droplets<br><br>mRNA (UCP1, PRMD16, TMEM26, TBX1)<br><br>Protein (UCP1, TBX1)	Beige vs white adipocytes<br>↑ No of lipid droplets<br>↓ Lipid droplet size<br>↑ mitochondrial content<br>↔ Basal and cAMP-stimulated OCR<br>↑ UCP1, PRDM16 mRNA<br>↔ TMEM26, TBX1 mRNA<br>↑ UCP1 protein

		**D3-D9**<br>850 nM insulin<br>0.2 nM T3<br>0.1 or 0.5 µM Rosi<br>10 µg/mL transferrin<br>**D9-D16**<br>850 nM insulin<br>0.2 nM T3<br>0.02, 0.1 or 0.5 µM Rosi	0.2 nM T3<br>0.1 µM cortisol<br>**D4-D14**<br>20 nM insulin<br>0.2 nM T3<br>0.1 µM cortisol	OCR (basal and cAMP-stimulated, Seahorse®)	↔ TBX1 protein
Velickovic et al., 2019<br>UK, US [67]	BM-derived MSCs (Lonza)<br><br>Growth medium: NR<br><br>Confluency at induction: NR	Adipogenic differentiation<br>**D1-D21**<br>Growth medium<br>0.5 mM IBMX<br>1 µM dexa<br>10 µg/mL insulin<br>1 nM T3<br>1 µM Rosi<br>1 mM caffeine	-	Protein (UCP1)<br><br>Mitochondrial content (MitoTracker®)<br><br>OCR, ECAR (Seahorse®)	Detectable mitochondria Detectable UCP1 protein<br>Detectable OCR, ECAR
Raajendiran et al., 2019<br>Australia, US [68]	Adipose progenitor cells sorted from SVF from WAT (omental, abdominal SC, gluteofemoral SC; $CD34^-$, $CD34^{low}$, $CD34^{high}$)<br><br>Growth medium: NR<br><br>Induction at 100% confluency	DMEM-F12<br>1% penicillin/streptomycin<br>3.3 µM biotin<br>17 µM pantothenate<br>10 µg/mL transferrin<br>**D1-D3**<br>0.5 mM IBMX<br>0.25 µM dexa<br>20 nM insulin<br>2 µM Rosi<br>0.2 nM T3<br>**D3-D7**<br>0.5 mM IBMX<br>0.25 µM dexa<br>20 nM insulin<br>0.2 nM T3<br>**D7-D11**<br>0.25 µM dexa<br>20 nM insulin<br>0.2 nM T3<br>± 1 µM isoproterenol for 4 h before harvesting	$CD34^-$ cells vs $CD34^{low}$ and $CD34^{high}$ cells	mRNA (PPGC1α, CITED1, UCP1)<br><br>OCR (Seahorse®)	$CD34^-$ cells vs $CD34^{low}$ and $CD34^{high}$ cells<br>↑ UCP1 mRNA<br>↔ PPGC1α, CITED1 mRNA<br>↑ isoproterenol stimulated UCP1, PPGC1α, CITED1 mRNA<br>↑ isoproterenol stimulated OCR and proton leak
West et al, 2019<br>US [69]	Embryonic stem cells<br><br>Growth medium<br>NP110, NP88: Smooth Muscle Medium 2 with supplements (PromoCell)<br>C4ELS5.1, C4ELSR2: EpiLife Medium, low serum growth supplement (Life Technologies)<br>E3: DMEM (high glucose), 20% FBS<br>E85: DMEM (high glucose), 10% FBS	HiStem-C+<br>**Protocol 1**<br>**D1-D14:** BMP4, Rosi, T3, CL 316,243<br>**Protocol 2**<br>**D1-D14:** Rosi, T3, CL 316,243<br>**Protocol 3**	Differentiated vs non-differentiated cells<br><br>Different differentiation protocols<br><br>Differentiated NP88 cells vs differentiated control white and brown adipocytes	mRNA (UCP1, CITED), Illumina bead array gene expression<br><br>Protein (UCP1)<br><br>Mitochondrial content (MitoTracker®)<br><br>OCR (Seahorse®)	Differentiated vs non-differentiated cells<br>Protocol 1<br>↑ UCP1 mRNA (C4ELS5.1, C4ELS5.2, C4ELS5.5, NP88, NP110), ↔ UCP1 mRNA (E3)<br>↑ CITED mRNA (C4ELS5.1, C4ELS5.2, C4ELS5.5, NP88, NP110, E3)<br>↑ UCP1 protein (C4ELS5.1, NP88, NP110)<br>↑ mitochondrial content (all lines)

	All media supplemented with 2 mM glutamax, 1% penicillin / streptomycin<br><br>Induction at 100% confluency	**D1-D14:** Rosi, T3, CL 316,243; D13-D14: FGF<br>**Protocol 4**<br>**D1-D14:** Rosi, T3, CL 316,243; 28ºC for 4h before harvesting<br><br>For OCR (NP88 line)<br>D1-D4: Rosi, T3, insulin, transferrin, selenium in serum-free medium<br><br>BMP4: 10 ng/mL<br>Rosi: 1 µM<br>T3: 2 nM<br>FGF21: 50 ng/mL<br>CL 316,243: 10 µM<br>Insulin: 6.25 µg/mL<br>Transferrin: 6.25 µg/mL<br>Selenium: 6.25 ng/mL			Different differentiation protocols<br>↔ UCP1, CITED mRNA (all cell lines)<br><br>Differentiated NP88 cells vs differentiated control white and brown adipocytes<br>↑ OCR (basal, ATP-linked, maximal)<br>↑ ECAR
Wu et al., 2019<br>China [70]	Fetal brown preadipocytes (ZenBio)<br><br>Growth medium<br>DMEM<br>10% FBS<br>1% penicillin/streptomycin<br><br>Induction at 100% confluency	Growth medium +<br>**D1-D2**<br>0.5 mM IBMX<br>1 µM dexa<br>850 nM insulin<br>1 nM T3<br>125 nM indomethacin<br>Rosi (concentration NR)<br>**D2-D10**<br>850 nM insulin<br>1 nM T3<br>Rosi (concentration NR)	Growth medium +<br>**D1-D2**<br>0.5 mM IBMX<br>1 µM dexa<br>850 nM insulin<br>1 nM T3<br>125 nM indomethacin<br>**D2-D10**<br>850 nM insulin<br>1 nM T3	Protein ($UCP1^+$ cells)	Adipocytes differentiated in the presence of Rosi vs vehicle<br>↑ $UCP1^+$ cells
Ghandour et al, 2018<br>Germany [71]	MADSCs<br><br>Growth medium<br>DMEM-F12<br>10% FBS<br>15 mM HEPES<br>60 µg/mL penicillin<br>50 µg/mL streptomycin<br>2.5 ng/mL FGF2 (until confluency)<br><br>Induction 2 d after confluency	Beige adipocytes<br>DMEM-F12<br>10 µg/mL transferrin<br>**D1-D2**<br>0.5 mM IBMX<br>1 µM dexa<br>10 nM insulin<br>0.2 nM T3<br>**D2-D8**<br>10 nM insulin<br>0.2 nM T3<br>0.1 µM Rosi<br>**D8-D13**<br>10 nM insulin<br>0.2 nM T3<br>**D13-D17**<br>10 nM insulin<br>0.2 nM T3	White adipocytes<br>DMEM-F12<br>10 µg/mL transferrin<br>**D1-D2**<br>0.5 mM IBMX<br>1 µM dexa<br>10 nM insulin<br>0.2 nM T3<br>**D2-D8**<br>10 nM insulin<br>0.2 nM T3<br>100 nM Rosi<br>**D8-D13**<br>10 nM insulin<br>0.2 nM T3<br>**D13-D17**<br>10 nM insulin<br>0.2 nM T3	mRNA (UCP1, CIDEA)<br><br>OCR (Seahorse®)	Beige vs white adipocytes<br>↑ UCP1, CIDEA mRNA<br>↑ OCR (basal, uncoupled, maximal, spare capacity)

			100 nM Rosi		
Haynes et al., 2018<br>US [72]	Endothelial cells ($CD31^{+}/CD34^{+}$) from SVF from WAT (omental, SC)<br><br>Growth medium<br>EGM2<br><br>Confluency at induction: NR	Beige/brown adipocytes<br>**D1-D14**<br>DMEM-F12<br>5% FBS<br>50 µg/mL of penicillin/streptomycin<br>5 µg/mL insulin<br>1 µM Rosi	Non-differentiated cells<br>**D1-D14**<br>DMEM-F12<br>5% FBS<br>50 µg/mL of penicillin/streptomycin	mRNA (UCP1, CIDEA)	Beige/brown adipocytes vs non-differentiated cells<br>↑ UCP1, Cidea mRNA
Khanh et al., 2018<br>Japan [73]	SVF from adipose tissue (infants and elderly)<br><br>Growth medium<br>IMDM<br>10% FBS<br>2 mg/ml L-glutamine<br>100 IU/mL penicillin<br>5 ng/mL bFGF<br><br>Induction at 80% confluency	Beige adipocytes<br>**D1**<br>Growth medium without bFGF<br>**D2-D3**<br>Growth medium without bFGF +<br>IBMX (concentration NR)<br>Dexa (concentration NR)<br>2 µg/mL insulin<br>T3 (concentration NR)<br>Transferrin<br>**D3-D7/21**<br>2 µg/mL insulin<br>T3<br>Rosi	White adipocytes<br>Growth medium +<br>**D1-D20**<br>0.1 µM dexa<br>2 µg/mL insulin<br>0.1 mM indomethacin<br><br>Beige vs white adipocytes<br><br>Infant vs elderly beige adipocytes	mRNA (UCP1, PGC1α, CIDEA, COX8B, PRDM16)<br><br>Protein (UCP1)<br><br>Mitochondrial content (MitoTracker®)	Beige vs white adipocytes<br>↑ UCP1, PGC1α, CIDEA, COX8B, PRDM16 mRNA<br>↑ mitochondrial content<br><br>Infant vs elderly beige adipocytes<br>↑ UCP1, PGC1α, CIDEA, COX8B, PRDM16 mRNA<br>↑ UCP1 protein<br>↑ mitochondrial content
Loh et al., 2018<br>Australia [74]	SVF from WAT (subacromial)<br><br>Growth medium<br>DMEM<br>1% penicillin/streptomycin<br>15% FCS<br><br>Induction 2 d after confluency	Brown/beige adipocytes<br>**D1-D7**<br>DMEM<br>2% FCS<br>1% penicillin/streptomycin<br>0.5 mM IBMX<br>25 µM dexa<br>1 µM insulin<br>1 µM Rosi or 1 µM Pio<br>250 nM T3<br>**D7-D18**<br>DMEM<br>2% FCS<br>1% penicillin/streptomycin<br>1 µM insulin<br>1 µM Rosi or 1 µM Pio	White adipocytes<br>**D1-D7**<br>DMEM<br>2% FCS<br>1% penicillin/streptomycin<br>0.5 mM IBMX<br>25 µM dexa<br>1 µM insulin<br>250 nM T3<br>1 mM Rosi<br>**D7-D18**<br>DMEM<br>2% FCS<br>1% penicillin/streptomycin<br>1 µM insulin	Protein (UCP1)	Brown/beige vs white adipocytes<br>↑ UCP1 protein
Nascimento et al., 2018<br>Netherlands, US [75]	SVF from adipose tissue (DN and SC)<br><br>Growth medium: NR<br>4 ng/mL bFGF 2 d before confluency	Brown adipocytes (DN)<br>Growth medium +<br>33 µM biotin<br>17 µM pantothenate	White adipocytes (SC)<br>Growth medium +<br>33 µM biotin<br>17 µM pantothenate	OCR (Seahorse®)	Brown vs white adipocytes<br>↑ NE-stimulated OCR<br><br>Brown adipocytes

	Induction at 100% confluency	10 µg/mL transferrin<br>**D1-D7**<br>0.25 mM IBMX<br>0.1 µM dexa<br>100 nM insulin<br>5 µM Rosi<br>2 nM T3<br>4 ng/mL BMP4<br>**D7-D14**<br>0.25 mM IBMX<br>0.1 µM dexa<br>100 nM insulin<br>2 nM T3<br>1 µM NE (1 h before harvesting)	10 µg/mL transferrin<br>**D1-D7**<br>0.25 mM IBMX<br>100 nM dexa<br>100 nM insulin<br>5 µM Rosi<br>2 nM T3<br>**D7-D14**<br>0.25 mM IBMX<br>100 nM dexa<br>100 nM insulin<br>2 nM T3<br>1 µM NE (1 h before harvesting)	Correlation between OCR and UCP1 mRNA, OCR and ZIC1 mRNA	Positive correlation between OCR and UCP1 mRNA and ZIC1 mRNA
Pisani et al., 2018<br>France [76]	MADSCs<br><br>Growth medium<br>DMEM<br>10% FBS<br>15 mM HEPES<br>2.5 ng/mL FGF2<br><br>Induction 2 d after confluency	Brite adipocytes<br>DMEM-F12-Ham's<br>10 µg/mL transferrin<br>**D1-D2**<br>0.5 mM IBMX<br>1 µM dexamethasone<br>10 nM insulin<br>0.2 nM T3<br>**D2-D8**<br>10 nM insulin<br>0.1 µM Rosi<br>0.2 nM T3<br>**D8-D13**<br>10 nM insulin<br>0.2 nM T3<br>**D13-D18**<br>10 nM insulin<br>0.1 µM Rosi<br>0.2 nM T3	White adipocytes<br>DMEM-F12-Ham's<br>10 µg/mL transferrin<br>**D1-D2**<br>0.5 mM IBMX<br>1 µM dexa<br>10 nM insulin<br>0.2 nM T3<br>**D2-D8**<br>10 nM insulin<br>100 nM Rosi<br>0.2 nM T3<br>**D8-D18**<br>10 nM insulin<br>0.2 nM T3	mRNA (UCP1, CIDEA)<br><br>Protein (UCP1, mitochondrial complex I, II, III, IV, V)<br><br>Mitochondrial content (mtDNA)<br><br>OCR (Seahorse®)	Brite vs white adipocyte<br>↑ UCP1, CIDEA mRNA<br>↑ UCP1 protein<br>↑ mitochondrial complex II, III, V<br>↔ mitochondrial complex I, IV<br>↑ mitochondrial content<br>↑ OCR (basal, uncoupled, maximal)
Rashnonejad et al, 2018<br>Turkey [77]	Umbilical cord MSC, SVF from WAT (abdominal SC)<br><br>Growth medium<br>Umbilical cord MSC<br>IMDM<br>20% FBS<br>100 U/mL penicillin<br>100 µg/mL streptomycin<br>2 mM L-glutamine<br>10 ng/mL bFGF<br><br>SVF from adipose tissue<br>DMEM-F12<br>20% FBS<br>100 U/mL penicillin<br>100 µg/mL streptomycin<br>2 mM L-glutamine<br>10 ng/mL bFGF<br>5 ng/mL EGF	Brown adipocytes<br>DMEM (high glucose)<br>20% FBS<br>**D1**<br>100 ng/mL BMP7<br>**D2-D7**<br>0.5 mM IBMX<br>1 µM dexa<br>1 µM insulin<br>200 nM T3<br>200 µM indomethacin<br>10 µg/mL bovine transferrin<br>100 ng/mL BMP7<br>**D7-D28**<br>1 µM insulin<br>200 nM T3<br>1 µM Rosi<br>10 µg/mL bovine transferrin<br>100 ng/mL BMP7	White adipocytes<br>DMEM (high glucose)<br>20% FBS<br>**D1-D7**<br>0.5 mM IBMX<br>1 µM dexa<br>1 µM insulin<br>200 nM T3<br>0.2 mM indomethacin<br>10 µg/mL bovine transferrin<br>**D7-D28**<br>1 µM insulin<br>200 nM T3<br>10 µg/mL bovine transferrin<br>1% filtered-sterile olive oil<br><br>Differentiated vs non-differentiated cells	Protein (UCP1)	UCP1 protein<br>↑ brown adipocytes from SVF from adipose tissue > adipocytes from umbilical cord MSC > non-differentiated cells<br>↑ brown vs white adipocytes from umbilical cord MSC and SVF from adipose tissue

	Induction at 80% confluency		Cells differentiated from umbilical cord MSC vs adipose tissue		
Velickovic et al., 2018<br>UK [78]	ADSCs (ThermoFisher)<br><br>Growth medium<br>DMEM<br>10% FBS<br>1% penicillin/streptomycin<br>1% L-glutamine<br>1% non-essential amino acids<br><br>Induction at 80% confluence	Adipocytes<br>Growth medium +<br>**D1-D9**<br>0.1 mM IBMX<br>1 µM dexa<br>10 µg/mL insulin<br>1 µM Rosi	Cells differentiated at 37 vs 32ºC	Lipid droplet size<br><br>Protein (UCP1)	Cells differentiated at 32 vs 37°C<br>↑ UCP1 protein<br>↑ no of smaller lipid droplets
Wang et al, 2018<br>China [79]	SVF from fetal brown adipose tissue<br><br>Growth medium<br>DMEM-F12<br>10% FBS<br>1% penicillin/streptomycin<br><br>Confluency at induction: NR	Adipocyte differentiation<br>**Protocol 1**<br>DMEM-F12<br>**D1-D4**<br>0.5 mM IBMX<br>1 µM dexa<br>430 nM insulin<br>1 µM Rosi<br>1 nM T3<br>10 µg/mL transferrin<br>33 µM biotin<br>17 µM pantothenate<br>**D4-D10**<br>430 nM insulin<br>1 nM T3	Variations in the main protocol<br><br>Comparison with different protocols<br>**Protocol 2**<br>DMEM (high glucose)<br>10% FBS<br>**D1-D7**<br>0.25 mM IBMX<br>5 µM dexa<br>860 nM insulin<br>1 µM Rosi<br>1 nM T3<br>100 µM indomethacin<br>10 µg/mL transferrin<br>**D7-D21**<br>160 nM insulin<br><br>**Protocol 3**<br>DMEM-F12<br>10% FBS<br>**D1-D2**<br>0.2 mM IBMX<br>5 µM dexa<br>860 nM insulin<br>1 µM Rosi<br>1 nM T3<br>**D2-D21**<br>860 nM insulin<br>5 µM dexa<br>1 nM T3<br><br>**Protocol 4**<br>DMEM-F12<br>**D1-D7**<br>0.5 mM IBMX<br>1 µM dexa<br>860 nM insulin<br>1 µM Rosi<br>1 nM T3<br>10 µg/mL transferrin	mRNA (UCP1, PGC1α, CIDEA)	Variations in the main protocol<br>UCP1 mRNA: DMEM-F12 > DMEM (high glucose) = DMEM (low glucose)<br>PGC1α, CIDEA mRNA: DMEM-F12 = DMEM (high glucose) = DMEM (low glucose)<br><br>UCP1, PGC1α, CIDEA mRNA: FBS + T3 = T3 > FBS > no FBS and T3<br><br>UCP1 mRNA: 0.5 mM IMBX > 0.25 mM IBMX > no IBMX<br>PGC1α, CIDEA mRNA: 0.5 mM IMBX = 0.25 mM IBMX > no IBMX<br><br>UCP1, PGC1α, CIDEA mRNA: 5 µM dexa = 1 µM dexa > no dexa<br><br>UCP1, PGC1α mRNA: 125 µM indomethacin = 100 µM indomethacin < no indomethacin<br>CIDEA mRNA: 125 µM indomethacin < 100 µM indomethacin = no indomethacin<br><br>UCP1 mRNA: ↑ 172, 430, 860, 1720 nM insulin vs 66 nM or no insulin (↑↑ 430, 860, 1720 nM insulin)<br>PGC1α mRNA: ↑ 66, 172, 430, 860 nM insulin vs 1720 nM or no insulin (↑↑ 66, 172, 430 nM insulin)<br>CIDEA mRNA: ↑ 66, 172, 430, 860, 1720 nM insulin<br><br>UCP1, PGC1α mRNA: ↑ biotin + transferrin + pantothenate or either alone vs none<br>CIDEA mRNA: ↑ biotin + transferrin + pantothenate vs either alone or none<br><br>UCP1, PGC1α, CIDEA mRNA: ↑ induction for 4 and 6 days vs 2 days

			33 µM biotin<br>17 µM pantothenate<br>1 nM GH<br>1 nM IGF-1<br><br>**Protocol 5**<br>DMEM-F12<br>**D1-D3**<br>0.54 mM IBMX<br>0.1 µM dexa<br>100 nM insulin<br>0.2 µM Rosi<br>2 nM T3<br>10 µg/mL transferrin<br>**D3-D12**<br>0.1 µM dexa<br>100 nM insulin<br>0.2 µM Rosi<br>2 nM T3<br>10 µg/mL transferrin		UCP1 protein: induction for 6 days > 4 days > 2 days<br><br>Comparison of different protocols<br>UCP1 mRNA: Protocol 1 = 2,3,4 > 1<br>PGC1α, CIDEA mRNA: Protocol 1 = 4 > 3 = 2 = 1<br>UCP1 protein: 1 = 3 = 4 > 1,2<br>UCP1 dependent OCR: 1 = 3 = 4 > 1 = 2
Su et al, 2018<br>US [80]	Preadipocytes (SC, PromoCell and Lonza)<br><br>Growth medium<br>DMEM<br>10% FBS<br><br>Induction at 100% confluency	Beige adipocytes<br>Growth medium +<br>**D1-D3**<br>0.5 mM IBMX<br>5 µM dexa<br>170 nM insulin<br>1 µM Rosi<br>2 nM T3<br>125 µM indomethacin<br>50 µg/mL ascorbic acid<br>5 µM SB<br>**D3-D12**<br>170 nM insulin<br>1 µM Rosi<br>2 nM T3<br>50 µg/mL ascorbic acid<br>5 µM SB<br>± 1 µM CL316,243 for 4 h before harvesting	Cells treated vs non-treated with CL316,243 for 4 h before harvesting	mRNA (UCP1)<br><br>OCR (Seahorse®)	Cells treated vs non-treated with CL316,243 for 4 h before harvesting<br>↔ UCP1 mRNA<br>↔ OCR (basal, ATP-linked, proton-leak linked, maximal)
Zhang et al., 2018<br>China [81]	SVF from embryonic WAT (leg and back) and adult WAT (SC)<br><br>Growth medium<br>DMEM-F12-Ham's<br>20% FBS<br>1% penicillin-streptomycin<br><br>Induction at 100% confluency	Beige adipocytes<br>**D1-D6/7**<br>DMEM (high-glucose)<br>2% FBS<br>0.5 mM IBMX<br>0.1 µM dexa<br>0.5 µM insulin<br>2 nM T3<br>30 µM indomethacin<br>17 µM pantothenate<br>33 µM biotin<br>± 1 µM NE 4 h before harvesting	Non-differentiated embryonic cells (D0)<br><br>Embryonic vs adult differentiated cells	mRNA (UCP1, PGC1α, PGC1β, CD137, TBX1, TMEM26, ZIC1)<br><br>Protein (UCP1, PGC1α)<br><br>Mitochondrial content (mtDNA)<br><br>OCR (Seahorse®)	Beige adipocytes vs non-differentiated embryonic cells<br>↑ basal UCP1, PGC1α, PGC1β, CD137, TBX1 mRNA<br>↔ basal TMEM26 mRNA<br>↑ NE-stimulated UCP1, PGC1α mRNA<br>↑ basal UCP1, PGC1α protein<br><br>Embryonic vs adult cells<br>↑ UCP1, PGC1β, CD137, TBX1, PRDM15, TMEM26<br>↑ UCP1 protein<br>↔ PGC1α protein<br>↑ mitochondrial content<br>↑ OCR

Berry et al., 2017<br>US [82]	SVF from WAT (abdominal and hip SC, ZenBio)<br><br>Growth medium<br>DMEM<br>10% FBS<br><br>Induction at 100% confluency	Beige adipocytes<br>Growth medium +<br>0.5 mM IBMX<br>5 µM dexa<br>0.5 µg/mL insulin<br>1 µM Rosi<br>2 nM T3<br>Duration: NR	-	mRNA (UCP1, PGC1α)	Detectable UCP1, PGC1α mRNA
Cambria et al, 2017<br>Italy [83]	BM stromal cells<br><br>Growth medium<br>MEM-alpha with L-Glutamine and nucleosides<br>10% FBS<br>10,000 U/mL penicillin<br>10,000 µg/ml streptomycin<br>25 µg/mL amphotericin B<br>100 µM ascorbic acid<br><br>Induction at 100% confluency	Adipocyte differentiation<br>Three days before induction: 8.3 nM BMP7<br>Growth medium +<br>**D1-D4**<br>0.5 mM IBMX<br>5 mM dexa<br>20 nM insulin<br>1 nM T3<br>125 mM indomethacin<br>**D4-D7**<br>20 nM insulin<br>1 nM T3	Comparison of 2 and 4 differentiation cycles (D1-D7) vs non-differentiated cells	Protein (UCP1)	↑ UCP1 protein: 2 and 4 differentiation cycles vs non-differentiated cells
Jiang et al., 2017<br>China, US [84]	SVF from WAT (SC)<br><br>Growth medium<br>MesenPRO RS medium (Invitrogen)<br><br>Confluency at induction: NR	DMEM-F12<br>GlutaMAX<br>10% FBS<br>penicillin-streptomycin<br>33 µM biotin<br>17 µM pantothenate<br>**D1-D3**<br>0.5 mM IBMX<br>5 µM dexa<br>0.5 µg/mL insulin<br>5 µM Rosi<br>**D3-D10**<br>5 µM dexa<br>0.5 µg/mL insulin<br>5 µM Rosi	-	mRNA (UCP1, CIDEA)	Detectable UCP1, CIDEA mRNA
Kim et al, 2017<br>South Korea [85]	ADSCs (Cell Engineering for Origin)<br><br>Growth medium<br>DMEM<br>10% FBS<br>100 U/mL penicillin / streptomycin / gentamicin<br><br>Induction 2 d after confluency	Adipocyte differentiation<br>DMEM<br>10% FBS<br>**D1-D6**<br>0.5 mM IBMX<br>1 µM dexa<br>1 µg/mL insulin<br>100 µM indomethacin<br>**D6-D10**<br>1 µg/mL insulin	Differentiated vs non-differentiated cells	mRNA (UCP1, PGC1α, TMEM26, TBX1, CD137)<br><br>Protein (UCP1, PGC1α, TMEM26, TBX1, CD137)	↑ UCP1, TMEM26, TBX1, CD137 mRNA<br>↓ PGC1α mRNA<br>↑ TMEM26, TBX1, CD137 protein<br>↓ PGC1α protein<br>↔ UCP1 protein
Liczano et al., 2017<br>Colombia [86]	SVF from WAT (abdominal SC)<br><br>Growth medium<br>DMEM-F12	Adipocytes<br>DMEM-F12<br>10 µg/mL transferrin<br>**D1-D3**	Differentiated vs non-differentiated cells	mRNA (UCP1)<br><br>Protein (UCP1, PGC1α, CITED1)	Adipocytes vs non-differentiated cells<br>↑ UCP1 mRNA<br>↔ UCP1, PGC1α, CITED protein

	2.5% FBS<br>1 ng/mL bFGF<br>10 ng/mL EGF<br>8.7 µM insulin<br><br>Induction at 100% confluency	0.5 mM IBMX<br>0.1 µM dexa<br>66 nM insulin<br>1 µM Rosi<br>1 nM T3<br>**D3-D14**<br>66 nM insulin<br>1 nM T3<br>± 31ºC for 6 h before harvesting	Cells maintained at 31 vs 37ºC for 6 h before harvesting		Adipocytes at 31 vs 37ºC for 6 h<br>↑ PGC1α protein
Nyman et al., 2017<br>Sweden [87]	SVF from WAT (SC)<br><br>Growth medium<br>DMEM-F12-Ham's<br>10% FBS<br>50 U/mL penicillin<br>50 µg/mL streptomycin<br>10 mM HEPES<br>33 µM biotin<br>17 µM pantothenate<br>1 nM FGF<br><br>Induction at 90% confluency	Beige adipocytes<br>**D1-D32**<br>DMEM-F12<br>3% FCS<br>0.5 mM IBMX<br>0.1 µM dexa<br>850 nM insulin<br>5 nM T3<br>0.1 µM Rosi or 50 ng/mL BMP4<br>± isoproterenol (concentration and treatment duration NR)	White adipocytes<br>**D1-D32**<br>DMEM-F12<br>3% FCS<br>0.5 mM IBMX<br>100 nM dexa<br>0.85 µM insulin<br>5 nM T3	mRNA (UCP1, PGC1α, PRDM16, CITED1, TMEM26)<br><br>Protein (UCP1, PGC1α)<br><br>OCR (Seahorse®)	Beige vs white adipocytes (Rosi-induced)<br>↑ UCP1, PGC1α, CITED1, TMEM26, PRDM16 mRNA<br>↔ PRDM16 mRNA<br>↑ UCP1 protein<br>↔ PGC1α protein<br>↑ OCR (basal, spare capacity, maximal capacity)<br>↑ isoproterenol-stimulated OCR (proton leak, maximal capacity)<br><br>Beige vs white adipocytes (BMP4-induced)<br>↑ UCP1, PGC1α, CITED1, TMEM26, PRDM16 mRNA<br>↔ UCP1, PGC1α protein<br>↑ OCR (basal, spare capacity, maximal capacity)<br>↑ isoproterenol-stimulated OCR (proton leak, maximal capacity)
Pino et al., 2017<br>US [88]	SVF from adipose tissue (DN, neck SC, abdominal SC)<br><br>Growth media<br>MEM-alpha<br>10% FBS<br>100 IU/mL penicillin<br>100 µg/mL streptomycin<br><br>Induction at 100% confluency	DMEM-F12<br>**D1-D7**<br>0.5 mM IBMX<br>0.1 µM dexa<br>100 nM insulin<br>1 µM Rosi<br>2 nM T3<br>10 µg/mL transferrin<br>33 µM biotin<br>17 µM pantothenate<br>**D7-D11/13**<br>DMEM/F12<br>0.1 µM dexa<br>10 nM insulin<br>± 1 mM cAMP for 4 h before harvesting	Adipocytes from SVF from DN vs neck SC vs abdominal SC<br><br>Adipocytes from each depot vs SVF cells from each depot<br><br>Stimulated vs non-cAMP stimulated cells	mRNA (UCP1, PGC1α)<br><br>OCR (Seahorse®)	Adipocytes from SVF from DN vs neck SC vs abdominal SC<br>UCP1 mRNA: DN > neck SC > abdominal SC<br><br>Adipocytes vs SVF cells<br>Abdominal SC: ↑ UCP1 mRNA<br>Neck SC: ↑ UCP1 mRNA<br>DN: ↑↑ UCP1 mRNA<br><br>cAMP-stimulated adipocytes vs non-cAMP-stimulated adipocytes<br>Abdominal SC:<br>↔ UCP1 mRNA<br>↑ PGC1α mRNA<br>↔ basal and uncoupled OCR<br>Neck SC:<br>↔ UCP1 mRNA<br>↑ PGC1α mRNA<br>↔ basal and uncoupled OCR<br>DN: ↑ UCP1, PGC1α mRNA<br>↑ basal and uncoupled OCR

Rebello et al., 2017<br>Canada, US [89]	ADSCs (LaCell)<br><br>Growth medium<br>DMEM-F12<br>10% FBS<br>1% penicillin/streptomycin /amphotericin B<br><br>Induction 1 d after confluency	Brown adipocytes<br>DMEM/DMEM-F12<br>(70%/30%)<br>3% FBS<br>1%<br>penicillin/streptomycin/<br>amphotericin B<br>33 µM biotin<br>20 µM pantothenate<br>**D1-D4**<br>0.5 mM IBMX<br>1 µM dexa<br>100 nM insulin<br>5 µM Rosi<br>**D4-D10**<br>70% DMEM<br>30% DMEM/F12 1:1<br>100 nM insulin<br>0.1 µM Rosi	White adipocytes<br>DMEM/DMEM-F12<br>(70%/30%)<br>3% FBS<br>1%<br>penicillin/streptomycin/ampho<br>tericin B<br>33 µM biotin<br>20 µM pantothenate<br>**D1-D4**<br>0.5 mM IBMX<br>1µM dexa<br>0.1µM insulin<br>5 µM Rosi<br>**D4-D13**<br>70% DMEM<br>30% DMEM/F12 1:1<br>1 µM dexa<br>0.1µM insulin	mRNA (UCP1, PGC1α)<br>mRNA (UCP1)<br><br>OCR (Seahorse®)	Brown vs white adipocytes<br>↑ UCP1 mRNA<br>↔ OCR (basal, maximal, ATP-linked, reserve)
Yang et al., 2017<br>France, US [90]	ADSCs (abdominal SC)<br><br>Growth media<br>DMEM-F12<br>10% FBS<br>1% penicillin/streptomycin<br>1 ng/mL bFGF<br><br>Confluency at induction: NR	Brown adipocytes<br>DMEM (high glucose)<br>10% FBS<br>1%<br>penicillin/streptomycin<br>**D1-D28**<br>0.25 mM IBMX<br>0.5 µM dexa<br>850 nM bovine insulin<br>1 µM Rosi<br>120 nM T3<br>125 µM indomethacin	White adipocytes<br>DMEM (high glucose)<br>10% FBS<br>1% penicillin/streptomycin<br>**D1-D28**<br>0.5 mM IBMX<br>1 µM dexa<br>10 µg/mL bovine insulin<br>200 µM indomethacin	mRNA (UCP1, CIDEA)<br><br>Protein (UCP1)<br><br>OCR (Seahorse®)	Brown vs white adipocytes<br>Both ↑ UCP1, CIDEA mRNA from D0 to D28<br>↔ OCR (basal, proton leak, maximal respiration)
Abdul-Rahman et al, 2016<br>Hungary [91]	SVF from adipose tissue (pericardial)<br><br>Growth medium<br>DMEM-F12<br>10% FBS<br><br>Induction at 100% confluency	Beige adipocytes<br>DMEM-F12<br>10 µg/mL transferrin<br>**D1-D3**<br>0.5 mM IBMX<br>1 µM dexa<br>850 nM insulin<br>0.2 nM T3<br>**D3-D7**<br>850 nM insulin<br>0.2 nM T3<br>Rosi (concentration NR)	White adipocytes<br>DMEM-F12<br>10 µg/mL transferrin<br>**D1-D4**<br>0.5 mM IBMX<br>25 nM dexa<br>20 nM insulin<br>2 µM Rosi<br>0.2 nM T3<br>0.1 µM cortisol<br>**D4-D7**<br>20 nM insulin<br>0.2 nM T3<br>0.1 µM cortisol	mRNA (UCP1, CIDEA, TMEM26, PRDM16, TBX1)<br><br>OCR (Seahorse®)	Beige vs white adipocytes<br>↑ UCP1, CIDEA, TMEM26 mRNA<br>↔ PRDM16, TBX1 mRNA<br>↑ OCR (basal and cAMP-stimulated)
Barbagallo et al, 2016<br>Italy [92]	SVF from WAT (abdominal SC)<br><br>Growth medium<br>DMEM<br>10% FBS<br><br>Confluency at induction: NR	Adipocyte differentiation<br>**D1-D14**<br>DMEM-F12 (high glucose)<br>3% FBS<br>0.5 mM IBMX<br>0.1 µM dexa<br>100 nM insulin	Non-differentiated cells	mRNA (PGC1α, UCP1, UCP2, UCP3)<br><br>Protein (UCP1)	Differentiated vs non-differentiated cells<br>↔ PGC1α, UCP1, UCP2, UCP3 mRNA<br>Undetectable UCP1 protein

		60 μM indomethacin<br>10 μg/mL transferrin			
Barquissau et al, 2016<br>France [93]	MADSCs<br><br>Growth medium<br>DMEM-F12<br>10% FBS<br>15 mM HEPES<br>60 μg/mL penicillin<br>50 μg/mL streptomycin<br>2.5 ng/mL FGF2 (until confluency)<br><br>Induction 2 d after confluency	Beige adipocytes<br>DMEM-F12-Ham's<br>10 μg/mL transferrin<br>**D1-D3**<br>0.5 mM IBMX<br>1 μM dexa<br>10 nM insulin<br>0.2 nM T3<br>0.1 μM Rosi<br>**D3-D9**<br>10 nM insulin<br>0.2 nM T3<br>0.1 μM Rosi<br>**D9-D13**<br>10 nM insulin<br>0.2 nM T3<br>**D13-D17**<br>10 nM insulin<br>0.2 nM T3<br>0.1 μM Rosi	White adipocytes<br>DMEM-F12<br>10 μg/mL transferrin<br>**D1-D2**<br>0.5 mM IBMX<br>1 μM dexa<br>10 nM insulin<br>0.2 nM T3<br>100 nM Rosi<br>**D2-D8**<br>10 nM insulin<br>0.2 nM T3<br>100 nM Rosi<br>**D8-D17**<br>10 nM insulin<br>0.2 nM T3	mRNA (UCP1, PGC1α, ELOVL3, CPT1M, CITED1, TBX1, TMEM26, ZIC1)<br><br>Protein (UCP1)<br><br>Mitochondrial content (mtDNA)<br><br>OCR (Seahorse®)	Beige vs white adipocytes<br>↑ UCP1, PGC1α, ELOVL3, CPT1M, CITED1, TBX1, TMEM26 mRNA<br>↔ ZIC1 mRNA<br>↑ UCP1 protein<br>↑ mitochondrial content<br>↑ OCR
Fleckenstein-Elsen et al, 2016<br>Germany [94]	SVF from adipose tissue (abdominal SC and breast)<br><br>Growth medium<br>DMEM-F12<br>10% FCS<br>1 nM $NaHCO_3$<br>33 μM biotin<br>17 μM pantothenate<br><br>Confluency at induction: NR	Adipocyte differentiation<br>Growth medium with 2.5% FCS+<br>**D1-D7**<br>0.2 mM IMBX<br>1 μM dexa<br>100 nM insulin<br>0.25 μM troglitazone<br>**D7-D12**<br>1 μM dexa<br>100 nM insulin	Differentiated vs non-differentiated cells	mRNA (UCP1, CPT1M)	Differentiated vs non-differentiated cells<br>↔ UCP1, CPT1M mRNA
Giroud et al., 2016<br>France, Germany [95]	SVF from adipose tissue<br><br>Growth medium<br>DMEM<br>10% FBS<br>15 mM Hepes<br>2.5 ng/ml FGF2 (removed at 100% confluency)<br>60 μg/mL penicillin<br>50 μg/mL streptomycin<br><br>Induction 2 d after confluency	Brown/brite adipocytes<br>DMEM-F12-Ham's<br>10 μg/mL transferrin<br>**D1-D2**<br>0.5 mM IBMX<br>1 μM dexa<br>10 nM insulin<br>0.2 nM T3<br>**D2-D8**<br>10 nM insulin<br>0.2 nM T3<br>0.1 μM Rosi<br>**D8-D13**<br>10 nM insulin<br>0.2 nM T3<br>**D13-D18**<br>10 nM insulin<br>0.2 nM T3<br>0.1 μM Rosi	White adipocytes<br>DMEM-F12-Ham's<br>10 μg/mL transferrin<br>**D1-D2**<br>0.5 mM IBMX<br>1 μm dexa<br>10 nM insulin<br>0.2 nM T3<br>**D2-D8**<br>10 nM insulin<br>0.2 nM T3<br>100 nM Rosi<br>**D8-D13**<br>10 nM insulin<br>0.2 nM T3<br>**D13-D18**<br>10 nM insulin<br>0.2 nM T3	mRNA (UCP1)<br><br>Protein (UCP1)	Brown/brite vs white adipocytes<br>↑ UCP1 mRNA (D15, D16, D18)<br>↑ UCP1 protein (D18)

Klepac et al., 2016<br>Germany, Sweden, US [96]	MADSCs (provided by the laboratory of C. Dani, University of Nice Sophia, Antipolis)<br><br>Growth medium<br>DMEM (low glucose)<br>10% FBS<br>1x glutamine<br>10 mM Hepes<br>5000 IU/mL penicillin<br>5000 µg/mL streptomycin<br>2.5 ng/mL FGF2 (for 48 h after seeding)<br><br>Induction at 100% confluency	Brown/beige adipocytes<br>Growth medium without FGF2 +<br>10 µg/mL transferrin<br>**D1-D3**<br>0.1 mM IBMX<br>1 µM dexa<br>5 µg/mL insulin<br>0.2 nM T3<br>1 µM Rosi<br>**D3-D12**<br>5 µg/mL insulin<br>0.2 nM T3<br>1 µM Rosi	-	mRNA (UCP1)	Detectable UCP1 mRNA
Kristóf et al., 2016<br>Hungary [97]	SVF from WAT (abdominal SC)<br><br>Growth medium<br>DMEM-F12<br>10% FBS<br>100 U/mL penicillin/streptomycin<br>33 µM biotin<br>17 µM pantothenate<br><br>Induction at 100% confluency	Beige adipocyte<br>Growth medium without FBS +<br>10 µg/mL transferrin<br>**D1-D4**<br>0.5 mM IBMX<br>1 µM dexa<br>850 nM insulin<br>2 µM Rosi<br>0.2 nM T3<br>100 nM cortisol<br>**D4-D14**<br>20 nM human insulin<br>0.2 nM T3<br>100 nM cortisol<br>± 500 µM cAMP for 4 h before harvesting	White adipocyte<br>Growth medium without FBS +<br>10 µg/mL transferrin<br>**D1-D4**<br>0.5 mM IBMX<br>25 nM dexa<br>20 nM insulin<br>200 pM T3<br>**D4-D14**<br>20 nM insulin<br>500 nM Rosi<br>200 pM T3<br>± 500 µM cAMP for 4 h before harvesting	mRNA (UCP1, CIDEA, ELOVL3, PGC1α, TBX1, PRDM16, ZIC1)<br><br>Protein (UCP1)<br><br>Mitochondrial content (mtDNA)<br><br>OCR (Seahorse®)	Beige vs white adipocytes<br>↑ UCP1, CIDEA, ELOVL3, PGC1α, TBX1 mRNA<br>↔ ZIC1, PRDM16 mRNA<br>↑ UCP1 protein<br>↑ mitochondrial content<br>↑ non-stimulated and cAMP-stimulated basal respiration and proton leak
Lee et al, 2016<br>Australia, US [98]	SVF from adipose tissue (neck SC, perithyroid)<br><br>Growth medium<br>DMEM<br>10% NCS<br>10 mM HEPES<br>50 U/mL penicillin<br>50 µg/mL streptomycin<br><br>Confluency at induction: NR	Brown adipocytes (SVF from perithyroid adipose tissue)<br>Growth medium +<br>33 µM biotin<br>**D1-D2**<br>0.5 mM IBMX<br>1 µM dexa<br>850 nM insulin<br>1 µM Rosi<br>**D2-D12**<br>0.5 mM IBMX<br>1 µM dexa<br>850 nM insulin<br>1 nM T3<br>1 µM Rosi<br>1 nM CL 316,243<br>10 µg/mL transferrin	White adipocytes (SVF from neck SC adipose tissue)<br>Growth medium +<br>33 µM biotin<br>**D1-D2**<br>0.5 mM IBMX<br>1 µM dexa<br>850 nM insulin<br>1 µM Rosi<br>**D2-D12**<br>0.5 mM IBMX<br>1 µM dexa<br>850 nM insulin<br>1 nM T3<br>1 µM Rosi<br>1 nM CL 316,243<br>10 µg/mL transferrin	mRNA (UCP1, PGC1α, CIDEA, DIO2, PRDM16, TMEM26, CITED1, ZIC1, LHX8, HOXC8, HOXC9)<br><br>Protein (UCP1)	Brown vs white adipocytes<br>↑ UCP1, PGC1α, CIDEA, DIO2, PRDM16, TMEM26, CITED1, ZIC1, LHX8 mRNA<br>↔ HOXC8, HOXC9 mRNA<br>↑ UCP1 protein
Min et al, 2016<br>US [99]	Capillary network cells from adipose tissue explants (abdominal SC)<br><br>Growth medium	Adipocyte differentiation<br>**D1-D3**<br>DMEM	Cells stimulated vs non-stimulated with FSK	mRNA (UCP1, CIDEA, DIO2)<br><br>Protein (UCP1)	Differentiated cells stimulated vs non-stimulated with FSK<br>↑ UCP1, DIO2 mRNA (FSK for 6h, 3d, 7d, 14d)

	EGM2-MV or<br>DMEM + 10% FBS<br><br>Confluency at induction: NR	10% FBS<br>0.5 mM IBMX<br>1 µM dexa<br>1 µg/mL insulin<br>**D3-D10/24**<br>DMEM<br>10% FBS<br>± 50 µM FSK (D10-D24, D17-D24, D21-D24, 6h before harvesting at D24)<br>± 10 µM isoproterenol (6h before harvesting at D10)	Cells stimulated vs non-stimulated with isoproterenol<br><br>Differentiated adipocytes vs pericarotideal adipose tissue	OCR (Seahorse®)	↑ CIDEA mRNA (FSK for 3d, 7d, 14d)<br>↔ CIDEA mRNA (FSK for 6h)<br>↑ UCP1 protein (7d)<br>↔ UCP1 protein (3d)<br><br>Differentiated cells stimulated vs not non-stimulated with isoproterenol<br>↑ UCP1 mRNA<br>↔ UCP1 protein<br><br>Differentiated cells stimulated vs non-stimulated with FSK (7d)<br>↑ OCR (ATP-linked, proton linked, maximal capacity, reserve capacity)<br>↔ non-mitochondrial OCR<br><br>Differentiated adipocytes vs pericarotideal adipose tissue<br>↑ UCP1, DIO2 mRNA
Vargas et al, 2016<br>Colombia, Japan [100]	ADSCs<br><br>Growth medium<br>DMEM-F12<br>2.5% FBS<br>1 ng/mL bFGF<br>10 ng/mL EGF<br>8.7 µM insulin<br><br>Confluency at induction: NR	Adipocyte differentiation<br>DMEM-F12<br>10 µg/mL transferrin<br>**D1-D3**<br>0.5 mM IBMX<br>1 µM dexa<br>66 nM insulin<br>2 µM Rosi<br>1 nM T3<br>**D3-D13**<br>66 nM insulin<br>1 nM T3	Differentiated vs non-differentiated cells	Protein (UCP1, PGC1α, CITED, TFAM)	Differentiated vs non-differentiated cells<br>↔ UCP1, PGC1α, CITED, TFAM protein in differentiated cells (at D3 and D6)
Wang et al., 2016<br>China [101]	BM MSCs<br><br>Growth medium<br>MEM-alpha<br>10% FBS<br>100 U/mL penicillin<br>10 µg/mL streptomycin<br>0.25 µg/mL amphotericin B<br><br>Induction at 100% confluency	Brown adipocyte<br>**D1-D14/21**<br>MEM-alpha<br>10% FBS<br>0.1 µM dexa<br>10 µg/mL insulin<br>1 nM T3<br>50 µg/mL indomethacin<br>50 µg/mL ascorbate-2 phosphate<br><br>Brown adipocytes ± PKA activators<br>**D1-D21**<br>MEM-alpha<br>100 nM dexa<br>10 µg/mL insulin<br>1 nM T3<br>50 µg/mL indomethacin<br>50 µg/mL ascorbate-2 phosphate	Brown adipocytes vs non-differentiated MSC<br><br>Presence vs absence of PKA activators (IMBX or FSK)	mRNA (UCP1, TBX1, CD137, TMEM26)<br><br>Protein (UCP1, CD40, CD137, TBX1)	Brown adipocytes vs non-differentiated MSC<br>↑ UCP1 mRNA (D14, D21)<br>↑ TBX1, CD137 mRNA (D14, D21)<br>↔ TMEM 26 mRNA (D14, D21)<br>↑ UCP1, CD40, CD137, TBX1 protein (D14, D21)<br><br>Presence vs absence of PKA activators<br>↑ UCP1 mRNA<br>↓ CD40 mRNA<br>↓ CD137 mRNA (IBMX)<br>↑ UCP1 protein<br>↓ CD40, TBX1 protein

		± 0.45 mM IBMX or<br>10 μM FSK			
Wu et al., 2016<br>China [102]	SVF from adipose tissue (embryonic BAT, embryonic perirenal WAT, adult SC)<br><br>Growth medium<br>DMEM (high glucose)<br>20% FBS<br>1% penicillin/streptomycin<br><br>Induction at 100% confluency	Adipocyte differentiation<br>**D1-D7**<br>DMEM (high glucose)<br>2% FBS<br>0.5 mM IBMX<br>0.1 μM dexa<br>0.5 μM insulin<br>2 nM T3<br>30 μM indomethacin<br>17 μM pantothenate<br>33 μM biotin<br>± 1 μM NE for 4 h before harvesting	Adipocytes vs SVF (non-differentiated cells)<br><br>Adipocytes differentiated from SVF from different depots (embryonic BAT, embryonic perirenal WAT, adult SC)<br><br>Adipocytes stimulated with NE vs non-stimulated	mRNA (UCP1, PRDM16, PGC1α, ZIC1, PGC1β, CD137, TBX1, TMEM26)<br><br>Protein (UCP1)<br><br>Mitochondrial content (TFAM and NRF1 mRNA expression, mitochondrial copy number)<br><br>OCR (Seahorse®)	Perirenal differentiated adipocytes vs SVF<br>↑ UCP1, PRCM16, PGC1α mRNA<br>↑ UCP1 protein<br><br>Adipocytes differentiated from SVF from different depots<br>UCP1, PRDM16, PGC1α mRNA: embryonic BAT and embryonic perirenal > adult SC<br>ZIC1, PGC1β mRNA: embryonic BAT > embryonic perirenal and adult SC<br>CD137, TBX1, TMEM26 mRNA: embryonic perirenal > embryonic BAT > adult SC<br>UCP1 protein: embryonic BAT and embryonic perirenal > adult SC<br>Mitochondrial content: embryonic BAT and embryonic perirenal > adult SC<br><br>NE-stimulated vs non-stimulated adipocytes<br>Embryonic BAT: ↑ UCP1, PGC1α mRNA<br>Perirenal WAT: ↑ UCP1, PGC1α mRNA<br>Adult SC: ↔ UCP1, PGC1α mRNA
Xu et al, 2016<br>China [103]	Stromal cells from BM<br><br>Growth medium<br>MEM-alpha<br>10% FBS<br>1% penicillin / streptomycin<br><br>Induction at 100% confluency	Adipocyte differentiation<br>MEM-alpha<br>10% FBS<br>**D1-D14**<br>0.5 mM IBMX<br>1 μM dexa<br>10 μg/mL insulin<br>100 μg/mL indomethacin	Differentiated vs non-differentiated cells	mRNA (microarray and gene ontology characterization)	Differentiated cells: upregulation of genes involved in brown fat cell differentiation
Barclay et al., 2015<br>Australia [104]	SVF from adipose tissue (supraclavicular, abdominal SC)<br><br>Growth medium<br>DMEM<br>10% NCS<br>10 mM HEPES<br>1 nM T3<br>100 μM isoprenaline<br>850 nM insulin<br><br>Induction at 100% confluency	Adipocyte differentiation<br>**D1-D12**<br>DMEM<br>10% NCS<br>10 mM HEPES<br>0.5 mM IBMX<br>1 or 10 μM dexa<br>850 nM insulin<br>1 nM T3<br>1 μM Rosi<br>50 IU/mL penicillin<br>50 μg/mL streptomycin<br>10 μg/mL transferrin<br>33 μM biotin<br>17 μM pantothenate<br>1 nM human hGH<br>1 nM IGF-I	Adipocytes differentiated from SVF from supraclavicular vs abdominal SC adipose tissue<br><br>10 vs 1 μM dexa<br><br>Isoprenaline stimulation in the presence of 1 μM dexa vs no dexa	mRNA (UCP1, CIDEA, PGC1α)<br><br>OCR (Seahorse®)	Adipocytes differentiated from SVF from supraclavicular vs abdominal SC adipose tissue<br>↑ UCP1 and CIDEA mRNA at D6, D9, D12 in supraclavicular but not in abdominal SC<br>↑ PGC1α mRNA at D9, D12 in supraclavicular but not abdominal SC<br><br>Adipocytes differentiated in the presence of 10 vs 1 μM dexa<br>↑ UCP1, PGC1α mRNA in supraclavicular but not abdominal SC<br>↑ CIDEA mRNA in supraclavicular and abdominal SC<br>↑ OCR (uncoupled, maximal) in supraclavicular and abdominal SC<br><br>Isoprenaline stimulation in the presence of 1 μM dexa vs no dexa

		± 100 nM isoprenaline at D9 for 5 h before harvesting			↓ isoprenaline-induced increase in UCP1 mRNA<br>↓ isoprenaline-induced increase in OCR (uncoupled, maximal)
Gustafson et al., 2015<br>Sweden [105]	Preadipocytes (SC)<br><br>Growth medium<br>DMEM-F12-Ham's<br>10% FBS<br>2 mM glutamine<br>100 IU/mL penicillin<br>100 μg/mL streptavidin<br><br>Confluency at induction: NR	Adipocyte differentiation<br>**D1-D3**<br>DMEM-F12<br>3% FBS<br>2 mM glutamine<br>100 IU/mL penicillin<br>100 μg/mL streptavidin<br>0.5 mM IBMX<br>100 μM dexa<br>850 nM insulin<br>33 μM biotin<br>17 μM pantothenate<br>± 10 μM Pio or 100 ng/mL BMP4<br>**D3-D9**<br>Growth medium +<br>100 μM dexa<br>850 nM insulin<br>± 10 μM Pio or 100 ng/mL BMP4	Induction in the presence vs absence of Pio<br><br>Induction in the presence vs absence of BMP4	mRNA (TMEM26, ZIC1, UCP1, PGC1α)<br><br>Protein (UCP1)<br><br>Mitochondrial content (MitoTracker®)	Induction in the presence vs absence of Pio<br>↔ ZIC1, TMEM26, PGC1α mRNA<br><br>Induction in the presence vs absence of BMP4<br>↑ ZIC1 mRNA<br>↔ TMEM26, PGC1α mRNA<br>↑ UCP1 protein<br>↑ Mitochondrial content
Hartig et al, 2015<br>US [106]	Primary preadipocytes (abdominal SC)<br><br>Growth medium<br>DMEM-F12<br>10% FBS<br>100 U/mL penicillin<br>100 μg/mL streptomycin<br><br>Induction at 100% confluency	Adipocyte differentiation<br>Growth medium +<br>**D1-D4**<br>0.25 mM IBMX<br>0.5 μM dexa<br>100 nM insulin<br>3 μM Rosi	Non-differentiated cells	mRNA (UCP1, CITED1, CIDEA)<br><br>Protein (UCP1)	Differentiated vs non-differentiated cells<br>↑ UCP1, CITED1, CIDEA mRNA<br>↑ UCP1 protein
Kouidhi et al, 2015<br>France [107]	SVF from WAT (chin and knee)<br><br>Growth medium<br>DMEM<br>10% FBS<br>2.5 ng/mL FGF2<br><br>Induction at 100% confluency	Adipocyte differentiation<br>**D1-D10**<br>Growth medium +<br>0.5 mM IMBX<br>0.25 μM dexa<br>340 nM insulin<br>0.2 nM T3<br>1 μM Rosi<br>± 10 μM FSK for 4 h before harvesting	Adipocytes differentiated from chin vs knee adipose tissue SVF	mRNA (UCP1)<br><br>Protein (UCP1)	Adipocytes differentiated from chin vs knee adipose tissue SVF<br>↑ UCP1 mRNA<br>↑ UCP1 protein (basal and FSK-stimulated)
Loft et al., 2015<br>Denmark, France, UK, US [108]	MADSCs (SC WAT)<br><br>Growth medium<br>DMEM (low glucose)<br>10% FBS<br>10 mM HEPES<br>2 mM L-glutamine<br>62.5 μg/mL penicillin	Brite adipocytes<br>DMEM-F12-Ham's<br>10 μg/mL transferrin<br>**D1-D2**<br>0.5 mM IBMX<br>1 μM dexa<br>850 nM insulin<br>0.2 nM T3	White adipocytes<br>DMEM-F12-Ham's<br>10 μg/mL transferrin<br>**D1-D2**<br>0.5 mM IBMX<br>1 μM dexa<br>0.85 μM insulin<br>0.2 nM T3	mRNA (UCP1, CIDEA, DIO2, ELOVL3)<br><br>Protein (UCP1)<br><br>OCR (Searose®)	Brite vs white adipocytes<br>↑ UCP1, CIDEA, DIO2, ELOVL3 (D16, D19)<br>↑ UCP1 protein (D19)<br>↑ OCR (basal, uncoupled, maximal)<br><br>Brite adipocytes: 31 vs 37ºC for 4 h before harvesting (D19)

	100 µg/mL streptomycin<br>2.5 ng/mL FGF2<br><br>Induction 2 d after confluency	**D2-D9**<br>850 nM insulin<br>0.2 nM T3<br>0.5 µM Rosi<br>**D9-D12**<br>850 nM insulin<br>0.2 nM T3<br>**D12-D16**<br>850 nM insulin<br>0.2 nM T3<br>0.5 µM Rosi<br>**D16-D23**<br>850 nM insulin<br>0.2 nM T3<br>± 31 vs 37ºC for 4 h before harvesting	**D2-D9**<br>0.85 µM insulin<br>0.2 nM T3<br>0.5 µM Rosi<br>**D9-D23**<br>0.85 µM insulin<br>0.2 nM T3		↑ UCP1, ELOVL3, PGC1α mRNA
Okla et al, 2015<br>US [109]	SVF from adipose tissue (abdominal SC)<br><br>Growth medium<br>DMEM-F12-Ham's<br>10% FCS<br>15 mM HEPES<br>50 U/mL penicillin<br>50 µg/mL streptomycin<br><br>Confluency at induction: NR	Adipocyte differentiation<br>3 days before induction: growth medium ± 100 ng/mL BMP7<br>**D1-D3**<br>0.25 mM IBMX<br>1 mM dexa<br>0.1 mM insulin<br>0.2 nM T3<br>1 µM Rosi<br>0.125 µM indomethacin<br>± 100 ng/mL BMP7<br>**D3-D10:** adipocyte maintenance medium without Rosi or with 1 or 5 µM Rosi, NR	BMP7 treatment vs no BMP7 treatment	mRNA (UCP1, DIO2, CIDEA, ADRB3, CD137, TMEM26, PRDM16)<br><br>OCR (MitoXpress, Cayman Chemical®)	BMP7 treatment vs no BMP7 treatment (no Rosi D3-D7)<br>↑ UCP1, DIO2, CIDEA, ADRB3, CD137, TMEM26 mRNA<br>↔ PRDM16 mRNA<br><br>BMP7 treatment vs no BMP7 treatment (± Rosi D3-D7)<br>↑ UCP1 mRNA (no Rosi, 1 µM Rosi, 5 µM Rosi D3-D7)<br>↑ OCR (basal and cAMP stimulated)
Seiler et al, 2015<br>Singapore [110]	SVF from adipose tissue (fetal BAT, adult SC and omental adipose tissue)<br><br>Growth medium<br>DMEM-F12-Ham's<br>10% FCS<br>HEPES<br>1:100 penicillin/streptomycin<br>1 µg/mL anphotericin B<br><br>Induction at 100% confluency	Adipocyte differentiation<br>DMEM<br>10% FBS<br>1:100 penicillin/streptomycin<br>1 µg/mL anphotericin B<br>**D1-D7**<br>0.25 mM IMBX<br>0.5 µM dexa<br>850 nM insulin<br>1 µM Rosi<br>100 µM indomethacin<br>**D7-D21**<br>160 nM insulin<br>± 1 µM NE for 3 h before harvesting	Adipocytes differentiated from SVF of fetal brown adipose tissue vs adult SC and omental adipose tissue<br><br>Adipocytes differentiated from SVF of fetal brown adipose tissue and stimulated vs non-stimulated with NE	mRNA (UCP1, PGC1α, LHX8, ZIC1, CIDEA)<br><br>Protein (UCP1)<br><br>Mitochondrial content (MitoTracker)<br><br>OCR (Seahorse®)	Adipocytes differentiated from SVF of fetal brown adipose tissue vs adult SC and omental adipose tissue<br>↑ UCP1, PGC1α, LHX8, ZIC1 mRNA<br>↑ UCP1 protein<br>↑ PGC1α protein (fetal brown > adult SC > adult omental)<br>↑ mitochondrial content<br>↑ OCR (proton leak-linked, maximal)<br>↔ OCR (basal, ATP-linked)<br><br>Adipocytes differentiated from SVF of fetal brown adipose tissue and stimulated vs non-stimulated with NE<br>↑ UCP1, PGC1α mRNA
van den Beukel et al, 2015<br>Netherlands [111]	SVF from adipose tissue (abdominal SC and perirenal)	Adipocyte differentiation<br>Growth medium +	Adipocytes differentiated from SVF from perirenal vs abdominal SC adipose tissue	mRNA (UCP1)<br><br>Protein (UCP1)	Adipocytes differentiated from SVF from abdominal SC and perirenal adipose tissue<br>↑ UCP1 mRNA in women

	Growth medium<br>MEM-alpha<br>15% FCS<br>4 mM glutamine<br>100 U/mL penicillin<br>100 µg/mL streptomycin<br>0.25 µg/mL anphotericin B<br><br>Induction at 95-100% confluency	**D1-D15**<br>0.5 mM IBMX<br>0.01 µM dexa<br>60 µM indomethacin	Adipocytes differentiated from women vs men	OCR (Seahorse®)	↔ UCP1 mRNA in men<br><br>Adipocytes differentiated from women vs men<br>↔ UCP1 mRNA (perirenal)<br>↔ OCR, basal and uncoupled (perirenal)
Vargas et al., 2015<br>Colombia [112]	SVF from WAT (abdominal SC)<br><br>Growth medium<br>First 24 h<br>DMEM-F12<br>10% FBS<br>gentamicin 50 µg/mL<br>Thereafter<br>DMEM-F12<br>2.5% FBS<br>1 ng/mL bFGF<br>10 ng/mL EGF<br>Insulin 8.7 µM<br><br>Induction at 100% confluency	Adipocytes<br>DMEM-F12 +<br>10 µg/mL transferrin<br>**D1-D3**<br>0.5 mM IBMX<br>0.1 µM dexa<br>66 nM insulin<br>1 nM T3<br>± 1 µM Rosi<br>**D3-D14**<br>66 nM insulin<br>1 nM T3<br>± 31ºC for 4 h before harvesting	Adipocytes differentiated in the presence vs absence of Rosi<br><br>Adipocytes exposed to 31 vs 37ºC for 4 h before harvesting	Protein (UCP1, PGC1α, CITED1)	Adipocytes differentiated in the presence vs absence of Rosi<br>37ºC: ↑ UCP1, PGC1α protein, ↔ CITED protein<br>31ºC: ↑ UCP1, PGC1α, CITED protein
Xue et al, 2015<br>US [113]	SVF from adipose tissue (WAT: subcutaneous and subplatysmal neck fat depots / BAT: fat depots from carotid sheath, longus colli muscle, prevertebral region)<br><br>Growth medium<br>DMEM (high glucose)<br>10% FBS<br>1% penicillin / streptomycin<br><br>Induction after cells were grown to confluence for 6 d	Adipocyte differentiation<br>**D1-D12**<br>DMEM (high glucose)<br>2% FBS<br>0.5 mM IMBX<br>0.1 µM dexa<br>500 nM insulin<br>2 nM T3<br>30 µM indomethacin<br>33 µM biotin<br>17 µM pantothenate	Adipocytes diffentiated from SVF from brown vs white adipose tissue	mRNA (UCP1, PGC1α)	Adipocytes diffentiated from SVF from brown vs white adipose tissue<br>↑ UCP1, PGC1α mRNA
Zhu et al., 2015<br>China, US [114]	SVF from WAT (SC)<br><br>Growth medium: NR<br><br>Confluency at induction: NR	Adipocytes<br>DMEM-F12<br>10% FBS<br>33 nM biotin<br>4 µg/mL pantothenate<br>10 µg/mL transferrin<br>**D1-D3**<br>0.5 mM IBMX<br>0.1 µM dexa<br>70 nM insulin<br>1 µM Rosi<br>2 µM T3<br>**D3-D11**<br>0.1 µM dexa<br>70 nM insulin<br>2 µM T3	Adipose tissue from DN/perithyroid region	mRNA (UCP1, PRDM16, CIDEA, ELOVL6)	Differentiated adipocytes vs adipose tissue from DN/perithyroid region<br>↓ UCP1, PRDM16, CIDEA, ELOVL6 mRNA

Di Franco et al., 2014<br>Italy [115]	SVF from adipose tissue (periadrenal, abdominal SC)<br><br>Growth medium: NR<br><br>Confluency at induction: NR	Brown adipogenic differentiation (protocol 1)<br>**D1-D14**<br>DMEM<br>10% FBS<br>0.5 mM IBMX<br>1 µM dexa<br>10 µM insulin<br>250 nM T3 or 10 µM NE for the last 3 days<br><br>Brown adipogenic differentiation (protocol 2)<br>**D1-D14**<br>DMEM<br>10% FBS<br>0.5 mM IBMX<br>1 µM dexa<br>10 µM insulin<br>10 µM Rosi<br>250 nM T3 or 10 µM NE for the last 3 days	SVF from periadrenal vs abdominal white adipose tissue	mRNA (UCP1, PRDM16)	SVF from periadrenal vs abdominal white adipose tissue<br>White adipogenic differentiation: ↔ UCP1, PRDM16 mRNA<br>Brown adipogenic differentiation 1: ↔ UCP1, PRDM16 mRNA<br>Brown adipogenic differentiation 2: ↔ UCP1 mRNA, ↑ PRDM16 mRNA
Elsen et al, 2014<br>Germany [116]	SVF from adipose tissue (abdominal SC and breast)<br><br>Growth medium<br>DMEM-F12<br>10% FCS<br>14 nM $NaHCO_3$<br>33 µM biotin<br>17 µM panthotenic acid<br><br>Induction at 100% confluency	Adipocyte differentiation<br>Growth medium +<br>10 mg/mL transferrin<br>50 mg/mL gentamycin<br>**D1-D3**<br>66 nM insulin<br>5 µM troglitazone<br>1 nM T3<br>100 nM cortisol<br>**D3-D14**<br>66 nM insulin<br>1 nM T3<br>100 nM cortisol<br>± 50 ng/mL BMP4 or BMP7 (D1-D14)	Differentiated vs non-differentiated cells<br><br>BMP-treated vs non-treated adipocytes	mRNA (UCP1, ZIC1, PGC1α, PGC1β, UCP2, CYCS, PRDM16, PPARα, NRF1, TFAM, OXR1, UCP3, VDAC1)<br><br>Protein (mitochondrial complex proteins)<br><br>Mitochondrial content (CTCO2 protein)<br><br>OCR (Oroboros®)	Differentiated vs non-differentiated cells<br>↑ UCP1 mRNA<br>↔ ZIC1, PGC1α mRNA<br>↑ complex I, III, IV, V protein<br>↔ complex II protein<br><br>BMP-treated vs non-treated adipocytes<br>↑ PGC1β, UCP2, CYCS<br>↔ PRDM16, PPARα, NRF1, TFAM<br>↓ OXR1, UCP3, VDAC1<br>↑ complex I, II, III, IV, V protein<br>↑ mitochondrial content
Karbiener et al., 2014<br>Austria, France, Germany, Italy [117]	MADSCs<br><br>Growth medium<br>DMEM (low glucose)<br>10% FBS<br>10 mM HEPES<br>2 mM L-Glutamine<br>100 µg/ml Normocin<br>2.5 ng/ml FGF2<br><br>Induction 2 d after confluency	Brite adipocytes<br>DMEM-F12-Ham's<br>5 mM HEPES<br>2 mM L-glutamine<br>100 µg/mL normocin<br>10 µg/mL transferrin<br>**D1-D3**<br>0.1 mM IBMX<br>1 µM dexa<br>860 nM insulin<br>0.1 µM Rosi<br>0.2 nM T3<br>**D3-D16**<br>860 nM insulin<br>0.1 µM Rosi	Brite adipocytes<br>DMEM-F12-Ham's<br>5 mM HEPES<br>2 mM L-glutamin<br>100 µg/mL normocin<br>10 µg/mL transferrin<br>**D1-D3**<br>0.1 mM IBMX<br>1 µM dexa<br>860 nM insulin<br>100 nM Rosi<br>0.2 nM T3<br>**D3-D9**<br>860 nM insulin<br>100 nM Rosi	mRNA (UCP1, PGC1α, PRDM16, ADRB1)<br><br>Protein (UCP1)	Brite vs white adipocyte<br>↑ UCP1 mRNA<br>↔ PGC1α, PRDM16, ADRB1 mRNA<br>↑ UCP1 protein

		0.2 nM T3	0.2 nM T3<br>**D10-D16**<br>860 nM insulin<br>0.2 nM T3		
Kern et al., 2014<br>US [118]	SVF from WAT (abdominal and thigh, SC)<br><br>Growth medium: NR<br><br>Induction 2 d after confluency	Adipocyte differentiation<br>DMEM-F10-Ham's<br>3% FBS<br>15 mM Hepes<br>33 µM biotin<br>17 µM pantothenate<br>**D1-D3**<br>0.25 mM IBMX<br>1 µM dexa<br>100 nM insulin<br>1 µM Rosi<br>**D3-D10**<br>1 µM dexa<br>1 µM Rosi<br>16ºC for 30 min followed by 4 h at 37ºC vs maintenance at 37ºC before harvesting	Adipocytes exposed vs non-exposed to cold	mRNA (UCP1, PGC1α)	Adipocytes exposed vs non-exposed to cold<br>↑ UCP1, PGC1α mRNA
Silva et al, 2014<br>US [119]	Mediastinal ADSCs from tissue explants, derivation of clonal lines by limiting dilution (clone BADSC18 used due to high UCP1 expression)<br><br>Growth medium<br>DMEM (low glucose)<br>10% XcytePlus (iBiologics)<br>1x Glutamax<br>1x MEM-nonessential aminoacids<br><br>Induction 90% confluency	Brown adipocytes<br>**D1-D7/14/21/28**<br>DMEM (low glucose)<br>10% FBS<br>0.5 mM IBMX<br>5 µM dexa<br>0.5 µg/mL insulin<br>1 nM T3<br>1 µM Rosi<br>20 nM FNDC5	White adipocytes<br>**D1-D7/14/21/28**<br>DMEM (low glucose)<br>10% FBS<br>0.5 mM IBMX<br>5 µM dexa<br>0.5 µg/mL insulin<br>1 nM T3<br>1 µM Rosi	mRNA (UCP1, DIO2, PRDM16)<br><br>Protein (UCP1)<br><br>OCR (Seahorse®)	Brown vs white adipocytes<br>↑ DIO2, UCP1, PRDM16 mRNA<br><br>Brown adipocytes vs non-differentiated cells<br>↑ DIO2, UCP1, PRDM16 mRNA<br>↑ UCP1 protein<br>↑ OCR (D7, D14, D21, D28)
Murholm et al., 2013<br>Denmark [120]	MADSCs (provided by the laboratory of C. Dani, University of Nice Sophia, Antipolis)<br><br>Growth medium<br>DMEM (low glucose)<br>10% FBS<br>2 mM L-glutamine<br>10 mM HEPES<br>50 IU/mL penicillin<br>50 µg/mL streptomycin<br>2.5 ng/mL FGF2<br>Induction 2 d after confluency<br><br>Primary white SC preadipocytes (Lonza)<br>Growth medium: PBM-2<br><br>Induction 2 d after confluency	Multipotent adipose-derived stem cells differentiation<br>DMEM-F12-Ham's (low glucose)<br>10 mM HEPES<br>2 mM L-glutamine<br>10 µg/ml transferrin<br>50 IU/mL penicillin<br>50 µg/mL streptomycin<br>**D1-D2**<br>0.5 mM IBMX<br>1 µM dexa<br>860 nM insulin<br>0.1 µM Rosi<br>0.2 nM T3<br>**D2-D12**<br>DMEM/Ham's F12 (low glucose)	Primary white SC preadipocytes differentiation<br>**D1-D5**<br>PBM2<br>IBMX<br>1 µM dexa<br>860 µM insulin<br>Indomethacin<br>**D5-D12**<br>Insulin<br>Indomethacin<br>Concentration of induction reagents NR	mRNA (UCP1)<br><br>Protein (UCP1)	Adipocytes differentiated from multipotent adipose-derived stem cells vs from primary white SC preadipocytes<br>↑ UCP1 mRNA<br><br>Adipocytes differentiated from multipotent adipose-derived stem cells<br>Detectable UCP1 protein

		860 nM insulin<br>0.1 μM Rosi<br>0.2 nM T3			
Lee et al., 2012<br>Japan [121]	MADSCs (infants)<br><br>Growth medium<br>DMEM<br>10% FBS<br>10 mg/mL penicillin / streptomycin<br><br>Confluency at induction: NR	Adipocyte differentiation<br>Growth medium +<br>**D1-D3**<br>0.5 mM IBMX<br>1 μM dexa<br>850 nM insulin<br>1 μM Pio<br>± T3 (0.4, 2, 10, 50, 250 nM)<br>**D3-D10**<br>850 nM insulin<br>1 μM Pio	Cells differentiated in the presence vs absence of T3 (D1-D3)<br><br>Cells differentiated in the presence of different T3 concentrations<br><br>Cells differentiated in the presence of T3 at different time points	mRNA (UCP1, PGC1α, NRF1, CIDEA, ELOVL3, TFAM, PRDM16)<br><br>Protein (UCP1)<br><br>Mitochondrial content (CytC, COX4, COX8a mRNA, CytC protein, MitoTracker®)<br><br>OCR (basal and isoproterenol-stimulated, Seahorse®)	Cells differentiated in the presence vs absence of T3 (250 nM, D1-D3)<br>↑ UCP1, PGC1α, NRF1, CIDEA, ELOVL3 mRNA<br>↔ TFAM, PRDM16 mRNA<br>↑ UCP1 protein<br>↑ Mitochondrial content (CytC, COX4, COX8a mRNA, CytC protein, MitoTracker staining)<br>↑ OCR (basal and 1 μM isoproterenol stimulated)<br><br>Cells differentiated in the presence of different T3 concentrations<br>↑ UCP1 mRNA (10, 50, 250 nM)<br>↑ PGC1α mRNA (250 nM)<br>↑ NRF1 mRNA (250 nM)<br>↔ TFAM mRNA<br><br>Cells differentiated in the presence of T3 at different time points<br>↑ UCP1 mRNA at D1-D3 > D7-D10 = D1-D10<br>↔ UCP1 mRNA D-5/D-2, D-2/D0
Jo et al., 2011<br>South Korea [122]	SVF from WAT (abdominal SC)<br><br>Growth medium<br>DMEM<br>10% FBS<br>1% penicillin/streptomycin 5000 IU/mL<br><br>Confluency at induction: NR	Growth medium +<br>**D1-D14**<br>0.5 mM IBMX<br>1 μM dexa<br>10 μM insulin<br>200 μM indomethacin	Differentiated vs-non-differentiated cells<br><br>Different time points of adipocytic differentiation	mRNA (UCP1)	Differentiated vs-nondifferentiated cells<br>↑ UCP1 mRNA<br><br>Different time points of adipocytic differentiation<br>↑ UCP1 mRNA (D7 > D14)<br>↔ UCP1 mRNA (D1, D4)
Lee et al, 2011<br>Australia [123]	SVF from adipose tissue (supraclavicular, neck SC)<br><br>Growth medium<br>DMEM<br>10% NCS<br>10 mM HEPES<br>850 nM insulin<br>1 nM T3<br>100 μM isoprenaline<br>50 U/mL penicillin<br>50 μg/mL streptomycin<br><br>Induction at 100% confluency	Adipocyte differentiation<br>Growth medium<br>**D1-D7**<br>0.5 mM IBMX<br>1 μM dexa<br>850 nM insulin<br>1 μM Rosi<br>1 nM T3<br>100 μM isoprenaline<br>1 nM GH<br>1 nM IGF-1<br>33 μM biotin<br>17 μM pantothenate<br>10 μg/mL transferrin	Adipocytes differentiated from SVF from supraclavicular vs neck SC adipose tissue	Protein (UCP1)	Adipocytes differentiated from SVF from supraclavicular vs neck SC adipose tissue<br>↑ UCP1 protein

Pisani et al, 2011<br>Austria, France, US [124]	ADSCs (SC WAT)<br><br>Growth medium<br>DMEM<br>10% FCS<br>60 µg/mL penicillin<br>50 µg/mL streptomycin<br>2.5 ng/mL FGF2 (until confluency)<br><br>Induction 2 d after confluency	DMEM-F12-Ham's<br>10 µg/mL transferrin<br>**D1-D3**<br>0.5 mM IBMX<br>1 µM dexa<br>850 nM insulin<br>0.2 nM T3<br>**D3-D17**<br>850 nM insulin<br>0.2 nM T3<br>± 0.1 µM Rosi (D3-D9, D3-D16, D3-D9 and D14-D16)	Cells treated vs non-treated with Rosi	mRNA (UCP1, UCP2, UCP3, CPT1M, CIDEA)	Cells treated vs non-treated with Rosi<br>↑ UCP1, UCP2, CPT1M, CIDEA mRNA<br>↔ UCP3 mRNA
Bogacka et al, 2005<br>Poland [125]	SVF from WAT (mixture of abdominal, hip, flank, thigh, breast SC)<br><br>Growth medium<br>DMEM-F10<br>FBS<br>Antibiotics<br><br>Confluency at induction: NR	Adipocyte differentiation<br>DMEM-F10<br>3% FBS<br>Penicillin/streptomycin/ amphotericin B<br>33 µM biotin<br>17 µM pantothenate<br>**D1-D3**<br>0.2 mM IBMX<br>1 µM dexa<br>100 nM insulin<br>1 µM Rosi<br>**D3-10**<br>1 µM dexa<br>100 nM insulin<br>± 1 µM Rosi through differentiation<br>± 4 µM FSK for 96 h before harvesting	Rosi treated vs non-treated cells<br><br>FSK treated vs non-treated cells	mRNA (UCP1, PGC1α, TFAM, CYTC)<br><br>Mitochondrial content (mtDNA, ND1 mRNA)	Rosi treated vs non-treated cells<br>↑ UCP1, CYTc mRNA<br>↔ PGC1α, TFAM, PPARα mRNA<br>↑ mitochondrial content<br><br>FSK treated vs non-treated cells<br>↑ PGC1α, TFAM, CYTc, UCP1, PPARα mRNA<br>↑ mitochondrial content
Tiraby et al., 2003<br>France [126]	SVF from WAT (abdominal SC)<br><br>Growth medium<br>DMEM-F12<br>10% FCS<br><br>Confluency at induction: NR	Adipocyte differentiation<br>DMEM-F12-Ham's<br>15 mM $NaHCO_3$<br>15 mM HEPES<br>33 µM biotin<br>17 µM pantotenate<br>10 µg/mL transferrin<br>50 µg/mL gentamicin<br>**D1-D3**<br>66 nM insulin<br>1 nM T3<br>1 µg/mL troglitazone<br>100 nM cortisol<br>**D3-D13**<br>Medium only<br>**D13-D15**<br>Medium ± 1 µM Rosi	Cells treated vs non-treated with Rosi	mRNA (UCP1)	Cells treated vs non-treated with Rosi<br>↑ UCP1 mRNA

Digby et al, 1998<br>UK, US [127]	SVF from WAT (abdominal SC, omental, perirenal)<br><br>Growth medium<br>DMEM-F12-Ham's<br>10% FBS<br>Antibiotics<br>2 mM L-glutamine<br><br>Induction at 100% confluency	Adipoctyte differentiation<br>**D1-D20**<br>Growth medium without FBS +<br>33 μM biotin<br>17 μM pantothenate<br>500 nM insulin<br>0.2 nM T3<br>± 0.1 μM Rosi	Cells diferentiated in the presence vs absence of Rosi<br><br>Cells differentiated from the SVF from different adipose depots	mRNA (UCP1)	Cells diferentiated in the presence vs absence of Rosi<br>UCP1 mRNA: more frequent induction in perirenal vs omental and abdominal SC<br><br>Cells differentiated from the SVF from different adipose depots<br>UCP1 mRNA: perirenal > omental and abdominal SC

ADRB3: beta-3 adrenergic receptor; ADRB1: beta-1 adrenergic receptor; ADSCs: Adipose-derived stem cells; BAT: brown adipose tissue; BATLAS: brown adipose tissue atlas/map (web tool); bFGF: basic fibroblast growth factor; BM: bone marrow; BME: Basal Medium Eagle ; BMP4: bone morphogenetic protein 4; BMP7: bone morphogenetic protein 7; cAMP: cyclic adenosine monophosphate; CAR4: carbonic anhydrase 4; CD34: hematopoietic progenitor cell antigen 34; CD40: CD40 molecule; CD137: tumor necrosis factor receptor superfamily member 9; C/EBPβ: CCAAT enhancer binding protein beta; CIDEA: cell death inducing DFFA like effector A; CIDEC: cell death-inducing DFFA-like effector C; COX4: cytochrome c oxidase subunit 4; COX7A: cytochrome c oxidase subunit 7A; COX8B: cytochrome c oxidase subunit 8B; CPT1M: carnitine palmitoyltransferase 1B; CITED1: CREB-binding protein/p300 interacting transactivator with Asp/Glu-rich C-terminal domain protein 1; CKMT1: mitochondrial creatine kinase 1; CKMT2: mitochondrial creatine kinase 2; CRISPR: clustered regularly interspaced short palindromic repeats; CytC: cytochrome C1; CYCS: cytochrome C, somatic; Dexa: dexamethasone; DIO2: type 2 deiodinase; DMEM: Dulbecco's Modified Eagle's Medium; DMEM-F12: Dulbecco's Modified Eagle's Medium/Nutrient F-12; DN: deep neck; EBF2: early B-cell factor transcription factor 2; EBM2: endothelial cell growth basal medium; ECAR: extracellular acidification rate; EGF: epidermal growth factor; EGM2: endothelial cell growth medium 2 ; EGM2-MV: microvascular endothelial cell growth medium; ELOVL: fatty acid elongase; FABP3: fatty acid binding protein 3; FBOX31: F-box protein 3; FBS: fetal bovine serum; FCS: fetal calf serum; FGF: fibroblast growth factor; FGF1: fibroblast growth factor 1; FGF2: fibroblast growth factor 2; FNDC5: fibronectin type III domain-containing protein 5; FSK: forskolin; GH: growth hormone; GPT2: glutamic-pyruvic transaminase 2; HEPES: 4-(2-hydroxyethyl)-1-piperazineethanesulfonic acid; HOXC8: homeobox C8; HOXC9: homeobox C9; IBMX: isomethylbuthylxanthine; IGF-1: insulin-like growth factor 1; IMDM: Iscove's modified Dulbecco's medium ; KCNK3: potassium two pore domain channel subfamily K member 3; LHX8: LIM homeobox 8; MADSCs: multipotent adipose-derived stem cells; MEM: Minimum Essential Medium; miR: microRNA; MSC: mesenchymal stem cells; mtDNA: mitochondrial DNA; mtND1: mitochondrially encoded NADH dehydrogenase 1; MTUS: microtubule associated scaffold protein 1; NaHCO3: sodium bicarbonate; NCS: neonatal calf serum; NE: norepinephrine; NRIP1: nuclear receptor-interacting protein 1; NR: not reported; NRF1: nuclear respiratory factor 1; OCR: oxygen consumption rate; P2RX5: purinergic receptor P2X5; PAT2: tramdorin-1 (SLC36A2); PBM2: pre-adipocyte basal medium (Lonza); PDK4: pyruvate dehydrogenase kinase 4; PGC1α: peroxisome proliferator-activated receptor gamma coactivator 1-alpha; PGC1β: peroxisome proliferator-activated receptor gamma coactivator 1-beta; Pio: pioglitazone; PPARα: peroxisome-proliferator activated receptor alpha; PRDM16: PR domain containing 16; ProFAT: profiling of fat tissue types; RNAseq: RNA sequencing; Rosi: Rosiglitazone; SAMM50: sorting and assembly machinery component 50 homolog; SB: SB431542, TGF beta inhibitor; SC: subcutaneous; SLC25A20: carnitine-acylcarnitine translocase; SLC7A10/ASC-1: Asc-type amino acid transporter 1; SHMT1: serine hydroxymethyltransferase 1; SHOX2: short stature homeobox 2; SVF: stromal vascular fraction; T3: triiodothyronine; TBX1: T-box protein 1; TEM: transmission electron microscopy; TFAM: mitochondrial transcription factor A protein; TMEM26: transmembrane protein 26; TOMM20A: translocase of outer mitochondrial membrane 20; UCP1: uncoupling protein 1; UCP2: uncoupling protein 2; UCP3: uncoupling protein 3; VDAC1: voltage-dependent anion channel 1; WAT: white adipose tissue; ZIC1: Zic family member 1.
All peptides are human (recombinant) unless otherwise stated.

Supplementary Table S4. Brown, beige, and overall thermogenic (overlapping brown/beige) RNA and protein markers assessed in the included studies to characterize the thermogenic adipocyte phenotype.

Marker	Role in thermogenic adipocyte phenotype	References
**Brown adipocytes**		
FBOX31	Brown adipocyte marker (function not defined)	[1]
miR133β	Negative regulation of brown adipocyte fate	[2]
miR206	Precursor fate - brown adipocyte differentiation	[3]
PDK4	Lipolysis, thermogenesis	[4]
ZIC1	Brown adipocyte differentiation and function [1]	[5]
**Beige adipocytes**		
CAR4	Beige adipocyte differentiation and function [1]	[6]
CD137	Beige marker (function not defined)	[6]
CD40	Beige marker (function not defined)	[6]
CITED1	Beige marker (function not defined)	[6]
KCNK3	Negative regulation of thermogenesis	[7]
SAMM50	Mitochondrial function / thermogenesis	[8]
SHOX2	Negative regulation of lipolysis and thermogenesis	[9]
TBX1	Precursor fate - beige adipocyte<br>Beige adipocyte function	[10]
TMEM26	Beige marker (function not defined)	[6]
**Overlapping brown/beige (overall thermogenic adipocyte) markers**		
ADRB1/ADRB3	Thermogenesis	[6]
C/EBPβ	Thermogenesis	[3]
CIDEA	Thermogenesis	[6]
CIDEC	Negative regulation of lipolysis	[11]
CKMT1/CKMT2	Thermogenesis (dispensible)	[12]
COX4	Thermogenesis	[13]
COX7A	Thermogenesis	[14]

COX8B	Thermogenesis	[15]
CPT1M1	Fatty acid metabolism	[16]
CPT2	Fatty acid metabolism	[16]
CYCS	Thermogenesis	[17]
CYTC	Thermogenesis	[18]
DIO2	Thermogenesis	[19]
EBF2	Thermogenic adipocyte differentiation	[20]
ELOVL3/ELOVL6	Fatty acid synthesis	[21]
FABP3	Lipid transport and metabolism	[22]
GPT2	Fatty acid metabolism	[23]
HOXC8	Negative regulation of thermogenic adipocyte differentiation	[6]
HOXC9	Thermogenic adipocyte marker (function not defined)	[6]
LHX8	Thermogenic adipocyte marker (function not defined)	[6]
MTUS1	Negative regulation of thermogenesis	[24]
NRF1	Mitochondrial biogenesis and function	[25]
OXR1	Mitochondrial function, thermogenic adipocyte differentiation	[26]
P2RX5	Thermogenic adipocyte differentiation	[27]
PAT2/SLC36A2	Thermogenesis	[28]
PGC1α/PGC1β	Mitochondrial biogenesis and function	[6]
PM20D1	Lipid and amino acid metabolism	[29]
PPARα	Lipid metabolism	[6]
PRDM16	Thermogenic adipocyte differentiation	[6]
SHMT1	Lipid metabolism and mitochondrial biogenesis	[30]
SCL25A20	Acylcarnitine transport for fatty acid oxidation	[31]
SCL7A10	Amino acid transport, thermogenesis	[32]
TFAM	Negative regulation of thermogenesis	[33]
TOMM20a	Mitochondrial function, thermogenesis	[34]
UCP1/UCP2/UCP3	Thermogenesis	[6]

VDAC1	Mitochondrial function	[35]

[1] Presumed role in thermogenic adipocytes.

ADRB3: beta-3 adrenergic receptor; ADRB1: beta-1 adrenergic receptor; CAR4: carbonic anhydrase 4; CD40: CD40 molecule; CD137: tumor necrosis factor receptor superfamily member 9; C/EBPβ: CCAAT enhancer binding protein beta; CIDEA: cell death inducing DFFA like effector A; CIDEC: cell death-inducing DFFA-like effector C; COX4: cytochrome c oxidase subunit 4; COX7A: cytochrome c oxidase subunit 7A; COX8B: cytochrome c oxidase subunit 8B; CPT1M: carnitine palmitoyltransferase 1B; CITED1: CREB-binding protein/p300 interacting transactivator with Asp/Glu-rich C-terminal domain protein 1; CKMT1: mitochondrial creatine kinase 1; CKMT2: mitochondrial creatine kinase 2; CYTC: cytochrome C1; CYCS: cytochrome C, somatic; DIO2: type 2 deiodinase; EBF2: early B-cell factor transcription factor 2; ELOVL: fatty acid elongase; FABP3: fatty acid binding protein 3; FBOX31: F-box protein 3; GPT2: glutamic-pyruvic transaminase 2; HOXC8: homeobox C8; HOXC9: homeobox C9; KCNK3: potassium two pore domain channel subfamily K member 3; LHX8: LIM homeobox 8; miR: microRNA; MTUS: microtubule associated scaffold protein 1; NRF1: nuclear respiratory factor 1; P2RX5: purinergic receptor P2X5; PAT2: tramdorin-1 (SLC36A2); PDK4: pyruvate dehydrogenase kinase 4; PGC1α: peroxisome proliferator-activated receptor gamma coactivator 1-alpha; PGC1β: peroxisome proliferator-activated receptor gamma coactivator 1-beta; PPARα: peroxisome-proliferator activated receptor alpha; PRDM16: PR domain containing 16; SAMM50: sorting and assembly machinery component 50 homolog; SLC25A20: carnitine-acylcarnitine translocase; SLC7A10/ASC-1: Asc-type amino acid transporter 1; SHMT1: serine hydroxymethyltransferase 1; SHOX2: short stature homeobox 2; TBX1: T-box protein 1; TFAM: mitochondrial transcription factor A protein; TMEM26: transmembrane protein 26; TOMM20A: translocase of outer mitochondrial membrane 20; UCP1: uncoupling protein 1; UCP2: uncoupling protein 2; UCP3: uncoupling protein 3; VDAC1: voltage-dependent anion channel 1; ZIC1: Zic family member 1.

References

1. Jash, S.; Banerjee, S.; Lee, M.J.; Farmer, S.R.; Puri, V. CIDEA Transcriptionally Regulates UCP1 for Britening and Thermogenesis in Human Fat Cells. *iScience* **2019**, 20, 73-89, doi:10.1016/j.isci.2019.09.011.
2. Yin, H.; Pasut, A.; Soleimani, V.D.; Bentzinger, C.F.; Antoun, G.; Thorn, S.; Seale, P.; Fernando, P.; van Ijcken, W.; Grosveld, F.; et al. MicroRNA-133 controls brown adipose determination in skeletal muscle satellite cells by targeting Prdm16. *Cell Metab* **2013**, 17, 210-224, doi:10.1016/j.cmet.2013.01.004.
3. Trajkovski, M.; Ahmed, K.; Esau, C.C.; Stoffel, M. MyomiR-133 regulates brown fat differentiation through Prdm16. *Nat Cell Biol* **2012**, 14, 1330-1335, doi:10.1038/ncb2612.
4. Markussen, L.K.; Rondini, E.A.; Johansen, O.S.; Madsen, J.G.S.; Sustarsic, E.G.; Marcher, A.-B.; Hansen, J.B.; Gerhart-Hines, Z.; Granneman, J.G.; Mandrup, S. Lipolysis regulates major transcriptional programs in brown adipocytes. *Nat Commun* **2022**, 13, 3956, doi:10.1038/s41467-022-31525-8.
5. Waldén, T.B.; Hansen, I.R.; Timmons, J.A.; Cannon, B.; Nedergaard, J. Recruited vs. nonrecruited molecular signatures of brown, "brite," and white adipose tissues. Am J Physiol Endocrinol Metab **2012**, 302, E19-E31, doi:10.1152/ajpendo.00249.2011.
6. Pilkington, A.C.; Paz, H.A.; Wankhade, U.D. Beige Adipose Tissue Identification and Marker Specificity-Overview. *Front Endocrinol* **2021**, 12, 599134, doi:10.3389/fendo.2021.599134.
7. Chen, Y.; Zeng, X.; Huang, X.; Serag, S.; Woolf, C.J.; Spiegelman, B.M. Crosstalk between KCNK3-Mediated Ion Current and Adrenergic Signaling Regulates Adipose Thermogenesis and Obesity. Cell 2017, 171, 836-848.e813, doi:10.1016/j.cell.2017.09.015.
8. Park, S.J.; Shon, D.H.; Kim, J.H.; Ryu, Y.H.; Ko, Y. SAMM50 Regulates Thermogenesis of Beige Adipocytes Differentiated from Human Adipose-Derived Stem Cells by Balancing Mitochondrial Dynamics. *Int J Mol Sci* **2022**, 23, doi:10.3390/ijms23126764.
9. Lee, K.Y.; Yamamoto, Y.; Boucher, J.; Winnay, J.N.; Gesta, S.; Cobb, J.; Blüher, M.; Kahn, C.R. Shox2 is a molecular determinant of depot-specific adipocyte function. *Proc Natl Acad Sci* USA **2013**, 110, 11409-11414, doi:10.1073/pnas.1310331110.
10. Markan, K.R.; Boland, L.K.; King-McAlpin, A.Q.; Claflin, K.E.; Leaman, M.P.; Kemerling, M.K.; Stonewall, M.M.; Amendt, B.A.; Ankrum, J.A.; Potthoff, M.J. Adipose TBX1 regulates β-adrenergic sensitivity in subcutaneous adipose tissue and thermogenic capacity in vivo. *Mol Metab* **2020**, 36, 100965, doi:10.1016/j.molmet.2020.02.008.
11. Fu, Q.; Wang, P.; Li, W.; Cai, Z.; Zhao, S.; Ling, W.; Li, M.; Tang, X.; Song, Z. Partial inhibition of adipose CIDEC improves insulin sensitivity and increases energy expenditure in high-fat diet-fed mice via activating ATGL-PPARα pathway. *BBA - Molecular and Cell Biology of Lipids* **2025**, 1870, 159659, doi:https://doi.org/10.1016/j.bbalip.2025.159659.

12. Politis-Barber, V.; Petrick, H.L.; Raajendiran, A.; DesOrmeaux, G.J.; Brunetta, H.S.; Dos Reis, L.M.; Mori, M.A.; Wright, D.C.; Watt, M.J.; Holloway, G.P. Ckmt1 is Dispensable for Mitochondrial Bioenergetics Within White/Beige Adipose Tissue. *Function* **2022**, 3, zqac037, doi:10.1093/function/zqac037.
13. Lee, J.Y.; Takahashi, N.; Yasubuchi, M.; Kim, Y.I.; Hashizaki, H.; Kim, M.J.; Sakamoto, T.; Goto, T.; Kawada, T. Triiodothyronine induces UCP-1 expression and mitochondrial biogenesis in human adipocytes. *Am J Physiol Cell Physiol* **2012**, 302, C463-472, doi:10.1152/ajpcell.00010.2011.
14. Maurer, S.F.; Fromme, T.; Grossman, L.I.; Hüttemann, M.; Klingenspor, M. The brown and brite adipocyte marker Cox7a1 is not required for non-shivering thermogenesis in mice. *Sci Rep* **2015**, 5, 17704, doi:10.1038/srep17704.
15. Paulo, E.; Wu, D.; Wang, Y.; Zhang, Y.; Wu, Y.; Swaney, D.L.; Soucheray, M.; Jimenez-Morales, D.; Chawla, A.; Krogan, N.J.; Wang, B. Sympathetic inputs regulate adaptive thermogenesis in brown adipose tissue through cAMP-Salt inducible kinase axis. *Sci Rep* **2018**, 8, 11001, doi:10.1038/s41598-018-29333-6.
16. Lee, J.; Ellis, J.M.; Wolfgang, M.J. Adipose fatty acid oxidation is required for thermogenesis and potentiates oxidative stress-induced inflammation. *Cell Rep* **2015**, 10, 266-279, doi:10.1016/j.celrep.2014.12.023.
17. Whitehead, A.; Krause, F.N.; Moran, A.; MacCannell, A.D.V.; Scragg, J.L.; McNally, B.D.; Boateng, E.; Murfitt, S.A.; Virtue, S.; Wright, J.; et al. Brown and beige adipose tissue regulate systemic metabolism through a metabolite interorgan signaling axis. *Nat Commun* **2021**, 12, 1905, doi:10.1038/s41467-021-22272-3.
18. Hüttemann, M.; Frank, V.; Kadenbach, B. The possible role of isoforms of cytochrome c oxidase subunit VIa in mammalian thermogenesis. *Cell Mol Life Sci* **1999**, 55, 1482-1490, doi:10.1007/s000180050387.
19. de Jesus, L.A.; Carvalho, S.D.; Ribeiro, M.O.; Schneider, M.; Kim, S.W.; Harney, J.W.; Larsen, P.R.; Bianco, A.C. The type 2 iodothyronine deiodinase is essential for adaptive thermogenesis in brown adipose tissue. *J Clin Invest* **2001**, 108, 1379-1385, doi:10.1172/jci13803.
20. Angueira, A.R.; Shapira, S.N.; Ishibashi, J.; Sampat, S.; Sostre-Colón, J.; Emmett, M.J.; Titchenell, P.M.; Lazar, M.A.; Lim, H.W.; Seale, P. Early B Cell Factor Activity Controls Developmental and Adaptive Thermogenic Gene Programming in Adipocytes. Cell Rep 2020, 30, 2869-2878.e2864, doi:10.1016/j.celrep.2020.02.023.
21. Tan, C.Y.; Virtue, S.; Bidault, G.; Dale, M.; Hagen, R.; Griffin, J.L.; Vidal-Puig, A. Brown Adipose Tissue Thermogenic Capacity Is Regulated by Elovl6. *Cell Rep* **2015**, 13, 2039-2047, doi:10.1016/j.celrep.2015.11.004.
22. Vergnes, L.; Chin, R.; Young, S.G.; Reue, K. Heart-type Fatty Acid-binding Protein Is Essential for Efficient Brown Adipose Tissue Fatty Acid Oxidation and Cold Tolerance. *J Biol Chem* **2011**, 286, 380-390, doi:https://doi.org/10.1074/jbc.M110.184754.
23. Vámos, A.; Arianti, R.; Vinnai, B.; Alrifai, R.; Shaw, A.; Póliska, S.; Guba, A.; Csősz, É.; Csomós, I.; Mocsár, G.; et al. Human abdominal subcutaneous-derived active beige adipocytes carrying FTO rs1421085 obesity-risk alleles exert lower thermogenic capacity. *Front Cell Dev Biol* **2023**, 11, 1155673, doi:10.3389/fcell.2023.1155673.
24. Zhao, X.Y.; Zhao, B.C.; Li, H.L.; Liu, Y.; Wang, B.; Li, A.Q.; Zeng, T.S.; Hui, H.X.; Sun, J.; Cikes, D.; et al. MTCH2 Suppresses Thermogenesis by Regulating Autophagy in Adipose Tissue. *Adv Sci (Weinh)* **2025**, 12, e2416598, doi:10.1002/advs.202416598.
25. Bartelt, A.; Widenmaier, S.B.; Schlein, C.; Johann, K.; Goncalves, R.L.S.; Eguchi, K.; Fischer, A.W.; Parlakgül, G.; Snyder, N.A.; Nguyen, T.B.; et al. Brown adipose tissue thermogenic adaptation requires Nrf1-mediated proteasomal activity. *Nat Med* **2018**, 24, 292-303, doi:10.1038/nm.4481.
26. Sellayah, D.; Sikder, D. Orexin receptor-1 mediates brown fat developmental differentiation. *Adipocyte* **2012**, 1, 58-63, doi:10.4161/adip.18965.
27. Razzoli, M.; McGonigle, S.; Sahu, B.S.; Rodriguez, P.; Svedberg, D.; Rao, L.; Ruocco, C.; Nisoli, E.; Vezzani, B.; Frontini, A.; Bartolomucci, A. A key role for P2RX5 in brown adipocyte differentiation and energy homeostasis. *Adipocyte 2024*, 13, 2421745, doi:10.1080/21623945.2024.2421745.
28. Shu, H.; Zhang, J.; Cheng, D.; Zhao, X.; Ma, Y.; Zhang, C.; Zhang, Y.; Jia, Z.; Liu, Z. The Role of Proton-Coupled Amino Acid Transporter 2 (SLC36A2) in Cold-Induced Thermogenesis of Mice. *Nutrients* **2023**, 15, doi:10.3390/nu15163552.
29. Long, J.Z.; Svensson, K.J.; Bateman, L.A.; Lin, H.; Kamenecka, T.; Lokurkar, I.A.; Lou, J.; Rao, R.R.; Chang, M.R.; Jedrychowski, M.P.; et al. The Secreted Enzyme PM20D1 Regulates Lipidated Amino Acid Uncouplers of Mitochondria. *Cell* **2016**, 166, 424-435, doi:10.1016/j.cell.2016.05.071.

30. Majeed, Y.; Halabi, N.; Madani, A.Y.; Engelke, R.; Bhagwat, A.M.; Abdesselem, H.; Agha, M.V.; Vakayil, M.; Courjaret, R.; Goswami, N.; et al. SIRT1 promotes lipid metabolism and mitochondrial biogenesis in adipocytes and coordinates adipogenesis by targeting key enzymatic pathways. *Sci Rep* **2021**, 11, 8177, doi:10.1038/s41598-021-87759-x.
31. Cohen, P.; Kajimura, S. The cellular and functional complexity of thermogenic fat. *Nat Rev Mol Cell Biol* **2021**, 22, 393-409, doi:10.1038/s41580-021-00350-0.
32. Arianti, R.; Vinnai, B.Á.; Tóth, B.B.; Shaw, A.; Csősz, É.; Vámos, A.; Győry, F.; Fischer-Posovszky, P.; Wabitsch, M.; Kristóf, E.; Fésüs, L. ASC-1 transporter-dependent amino acid uptake is required for the efficient thermogenic response of human adipocytes to adrenergic stimulation. *FEBS Letters* **2021**, 595, 2085-2098, doi:https://doi.org/10.1002/1873-3468.14155.
33. Vernochet, C.; Mourier, A.; Bezy, O.; Macotela, Y.; Boucher, J.; Rardin, Matthew J.; An, D.; Lee, Kevin Y.; Ilkayeva, Olga R.; Zingaretti, Cristina M.; et al. Adipose-Specific Deletion of TFAM Increases Mitochondrial Oxidation and Protects Mice against Obesity and Insulin Resistance. *Cell Metab* **2012**, 16, 765-776, doi:https://doi.org/10.1016/j.cmet.2012.10.016.
34. Nagy, L.; Rauch, B.; Szerafin, T.; Uray, K.; Tóth, A.; Bai, P. Nicotinamide-riboside shifts the differentiation of human primary white adipocytes to beige adipocytes impacting substrate preference and uncoupling respiration through SIRT1 activation and mitochondria-derived reactive species production. *Front Cell Dev Biol* **2022**, 10, 979330, doi:10.3389/fcell.2022.979330.
35. Elsen, M.; Raschke, S.; Tennagels, N.; Schwahn, U.; Jelenik, T.; Roden, M.; Romacho, T.; Eckel, J. BMP4 and BMP7 induce the white-to-brown transition of primary human adipose stem cells. *Am J Physiol Cell Physiol* **2014**, 306, C431-C440, doi:10.1152/ajpcell.00290.2013.

Supplementary Table S5. Excluded articles and reasons for exclusion (n = 153).

Authors	Reason for exclusion
Kim et al., 2025 [1]	1
Yu et al., 2025 [2]	2
Arianti et al., 2024 [3]	3
Bahn et al., 2024 [4]	4
Bolin et al., 2024 [5]	5
Di Maio et al., 2024 [6]	15
Hayato et al., 2024 [7]	1
Hong et al., 2024 [8]	1
Karanfil et al., 2024 [9]	6
Kim et al., 2024 [10]	1
Ma et al., 2024 [11]	1
Reckziegel et al., 2024 [12]	1
Sakaki et al., 2024 [13]	1
Takeda et al, 2024 [14]	15
Takeda et al., 2024 [15]	3
Wang et al., 2024 [16]	3
Wen et al., 2024 [17]	1
Yadav et al., 2024 [18]	1
Abdul Majeed et al., 2023 [19]	4
Ali et al., 2023 [20]	7
Arianti et al., 2023 [21]	3
Bokhari et al., 2023 [22]	8

Carobbio et al., 2023 [23]	8
Cero et al., 2023 [24]	5
Dong et al., 2023 [25]	1
Escudero et al., 2023 [26]	9
Fu et al., 2023 [27]	1
Michurina et al., 2023 [28]	5
Omran et al., 2023 [29]	3
Pagnotta et al., 2023 [30]	1
Rao et al., 2023 [31]	5
Spinelli et al., 2023 [32]	5
Vaittinen et al., 2023 [33]	8
Acosta et al., 2022 [34]	1
Al Ali et al., 2022 [35]	5
Algieri et al., 2022 [36]	10
Christen et al., 2022 [37]	8
Colitti et al., 2022 [38]	8
Cruciani et al., 2022 [39]	3
Dani et al., 2022 [40]	11
Davies et al., 2022 [41]	1
Ferrando et al., 2022 [42]	5
Gavaldà-Navarro et al., 2022 [43]	4
Guijas et al., 2022 [44]	8
Hong et al., 2022 [45]	1
Jung et al., 2022 [46]	5
Kasza et al., 2022 [47]	2

Monfort-Ferre., 2022 [48]	2
Nahmgoong et al., 2022 [49]	1
Scheele et al., 2022 [50]	8
So et al., 2022 [51]	1
Solivan-Rivera et al., 2022 [52]	16
Takeda et al., 2022 [53]	15
Wang et al., 2022 [54]	1
Yao and Dani., 2022 [55]	9
Zhang et al., 2022 [56]	1
Arianti et al., 2021 [57]	15
Bové et al., 2021 [58]	3
Breining et al., 2021 [59]	5
Carobbio et al., 2021 [60]	5
Chakraborty et al., 2021 [61]	5
Chen et al., 2021 [62]	3
Lao-On et al., 2021 [63]	3
Meng et al., 2021 [64]	2
Senamontree et al., 2021 [65]	5
Shaw et al., 2021 [66]	12
Takeda et al., 2021 [67]lai	15
Van Krieken et al., 2021 [68]	4
Bai et al., 2020 [69]	2
Cattaneo et al., 2020 [70]	1
Colson et al., 2020 [71]	15
Dahlhaus et al., 2020 [72]	8
Ferrari et al., 2020 [73]	1

Frühbeck et al., 2020 [74]	2
Han et al., 2020 [75]	3
Jung et al., 2020 [76]	12
Leménager et al., 2020 [77]	3
Lugo Leija et al., 2020 [78]	1
Nielsen et al., 2020 [79]	5
Oliveira et al., 2020 [80]	3
Overby et al., 2020 [81]	5
Park et al., 2020 [82]	5
Takeda et al., 2020 [83]	15
Vasileva et al., 2020 [84]	8
Zhang et al., 2020 [85]	5
Zlatska et al., 2020 [86]	13
Wu et al., 2020 [87]	2
Chen et al., 2019 [88]	1
Comas et al., 2019 [89]	2
Christian 2019 [90]	11
Fayyad et al., 2019 [91]	5
Harms et al., 2019 [92]	6
Klusóczk et al., 2019 [93]	8
Lee et al., 2019 [94]	14
Pellegrini et al., 2019 [95]	10
Tews et al., 2019 [96]	8
Hafner et al., 2018 [97]	5
Hagberg et al., 2018 [98]	3

Ju et al., 2018 [99]	1
Montanari et al., 2018 [100]	8
Mukherjee et al., 2018 [101]	5
Nakamura et al., 2018 [102]	5
Schmidt et al., 2018 [103]	5
Tang et al., 2018 [104]	5
Zahid et al., 2018 [105]	5
Chechi et al., 2017 [106]	3
Finlin et al., 2017 [107]	3
Guénantin et al., 2017 [108]	5
Hafner et al., 2017 [109]	11
Kriszt et al., 2017 [110]	5
Markussen et al., 2017 [111]	5
Maurizi et al., 2017 [112]	12
Shamsi et al., 2017 [113]	5
Takeda et al., 2017 [114]	3
Tews et al., 2017 [115]	8
Yeo et al., 2017 [116]	3
Zou al., 2017 [117]	3
Adeniran-Catlett., 2016 [118]	12
Gavaldà-Navarro et al., 2016 [119]	2
Ghandour et al., 2016 [120]	15
Giroud et al., 2016 [121]	3
Hafner et al., 2016 [122]	11

Hankir et al., 2016 [123]	3
Huttala et al., 2016 [124]	13
Laiglesia et al., 2016 [125]	3
Pisani et al., 2016 [126]	15
Shukla et al., 2016 [127]	12
Zhang et al., 2016 [128]	15
Docanto et al., 2015 [129]	12
Fernández-Galilea et al., 2015 [130]	3
Guennoun et al., 2015 [131]	5,8
Lin et al., 2015 [132]	12
Moisan et al., 2015 [133]	3
Moreno-Navarrete et al., 2015 [134]	3
Nagano et al., 2015 [135]	2
Shinoda al., 2015 [136]	5
Strong et al., 2015 [137]	2
Yoneshiro et al., 2015 [138]	2
Bugge et al., 2014 [139]	11
De Sousa et al., 2014 [140]	1
Lee et al., 2014 [141]	11
Li et al., 2014 [142]	1
Mohsen -Kanson et al., 2014 [143]	5
Obregon et al., 2014 [144]	2
Aune et al., 2013 [145]	1
Beranger et al., 2013 [146]	2

Ahfeldtet al., 2012 [147]	5
Chung et al., 2012 [148]	11
Elefanty and Stanley et al., 2012 [149]	11
Nishio et al., 2012 [150]	5
Huang et al., 2011 [151]	5
McKay et al., 2006 [152]	2
Zilberfarb et al., 1997 [153]	5

1. Non-human cells (n = 27)
2. No adipocyte differentiation induction (n = 15)
3. Inadequate medium (conditioned) or inadequate medium description (n = 24)
4. Human tissue assessment (n = 4)
5. Immortalized / genetically modified cells (n = 34)
6. Dedifferentiatiated mature adipocytes (n = 2)
7. Inadequate medium (not chemically-induced adipocyte differentiation, n = 1)
8. SGBS cells (n = 11)
9. Organoids (n = 2)
10. Inadequate objective (characterize lipodystrophy adipocytes, n = 2)
11. Reviews, book chapters, commentaries, protocols (n = 11)
12. No assessment of thermogenic markers (n = 7)
13. No thermogenic induction of adipocytes (n = 2)
14. Treatment of mature adipocytes (n = 1)
15. Previously published differentiation conditions (n = 10)
16. Human cells assessed in mice (transplantation, n = 1)

References

1. Kim, S.H.; Park, W.Y.; Kim, B.; Kim, J.H.; Song, G.; Park, J.Y.; Jiao, W.; Jung, S.J.; Ahn, K.S.; Kwak, H.J.; Um, J.Y. FXR-ApoC2 pathway activates UCP1-mediated thermogenesis by

promoting the browning of white adipose tissues. *J Biol Chem* **2025**, *301*, 108181, doi:10.1016/j.jbc.2025.108181.
2. Yu, J.; Gu, X.; Guo, Y.; Gao, M.; Cheng, S.; Meng, M.; Cui, X.; Zhang, Z.; Guo, W.; Yan, D.; et al. E3 ligase FBXW7 suppresses brown fat expansion and browning of white fat. *EMBO Rep* **2025**, *26*, 748-767, doi:10.1038/s44319-024-00337-w.
3. Arianti, R.; Vinnai, B.; Alrifai, R.; Karadsheh, G.; Al-Khafaji, Y.Q.; Póliska, S.; Győry, F.; Fésüs, L.; Kristóf, E. Upregulation of inhibitor of DNA binding 1 and 3 is important for efficient thermogenic response in human adipocytes. *Sci Rep* **2024**, *14*, 28272, doi:10.1038/s41598-024-79634-2.
4. Bahn, Y.J.; Wang, Y.; Dagur, P.; Scott, N.; Cero, C.; Long, K.T.; Nguyen, N.; Cypess, A.M.; Rane, S.G. TGF-β antagonism synergizes with PPARγ agonism to reduce fibrosis and enhance beige adipogenesis. *Mol Metab* **2024**, *90*, 102054, doi:10.1016/j.molmet.2024.102054.
5. Bolin, A.P.; de Fatima Silva, F.; Salgueiro, R.B.; Dos Santos, B.A.; Komino, A.C.M.; Andreotti, S.; de Sousa, É.; de Castro, É.; Real, C.C.; de Paula Faria, D.; et al. Glucocorticoid modulates oxidative and thermogenic function of rat brown adipose tissue and human brown adipocytes. *J Cell Physiol* **2024**, *239*, 1-12, doi:10.1002/jcp.31397.
6. Di Maio, G.; Alessio, N.; Ambrosino, A.; Al Sammarraie, S.H.A.; Monda, M.; Di Bernardo, G. Irisin influences the in vitro differentiation of human mesenchymal stromal cells, promoting a tendency toward beiging adipogenesis. *J Cell Biochem* **2024**, *125*, e30565, doi:10.1002/jcb.30565.
7. Hayato, R.; Matsumoto, T.; Higure, Y. Ca2+ Depletion in the ER Causes Store-Operated Ca2+ Entry via the TRPC6 Channel in Mouse Brown Adipocytes. *Physiol Res* **2024**, *73*, 69-80, doi:10.33549/physiolres.935071.
8. Hong, P.; Wang, D.; Wu, Y.; Zhang, Q.; Liu, P.; Pan, J.; Yu, M.; Tian, W. A novel long noncoding RNA AK029592 contributes to thermogenic adipocyte differentiation. *Stem Cells Transl Med* **2024**, *13*, 985-1000, doi:10.1093/stcltm/szae056.
9. Karanfil, A.S.; Louis, F.; Sowa, Y.; Matsusaki, M. Polyelectrolyte nanofilms on cell surface can induce brown adipogenic differentiation of DFATs. *Biochem Biophys Res Commun* **2024**, *733*, 150432, doi:10.1016/j.bbrc.2024.150432.
10. Kim, S.; Yazawa, T.; Koide, A.; Yoneda, E.; Aoki, R.; Okazaki, T.; Tomita, K.; Watanabe, H.; Muroi, Y.; Testuka, M.; Muranishi, Y. Potential Role of Pig UCP3 in Modulating Adipocyte Browning via the Beta-Adrenergic Receptor Signaling Pathway. *Biology* **2024**, *13*, 284.
11. Yuze, M.; Liu, N.; Shao, X.; Shi, T.; Lin, J.; Liu, B.; Shen, T.; Guo, B.; Jiang, Q. Mechanical loading on osteocytes regulates thermogenesis homeostasis of brown adipose tissue by influencing osteocyte-derived exosomes. *Journal of Orthopaedic Translation* **2024**, *48*, 39-52, doi:https://doi.org/10.1016/j.jot.2024.06.012.
12. Reckziegel, P.; Petrovic, N.; Cannon, B.; Nedergaard, J. Perfluorooctanoate (PFOA) cell-autonomously promotes thermogenic and adipogenic differentiation of brown and white adipocytes. *Ecotoxicol Environ Saf* **2024**, *271*, 115955, doi:10.1016/j.ecoenv.2024.115955.
13. Sakaki, M.; Kamatari, Y.; Kurisaki, A.; Funaba, M.; Hashimoto, O. Activin E upregulates uncoupling protein 1 and fibroblast growth factor 21 in brown adipocytes. *Mol Cell Endocrinol* **2024**, *592*, 112326, doi:10.1016/j.mce.2024.112326.
14. Takeda, Y.; Yoshikawa, T.; Dai, P. Angiotensin II participates in mitochondrial thermogenic functions via the activation of glycolysis in chemically induced human brown adipocytes. *Scientific Reports* **2024**, *14*, 10789, doi:10.1038/s41598-024-61774-0.
15. Takeda, Y.; Dai, P. Functional roles of pantothenic acid, riboflavin, thiamine, and choline in adipocyte browning in chemically induced human brown adipocytes. *Sci Rep* **2024**, *14*, 18252, doi:10.1038/s41598-024-69364-w.

16. Wang, Q.; Su, Y.; Sun, R.; Xiong, X.; Guo, K.; Wei, M.; Yang, G.; Ru, Y.; Zhang, Z.; Li, J.; et al. MIIP downregulation drives colorectal cancer progression through inducing peri-cancerous adipose tissue browning. *Cell & Bioscience* **2024**, *14*, 12, doi:10.1186/s13578-023-01179-0.
17. Wen, Q.; Xie, X.; Ren, Q.; Pan, R.; Du, Y. BDE-99 stimulates generation of aberrant brown/beige adipocytes. *Environ Pollut* **2024**, *347*, 123761, doi:10.1016/j.envpol.2024.123761.
18. Yadav, M.K.; Ishida, M.; Gogoleva, N.; Liao, C.W.; Salim, F.N.; Kanai, M.; Kuno, A.; Hayashi, T.; Shahri, Z.J.; Kulathunga, K.; et al. MAFB in macrophages regulates cold-induced neuronal density in brown adipose tissue. *Cell Rep* **2024**, *43*, 113978, doi:10.1016/j.celrep.2024.113978.
19. Abdul Majeed, S.; Dunzendorfer, H.; Weiner, J.; Heiker, J.T.; Kiess, W.; Körner, A.; Landgraf, K. COBL, MKX and MYOC Are Potential Regulators of Brown Adipose Tissue Development Associated with Obesity-Related Metabolic Dysfunction in Children. *Int J Mol Sci* **2023**, *24*, doi:10.3390/ijms24043085.
20. Ali, U.; Wabitsch, M.; Tews, D.; Colitti, M. Effects of allicin on human Simpson-Golabi-Behmel syndrome cells in mediating browning phenotype. *Frontiers in endocrinology* **2023**, *14*, 1141303, doi:10.3389/fendo.2023.1141303.
21. Arianti, R.; Vinnai, B.Á.; Győry, F.; Guba, A.; Csősz, É.; Kristóf, E.; Fésüs, L. Availability of abundant thiamine determines efficiency of thermogenic activation in human neck area derived adipocytes. *Journal of Nutritional Biochemistry* **2023**, *119*, doi:10.1016/j.jnutbio.2023.109385.
22. Bokhari, M.H.; Halleskog, C.; Shabalina, I.; Bengtsson, T. Use of Isothermal Microcalorimetry to Measure Cellular Heat Production in Thermogenic Adipocytes. *Methods Mol Biol* **2023**, *2662*, 77-85, doi:10.1007/978-1-0716-3167-6_7.
23. Carobbio, S.; Vidal-Puig, A. Differentiation of Human Pluripotent Stem Cells (hPSCs) into Brown-Like Adipocytes. *Methods Mol Biol* **2023**, *2662*, 1-9, doi:10.1007/978-1-0716-3167-6_1.
24. Cero, C.; Shu, W.; Reese, A.L.; Douglas, D.; Maddox, M.; Singh, A.P.; Ali, S.L.; Zhu, A.R.; Katz, J.M.; Pierce, A.E.; et al. Standardized In Vitro Models of Human Adipose Tissue Reveal Metabolic Flexibility in Brown Adipocyte Thermogenesis. *Endocrinology* **2023**, *164*, doi:10.1210/endocr/bqad161.
25. Dong, M.; An, K.; Mao, L. High levels of uric acid inhibit BAT thermogenic capacity through regulation of AMPK. *Am J Physiol Endocrinol Metab* **2023**, *325*, E376-e389, doi:10.1152/ajpendo.00092.2023.
26. Escudero, M.; Vaysse, L.; Eke, G.; Peyrou, M.; Villarroya, F.; Bonnel, S.; Jeanson, Y.; Boyer, L.; Vieu, C.; Chaput, B.; et al. Scalable Generation of Pre-Vascularized and Functional Human Beige Adipose Organoids. *Advanced Science* **2023**, doi:10.1002/advs.202301499.
27. Fu, T.; Sun, W.; Xue, J.; Zhou, Z.; Wang, W.; Guo, Q.; Chen, X.; Zhou, D.; Xu, Z.; Liu, L.; et al. Proteolytic rewiring of mitochondria by LONP1 directs cell identity switching of adipocytes. *Nature cell biology* **2023**, *25*, 848-864, doi:10.1038/s41556-023-01155-3.
28. Michurina, S.; Stafeev, I.; Boldyreva, M.; Truong, V.A.; Ratner, E.; Menshikov, M.; Hu, Y.C.; Parfyonova, Y. Transplantation of Adipose-Tissue-Engineered Constructs with CRISPR-Mediated UCP1 Activation. *Int J Mol Sci* **2023**, *24*, doi:10.3390/ijms24043844.
29. Omran, F.; Murphy, A.M.; Younis, A.Z.; Kyrou, I.; Vrbikova, J.; Hainer, V.; Sramkova, P.; Fried, M.; Ball, G.; Tripathi, G.; et al. The impact of metabolic endotoxaemia on the browning process in human adipocytes. *BMC Med* **2023**, *21*, 154, doi:10.1186/s12916-023-02857-z.
30. Pagnotta, P.; Gantov, M.; Fletcher, S.; Lombardi, A.; Crosbie, M.L.; Santiso, N.; Ursino, A.; Frascarolli, C.; Amato, A.; Dreszman, R.; et al. Peritumoral adipose tissue promotes lipolysis and white adipocytes browning by paracrine action. *Frontiers in endocrinology* **2023**, *14*, 1144016, doi:10.3389/fendo.2023.1144016.

31. Rao, J.; Djeffal, Y.; Chal, J.; Marchianò, F.; Wang, C.H.; Al Tanoury, Z.; Gapon, S.; Mayeuf-Louchart, A.; Glass, I.; Sefton, E.M.; et al. Reconstructing human brown fat developmental trajectory in vitro. *Dev Cell* **2023**, doi:10.1016/j.devcel.2023.08.001.
32. Spinelli, S.; Cossu, V.; Passalacqua, M.; Hansen, J.B.; Guida, L.; Magnone, M.; Sambuceti, G.; Marini, C.; Sturla, L.; Zocchi, E. The ABA/LANCL1/2 Hormone/Receptor System Controls Adipocyte Browning and Energy Expenditure. *Int J Mol Sci* **2023**, *24*, doi:10.3390/ijms24043489.
33. Vaittinen, M.; Ilha, M.; Herbers, E.; Wagner, A.; Virtanen, K.A.; Pietiläinen, K.H.; Pirinen, E.; Pihlajamäki, J. Liraglutide demonstrates a therapeutic effect on mitochondrial dysfunction in human SGBS adipocytes in vitro. *Diabetes Res Clin Pract* **2023**, *199*, 110635, doi:10.1016/j.diabres.2023.110635.
34. Acosta, F.M.; Stojkova, K.; Zhang, J.; Garcia Huitron, E.I.; Jiang, J.X.; Rathbone, C.R.; Brey, E.M. Engineering Functional Vascularized Beige Adipose Tissue from Microvascular Fragments of Models of Healthy and Type II Diabetes Conditions. *J Tissue Eng* **2022**, *13*, 20417314221109337, doi:10.1177/20417314221109337.
35. Al-Ali, M.M.; Khan, A.A.; Fayyad, A.M.; Abdallah, S.H.; Khattak, M.N.K. Transcriptomic profiling of the telomerase transformed Mesenchymal stromal cells derived adipocytes in response to rosiglitazone. *BMC Genom Data* **2022**, *23*, 17, doi:10.1186/s12863-022-01027-z.
36. Algieri, C.; Bernardini, C.; Trombetti, F.; Schena, E.; Zannoni, A.; Forni, M.; Nesci, S. Cellular Metabolism and Bioenergetic Function in Human Fibroblasts and Preadipocytes of Type 2 Familial Partial Lipodystrophy. *Int J Mol Sci* **2022**, *23*, doi:10.3390/ijms23158659.
37. Christen, L.; Broghammer, H.; Rapöhn, I.; Möhlis, K.; Strehlau, C.; Ribas-Latre, A.; Gebhardt, C.; Roth, L.; Krause, K.; Landgraf, K.; et al. Myoglobin-mediated lipid shuttling increases adrenergic activation of brown and white adipocyte metabolism and is as a marker of thermogenic adipocytes in humans. *Clin Transl Med* **2022**, *12*, e1108, doi:10.1002/ctm2.1108.
38. Colitti, M.; Ali, U.; Wabitsch, M.; Tews, D. Transcriptomic analysis of Simpson Golabi Behmel syndrome cells during differentiation exhibit BAT-like function. *TISSUE & CELL* **2022**, *77*, doi:10.1016/j.tice.2022.101822.
39. Cruciani, S.; Garroni, G.; Pala, R.; Coradduzza, D.; Cossu, M.L.; Ginesu, G.C.; Capobianco, G.; Dessole, S.; Ventura, C.; Maioli, M. Metformin and vitamin D modulate adipose-derived stem cell differentiation towards the beige phenotype. *Adipocyte* **2022**, *11*, 356-365, doi:10.1080/21623945.2022.2085417.
40. Dani, V.; Yao, X.; Bruni-Favier, S.; Dani, C. Embryonic origins of the three types of adipocytes and novel in vitro models for studying the development of human adipocytes. *Medecine des Maladies Metaboliques* **2022**, *16*, 689-695, doi:10.1016/j.mmm.2022.09.009.
41. Davies, M.R.; Garcia, S.; Liu, M.; Chi, H.; Kim, H.T.; Raffai, R.L.; Liu, X.; Feeley, B.T. Muscle-Derived Beige Adipose Precursors Secrete Promyogenic Exosomes That Treat Rotator Cuff Muscle Degeneration in Mice and Are Identified in Humans by Single-Cell RNA Sequencing. *Am J Sports Med* **2022**, *50*, 2247-2257, doi:10.1177/03635465221095568.
42. Ferrando, M.; Bruna, F.A.; Romeo, L.R.; Contador, D.; Moya-Morales, D.L.; Santiano, F.; Zyla, L.; Gomez, S.; Lopez-Fontana, C.M.; Calvo, J.C.; et al. Renal peritumoral adipose tissue undergoes a browning process and stimulates the expression of epithelial-mesenchymal transition markers in human renal cells. *Scientific reports* **2022**, *12*, 8687, doi:10.1038/s41598-022-12746-9.
43. Gavaldà-Navarro, A.; Mirra, S.; Manso, Y.; Sánchez-Infantes, D.; Giralt, M.; Soriano, E.; Villarroya, F. The armadillo-repeat containing X-linked protein 3, ARMCX3, is a negative regulator of the browning of adipose tissue associated with obesity. *Int J Obes (Lond)* **2022**, *46*, 1652-1661, doi:10.1038/s41366-022-01169-1.

44. Guijas, C.; To, A.; Montenegro-Burke, J.R.; Domingo-Almenara, X.; Alipio-Gloria, Z.; Kok, B.P.; Saez, E.; Alvarez, N.H.; Johnson, K.A.; Siuzdak, G. Drug-Initiated Activity Metabolomics Identifies Myristoylglycine as a Potent Endogenous Metabolite for Human Brown Fat Differentiation. *METABOLITES* **2022**, *12*, doi:10.3390/metabo12080749.
45. Hong, P.; Wu, Y.; Zhang, Q.; Liu, P.; Zhang, S.; Yu, M.; Tian, W. Identification of thermogenesis-related lncRNAs in small extracellular vesicles derived from adipose tissue. *BMC Genomics* **2022**, *23*, 660, doi:10.1186/s12864-022-08883-0.
46. Jung, I.; Tu-Sekine, B.; Jin, S.; Anokye-Danso, F.; Ahima, R.S.; Brown, T.T.; Kim, S.F. Dolutegravir Suppresses Thermogenesis via Disrupting Uncoupling Protein 1 Expression and Mitochondrial Function in Brown/Beige Adipocytes in Preclinical Models. *J Infect Dis* **2022**, *226*, 1626-1636, doi:10.1093/infdis/jiac175.
47. Kasza, I.; Kühn, J.P.; Völzke, H.; Hernando, D.; Xu, Y.G.; Siebert, J.W.; Gibson, A.L.F.; Yen, C.E.; Nelson, D.W.; MacDougald, O.A.; et al. Contrasting recruitment of skin-associated adipose depots during cold challenge of mouse and human. *J Physiol* **2022**, *600*, 847-868, doi:10.1113/JP280922.
48. Monfort-Ferré, D.; Caro, A.; Menacho, M.; Martí, M.; Espina, B.; Boronat-Toscano, A.; Nuñez-Roa, C.; Seco, J.; Bautista, M.; Espín, E.; et al. The Gut Microbiota Metabolite Succinate Promotes Adipose Tissue Browning in Crohn's Disease. *J Crohns Colitis* **2022**, *16*, 1571-1583, doi:10.1093/ecco-jcc/jjac069.
49. Nahmgoong, H.; Jeon, Y.G.; Park, E.S.; Choi, Y.H.; Han, S.M.; Park, J.; Ji, Y.; Sohn, J.H.; Han, J.S.; Kim, Y.Y.; et al. Distinct properties of adipose stem cell subpopulations determine fat depot-specific characteristics. *Cell metabolism* **2022**, *34*, 458-472.e456, doi:10.1016/j.cmet.2021.11.014.
50. Scheele, C.; Henriksen, T.I.; Nielsen, S. Isolation and Characterization of Human Brown Adipocytes. In *Methods in Molecular Biology*; 2022; Volume 2448, pp. 217-234.
51. So, J.; Taleb, S.; Wann, J.; Strobel, O.; Kim, K.; Roh, H.C. Chronic cAMP activation induces adipocyte browning through discordant biphasic remodeling of transcriptome and chromatin accessibility. *Molecular metabolism* **2022**, *66*, 101619, doi:10.1016/j.molmet.2022.101619.
52. Solivan-Rivera, J.; Yang Loureiro, Z.; DeSouza, T.; Desai, A.; Pallat, S.; Yang, Q.; Rojas-Rodriguez, R.; Ziegler, R.; Skritakis, P.; Joyce, S.; et al. A neurogenic signature involving monoamine Oxidase-A controls human thermogenic adipose tissue development. *Elife* **2022**, *11*, doi:10.7554/eLife.78945.
53. Takeda, Y.; Dai, P. Capsaicin directly promotes adipocyte browning in the chemical compound-induced brown adipocytes converted from human dermal fibroblasts. *Scientific reports* **2022**, *12*, 6612, doi:10.1038/s41598-022-10644-8.
54. Wang, Q.; Li, H.; Tajima, K.; Verkerke, A.R.P.; Taxin, Z.H.; Hou, Z.; Cole, J.B.; Li, F.; Wong, J.; Abe, I.; et al. Post-translational control of beige fat biogenesis by PRDM16 stabilization. *Nature* **2022**, *609*, 151-158, doi:10.1038/s41586-022-05067-4.
55. Yao, X.; Dani, C. A Simple Method for Generating, Clearing, and Imaging Pre-vascularized 3D Adipospheres Derived from Human iPS Cells. *Methods Mol Biol* **2022**, *2454*, 495-507, doi:10.1007/7651_2021_360.
56. Zhang, P.; Wu, W.; Du, C.; Ji, X.; Wang, Y.; Han, Q.; Xu, H.; Li, C.; Xu, Y. RNA-seq profiling of white and brown adipocyte differentiation treated with epigallocatechin gallate. *Sci Data* **2022**, *9*, 41, doi:10.1038/s41597-022-01149-0.
57. Arianti, R.; Vinnai, B.Á.; Tóth, B.B.; Shaw, A.; Csősz, É.; Vámos, A.; Győry, F.; Fischer-Posovszky, P.; Wabitsch, M.; Kristóf, E.; Fésüs, L. ASC-1 transporter-dependent amino acid uptake is required for the efficient thermogenic response of human adipocytes to adrenergic stimulation. *FEBS Letters* **2021**, *595*, 2085-2098, doi:10.1002/1873-3468.14155.

58. Bové, M.; Monto, F.; Guillem-Llobat, P.; Ivorra, M.D.; Noguera, M.A.; Zambrano, A.; Sirerol-Piquer, M.S.; Requena, A.C.; García-Alonso, M.; Tejerina, T.; et al. NT3/TrkC Pathway Modulates the Expression of UCP-1 and Adipocyte Size in Human and Rodent Adipose Tissue. *Frontiers in endocrinology* **2021**, *12*, 630097, doi:10.3389/fendo.2021.630097.
59. Breining, P.; Pedersen, S.B.; Kjolby, M.; Hansen, J.B.; Jessen, N.; Richelsen, B. Parathyroid hormone receptor stimulation induces human adipocyte lipolysis and browning. *European Journal of Endocrinology* **2021**, *184*, 687-697, doi:10.1530/EJE-20-0713.
60. Carobbio, S.; Guenantin, A.C.; Bahri, M.; Rodriguez-Fdez, S.; Honig, F.; Kamzolas, I.; Samuelson, I.; Long, K.; Awad, S.; Lukovic, D.; et al. Unraveling the Developmental Roadmap toward Human Brown Adipose Tissue. *Stem Cell Reports* **2021**, *16*, 641-655, doi:10.1016/j.stemcr.2021.01.013.
61. Chakraborty, S.; Ong, W.K.; Yau, W.W.Y.; Zhou, Z.; Bhanu Prakash, K.N.; Toh, S.A.; Han, W.; Yen, P.M.; Sugii, S. CD10 marks non-canonical PPARγ-independent adipocyte maturation and browning potential of adipose-derived stem cells. *Stem cell research & therapy* **2021**, *12*, 109, doi:10.1186/s13287-021-02179-y.
62. Chen, X.; He, X.; Guo, Y.; Liu, L.; Li, H.; Tan, J.; Feng, W.; Guan, H.; Cao, X.; Xiao, H.; Li, Y. Glucose-dependent insulinotropic polypeptide modifies adipose plasticity and promotes beige adipogenesis of human omental adipose-derived stem cells. *FASEB J* **2021**, *35*, e21534, doi:10.1096/fj.201903253R.
63. Lao-On, U.; Cliff, T.S.; Dalton, S.; Jitrapakdee, S. Pyruvate carboxylase supports basal ATP-linked respiration in human pluripotent stem cell-derived brown adipocytes. *Biochemical and biophysical research communications* **2021**, *569*, 139-146, doi:10.1016/j.bbrc.2021.06.096.
64. Meng, W.; Xiao, T.; Liang, X.; Wen, J.; Peng, X.; Wang, J.; Zou, Y.; Liu, J.; Bialowas, C.; Luo, H.; et al. The miR-182-5p/FGF21/acetylcholine axis mediates the crosstalk between adipocytes and macrophages to promote beige fat thermogenesis. *JCI Insight* **2021**, *6*, doi:10.1172/jci.insight.150249.
65. Senamontree, S.; Lakthan, T.; Charoenpanich, P.; Chanchao, C.; Charoenpanich, A. Betulinic acid decreases lipid accumulation in adipogenesis-induced human mesenchymal stem cells with upregulation of pgc-1a and ucp-1 and post-transcriptional downregulation of adiponectin and leptin secretion. *PeerJ* **2021**, *9*, doi:10.7717/peerj.12321.
66. Shaw, A.; Tóth, B.B.; Király, R.; Arianti, R.; Csomós, I.; Póliska, S.; Vámos, A.; Korponay-Szabó, I.R.; Bacso, Z.; Győry, F.; et al. Irisin Stimulates the Release of CXCL1 From Differentiating Human Subcutaneous and Deep-Neck Derived Adipocytes via Upregulation of NFκB Pathway. *Front Cell Dev Biol* **2021**, *9*, 737872, doi:10.3389/fcell.2021.737872.
67. Takeda, Y.; Yoshikawa, T.; Dai, P. Transcriptome analysis reveals brown adipogenic reprogramming in chemical compound-induced brown adipocytes converted from human dermal fibroblasts. *Scientific reports* **2021**, *11*, 5061, doi:10.1038/s41598-021-84611-0.
68. van Krieken, P.P.; Odermatt, T.S.; Borsigova, M.; Blüher, M.; Wueest, S.; Konrad, D. Oncostatin M suppresses browning of white adipocytes via gp130-STAT3 signaling. *Molecular metabolism* **2021**, *54*, 101341, doi:10.1016/j.molmet.2021.101341.
69. Bai, N.; Ma, J.; Alimujiang, M.; Xu, J.; Hu, F.; Xu, Y.; Leng, Q.; Chen, S.; Li, X.; Han, J.; et al. Bola3 Regulates Beige Adipocyte Thermogenesis via Maintaining Mitochondrial Homeostasis and Lipolysis. *Frontiers in endocrinology* **2020**, *11*, 592154, doi:10.3389/fendo.2020.592154.
70. Cattaneo, P.; Mukherjee, D.; Spinozzi, S.; Zhang, L.; Larcher, V.; Stallcup, W.B.; Kataoka, H.; Chen, J.; Dimmeler, S.; Evans, S.M.; Guimarães-Camboa, N. Parallel Lineage-Tracing Studies Establish Fibroblasts as the Prevailing In Vivo Adipocyte Progenitor. *Cell reports* **2020**, *30*, 571-582.e572, doi:10.1016/j.celrep.2019.12.046.

71. Colson, C.; Batrow, P.L.; Gautier, N.; Rochet, N.; Ailhaud, G.; Peiretti, F.; Amri, E.Z. The Rosmarinus Bioactive Compound Carnosic Acid Is a Novel PPAR Antagonist That Inhibits the Browning of White Adipocytes. *Cells* **2020**, *9*, doi:10.3390/cells9112433.
72. Dahlhaus, M.; Roos, J.; Engel, D.; Tews, D.; Halbgebauer, D.; Funcke, J.B.; Kiener, S.; Schuler, P.J.; Döscher, J.; Hoffmann, T.K.; et al. CD90 Is Dispensable for White and Beige/Brown Adipocyte Differentiation. *Int J Mol Sci* **2020**, *21*, doi:10.3390/ijms21217907.
73. Ferrari, A.; Longo, R.; Peri, C.; Coppi, L.; Caruso, D.; Mai, A.; Mitro, N.; De Fabiani, E.; Crestani, M. Inhibition of class I HDACs imprints adipogenesis toward oxidative and brown-like phenotype. *Biochim Biophys Acta Mol Cell Biol Lipids* **2020**, *1865*, 158594, doi:10.1016/j.bbalip.2019.158594.
74. Frühbeck, G.; Fernández-Quintana, B.; Paniagua, M.; Hernández-Pardos, A.W.; Valentí, V.; Moncada, R.; Catalán, V.; Becerril, S.; Gómez-Ambrosi, J.; Portincasa, P.; et al. FNDC4, a novel adipokine that reduces lipogenesis and promotes fat browning in human visceral adipocytes. *Metabolism* **2020**, *108*, 154261, doi:10.1016/j.metabol.2020.154261.
75. Han, K.H.; Arlian, B.M.; Lin, C.W.; Jin, H.Y.; Kang, G.H.; Lee, S.; Lee, P.C.; Lerner, R.A. Agonist Antibody Converts Stem Cells into Migrating Brown Adipocyte-Like Cells in Heart. *Cells* **2020**, *9*, doi:10.3390/cells9010256.
76. Jung, Y.J.; Kim, H.K.; Cho, Y.; Choi, J.S.; Woo, C.H.; Lee, K.S.; Sul, J.H.; Lee, C.M.; Han, J.; Park, J.H.; et al. Cell reprogramming using extracellular vesicles from differentiating stem cells into white/beige adipocytes. *Sci Adv* **2020**, *6*, eaay6721, doi:10.1126/sciadv.aay6721.
77. Leménager, H.; Fiévet, L.M.A.; Guilloton, F.; Naji, A.; Descamps, J.G.; Chaput, B.; Suganuma, N.; Pagès, J.C.; Sensebé, L.; Carrière, A.; et al. Cell immaturity and white/beige adipocyte potential of primary human adipose-derived stromal cells are restrained by culture-medium TGFβ1. *Stem Cells* **2020**, *38*, 782-796, doi:10.1002/stem.3164.
78. Lugo Leija, H.A.; Velickovic, K.; Bloor, I.; Sacks, H.; Symonds, M.E.; Sottile, V. Cold-induced beigeing of stem cell-derived adipocytes is not fully reversible after return to normothermia. *J Cell Mol Med* **2020**, *24*, 11434-11444, doi:10.1111/jcmm.15749.
79. Nilsen, M.S.; Jersin, R.; Ulvik, A.; Madsen, A.; McCann, A.; Svensson, P.A.; Svensson, M.K.; Nedrebø, B.G.; Gudbrandsen, O.A.; Tell, G.S.; et al. 3-Hydroxyisobutyrate, A Strong Marker of Insulin Resistance in Type 2 Diabetes and Obesity That Modulates White and Brown Adipocyte Metabolism. *Diabetes* **2020**, *69*, 1903-1916, doi:10.2337/db19-1174.
80. de Oliveira, M.; Mathias, L.S.; Rodrigues, B.M.; Mariani, B.G.; Graceli, J.B.; De Sibio, M.T.; Castro Olimpio, R.M.; Fontes Moretto, F.C.; Deprá, I.C.; Nogueira, C.R. The roles of triiodothyronine and irisin in improving the lipid profile and directing the browning of human adipose subcutaneous cells. *Mol Cell Endocrinol* **2020**, *506*, 110744, doi:10.1016/j.mce.2020.110744.
81. Overby, H.; Yang, Y.; Xu, X.; Wang, S.; Zhao, L. Indomethacin promotes browning and brown adipogenesis in both murine and human fat cells. *Pharmacol Res Perspect* **2020**, *8*, e00592, doi:10.1002/prp2.592.
82. Park, M.J.; Liao, J.; Kim, D.I. TC-E 5003, a protein methyltransferase 1 inhibitor, activates the PKA-dependent thermogenic pathway in primary murine and human subcutaneous adipocytes. *FEBS letters* **2020**, *594*, 2923-2930, doi:10.1002/1873-3468.13900.
83. Takeda, Y.; Dai, P. A developed serum-free medium and an optimized chemical cocktail for direct conversion of human dermal fibroblasts into brown adipocytes. *Scientific reports* **2020**, *10*, 3775, doi:10.1038/s41598-020-60769-x.
84. Vasileva, L.V.; Savova, M.S.; Amirova, K.M.; Balcheva-Sivenova, Z.; Ferrante, C.; Orlando, G.; Wabitsch, M.; Georgiev, M.I. Caffeic and Chlorogenic Acids Synergistically Activate Browning Program in Human Adipocytes: Implications of AMPK- and PPAR-Mediated Pathways. *Int J Mol Sci* **2020**, *21*, doi:10.3390/ijms21249740.

85. Zhang, L.; Avery, J.; Yin, A.; Singh, A.M.; Cliff, T.S.; Yin, H.; Dalton, S. Generation of Functional Brown Adipocytes from Human Pluripotent Stem Cells via Progression through a Paraxial Mesoderm State. *Cell Stem Cell* **2020**, *27*, 784-797.e711, doi:10.1016/j.stem.2020.07.013.
86. Zlatska, A.V.; Vasyliev, R.G.; Gordiienko, I.M.; Rodnichenko, A.E.; Morozova, M.A.; Vulf, M.A.; Zubov, D.O.; Novikova, S.N.; Litvinova, L.S.; Grebennikova, T.V.; et al. *Effect of the deuterium on efficiency and type of adipogenic differentiation of human adipose-derived stem cells in vitro*, 1; England, 2020.
87. Wu, C.; Zhang, H.; Lin, X.; Zeng, Y.; Zhang, Y.; Ma, X.; Xue, Y.; Guan, M. Role of PDK4 in insulin signaling pathway in periadrenal adipose tissue of pheochromocytoma patients. *Endocr Relat Cancer* **2020**, *27*, 583-589, doi:10.1530/ERC-20-0293.
88. Chen, Y.; Ikeda, K.; Yoneshiro, T.; Scaramozza, A.; Tajima, K.; Wang, Q.; Kim, K.; Shinoda, K.; Sponton, C.H.; Brown, Z.; et al. Thermal stress induces glycolytic beige fat formation via a myogenic state. *Nature* **2019**, *565*, 180-+, doi:10.1038/s41586-018-0801-z.
89. Comas, F.; Martínez, C.; Sabater, M.; Ortega, F.; Latorre, J.; Díaz-Sáez, F.; Aragonés, J.; Camps, M.; Gumà, A.; Ricart, W.; et al. Neuregulin 4 Is a Novel Marker of Beige Adipocyte Precursor Cells in Human Adipose Tissue. *Front Physiol* **2019**, *10*, 39, doi:10.3389/fphys.2019.00039.
90. Christian, M. In vitro models for study of brown adipocyte biology. In *Handbook of Experimental Pharmacology*; 2019; Volume 251, pp. 85-96.
91. Fayyad, A.M.; Khan, A.A.; Abdallah, S.H.; Alomran, S.S.; Bajou, K.; Khattak, M.N.K. Rosiglitazone Enhances Browning Adipocytes in Association with MAPK and PI3-K Pathways During the Differentiation of Telomerase-Transformed Mesenchymal Stromal Cells into Adipocytes. *Int J Mol Sci* **2019**, *20*, doi:10.3390/ijms20071618.
92. Harms, M.J.; Li, Q.; Lee, S.; Zhang, C.; Kull, B.; Hallen, S.; Thorell, A.; Alexandersson, I.; Hagberg, C.E.; Peng, X.R.; et al. Mature Human White Adipocytes Cultured under Membranes Maintain Identity, Function, and Can Transdifferentiate into Brown-like Adipocytes. *Cell reports* **2019**, *27*, 213-225.e215, doi:10.1016/j.celrep.2019.03.026.
93. Klusóczki, Á.; Veréb, Z.; Vámos, A.; Fischer-Posovszky, P.; Wabitsch, M.; Bacso, Z.; Fésüs, L.; Kristóf, E. Differentiating SGBS adipocytes respond to PPARγ stimulation, irisin and BMP7 by functional browning and beige characteristics. *Scientific reports* **2019**, *9*, 5823, doi:10.1038/s41598-019-42256-0.
94. Lee, M.J.; Jash, S.; Jones, J.E.C.; Puri, V.; Fried, S.K. Rosiglitazone remodels the lipid droplet and britens human visceral and subcutaneous adipocytes ex vivo. *Journal of lipid research* **2019**, *60*, 856-868, doi:10.1194/jlr.M091173.
95. Pellegrini, C.; Columbaro, M.; Schena, E.; Prencipe, S.; Andrenacci, D.; Iozzo, P.; Angela Guzzardi, M.; Capanni, C.; Mattioli, E.; Loi, M.; et al. Altered adipocyte differentiation and unbalanced autophagy in type 2 Familial Partial Lipodystrophy: an in vitro and in vivo study of adipose tissue browning. *Exp Mol Med* **2019**, *51*, 1-17, doi:10.1038/s12276-019-0289-0.
96. Tews, D.; Pula, T.; Funcke, J.B.; Jastroch, M.; Keuper, M.; Debatin, K.M.; Wabitsch, M.; Fischer-Posovszky, P. Elevated UCP1 levels are sufficient to improve glucose uptake in human white adipocytes. *Redox Biol* **2019**, *26*, 101286, doi:10.1016/j.redox.2019.101286.
97. Hafner, A.L.; Mohsen-Kanson, T.; Dani, C. Differentiation of brown adipocyte progenitors derived from human induced pluripotent stem cells. In *Methods in Molecular Biology*; 2018; Volume 1773, pp. 31-39.
98. Hagberg, C.E.; Li, Q.; Kutschke, M.; Bhowmick, D.; Kiss, E.; Shabalina, I.G.; Harms, M.J.; Shilkova, O.; Kozina, V.; Nedergaard, J.; et al. Flow Cytometry of Mouse and Human Adipocytes for the Analysis of Browning and Cellular Heterogeneity. *Cell reports* **2018**, *24*, 2746-2756.e2745, doi:10.1016/j.celrep.2018.08.006.

99. Ju, L.; Chen, S.; Alimujiang, M.; Bai, N.; Yan, H.; Fang, Q.; Han, J.; Ma, X.; Yang, Y.; Jia, W. A novel role for Bcl2l13 in promoting beige adipocyte biogenesis. *Biochemical and biophysical research communications* **2018**, *506*, 485-491, doi:10.1016/j.bbrc.2018.10.034.
100. Montanari, T.; Colitti, M. Simpson-Golabi-Behmel syndrome human adipocytes reveal a changing phenotype throughout differentiation. *Histochem Cell Biol* **2018**, *149*, 593-605, doi:10.1007/s00418-018-1663-z.
101. Mukherjee, S.; Zhang, T.; Lacko, L.A.; Tan, L.; Xiang, J.Z.; Butler, J.M.; Chen, S. Derivation and characterization of a UCP1 reporter human ES cell line. *Stem Cell Res* **2018**, *30*, 12-21, doi:10.1016/j.scr.2018.04.007.
102. Nakamura, K.; Kishida, T.; Ejima, A.; Tateyama, R.; Morishita, S.; Ono, T.; Murakoshi, M.; Sugiyama, K.; Nishino, H.; Mazda, O. Bovine lactoferrin promotes energy expenditure via the cAMP-PKA signaling pathway in human reprogrammed brown adipocytes. *Biometals* **2018**, *31*, 415-424, doi:10.1007/s10534-018-0103-9.
103. Schmidt, E.; Dhaouadi, I.; Gaziano, I.; Oliverio, M.; Klemm, P.; Awazawa, M.; Mitterer, G.; Fernandez-Rebollo, E.; Pradas-Juni, M.; Wagner, W.; et al. LincRNA H19 protects from dietary obesity by constraining expression of monoallelic genes in brown fat. *Nat Commun* **2018**, *9*, 3622, doi:10.1038/s41467-018-05933-8.
104. Tang, Y.; He, Y.; Li, C.; Mu, W.; Zou, Y.; Liu, C.; Qian, S.; Zhang, F.; Pan, J.; Wang, Y.; et al. RPS3A positively regulates the mitochondrial function of human periaortic adipose tissue and is associated with coronary artery diseases. *Cell Discovery* **2018**, *4*, doi:10.1038/s41421-018-0041-2.
105. Zahid, H.; Subbaramaiah, K.; Iyengar, N.M.; Zhou, X.K.; Chen, I.C.; Bhardwaj, P.; Gucalp, A.; Morrow, M.; Hudis, C.A.; Dannenberg, A.J.; Brown, K.A. Leptin regulation of the p53-HIF1α/PKM2-aromatase axis in breast adipose stromal cells: a novel mechanism for the obesity-breast cancer link. *Int J Obes (Lond)* **2018**, *42*, 711-720, doi:10.1038/ijo.2017.273.
106. Chechi, K.; Voisine, P.; Mathieu, P.; Laplante, M.; Bonnet, S.; Picard, F.; Joubert, P.; Richard, D. Functional characterization of the Ucp1-associated oxidative phenotype of human epicardial adipose tissue. *Scientific reports* **2017**, *7*, 15566, doi:10.1038/s41598-017-15501-7.
107. Finlin, B.S.; Zhu, B.B.; Confides, A.L.; Westgate, P.M.; Harfmann, B.D.; Dupont-Versteegden, E.E.; Kern, P.A. Mast Cells Promote Seasonal White Adipose Beiging in Humans. *Diabetes* **2017**, *66*, 1237-1246, doi:10.2337/db16-1057.
108. Guénantin, A.C.; Briand, N.; Capel, E.; Dumont, F.; Morichon, R.; Provost, C.; Stillitano, F.; Jeziorowska, D.; Siffroi, J.P.; Hajjar, R.J.; et al. Functional human beige adipocytes from induced pluripotent stem cells. *Diabetes* **2017**, *66*, 1470-1478, doi:10.2337/db16-1107.
109. Hafner, A.L.; Dani, C. Adipocyte progenitors from human pluripotent stem cells. In *Adipose Tissue Biology: Second Edition*; 2017; pp. 61-68.
110. Kriszt, R.; Arai, S.; Itoh, H.; Lee, M.H.; Goralczyk, A.G.; Ang, X.M.; Cypess, A.M.; White, A.P.; Shamsi, F.; Xue, R.; et al. Optical visualisation of thermogenesis in stimulated single-cell brown adipocytes. *Scientific Reports* **2017**, *7*, doi:10.1038/s41598-017-00291-9.
111. Markussen, L.K.; Isidor, M.S.; Breining, P.; Andersen, E.S.; Rasmussen, N.E.; Petersen, L.I.; Pedersen, S.B.; Richelsen, B.; Hansen, J.B. Characterization of immortalized human brown and white pre-adipocyte cell models from a single donor. *PLoS One* **2017**, *12*, doi:10.1371/journal.pone.0185624.
112. Maurizi, G.; Poloni, A.; Mattiucci, D.; Santi, S.; Maurizi, A.; Izzi, V.; Giuliani, A.; Mancini, S.; Zingaretti, M.C.; Perugini, J.; et al. Human White Adipocytes Convert Into "Rainbow" Adipocytes In Vitro. *J Cell Physiol* **2017**, *232*, 2887-2899, doi:10.1002/jcp.25743.
113. Shamsi, F.; Tseng, Y.H. Protocols for Generation of Immortalized Human Brown and White Preadipocyte Cell Lines. *Methods Mol Biol* **2017**, *1566*, 77-85, doi:10.1007/978-1-4939-6820-6_8.

114. Takeda, Y.; Harada, Y.; Yoshikawa, T.; Dai, P. Direct conversion of human fibroblasts to brown adipocytes by small chemical compounds. *Scientific Reports* **2017**, *7*, doi:10.1038/s41598-017-04665-x.
115. Tews, D.; Fromme, T.; Keuper, M.; Hofmann, S.M.; Debatin, K.M.; Klingenspor, M.; Wabitsch, M.; Fischer-Posovszky, P. Teneurin-2 (TENM2) deficiency induces UCP1 expression in differentiating human fat cells. *Mol Cell Endocrinol* **2017**, *443*, 106-113, doi:10.1016/j.mce.2017.01.015.
116. Yeo, C.R.; Agrawal, M.; Hoon, S.; Shabbir, A.; Shrivastava, M.K.; Huang, S.; Khoo, C.M.; Chhay, V.; Yassin, M.S.; Tai, E.S.; et al. SGBS cells as a model of human adipocyte browning: A comprehensive comparative study with primary human white subcutaneous adipocytes. *SCIENTIFIC REPORTS* **2017**, *7*, doi:10.1038/s41598-017-04369-2.
117. Zou, Y.; Lu, P.; Shi, J.; Liu, W.; Yang, M.; Zhao, S.; Chen, N.; Chen, M.; Sun, Y.; Gao, A.; et al. IRX3 Promotes the Browning of White Adipocytes and Its Rare Variants are Associated with Human Obesity Risk. *EBioMedicine* **2017**, *24*, 64-75, doi:10.1016/j.ebiom.2017.09.010.
118. Adeniran-Catlett, A.E.; Weinstock, L.D.; Bozal, F.K.; Beguin, E.; Caraballo, A.T.; Murthy, S.K. Accelerated adipogenic differentiation of hMSCs in a microfluidic shear stimulation platform. *Biotechnol Prog* **2016**, *32*, 440-446, doi:10.1002/btpr.2211.
119. Gavaldà-Navarro, A.; Moreno-Navarrete, J.M.; Quesada-López, T.; Cairó, M.; Giralt, M.; Fernández-Real, J.M.; Villarroya, F. Lipopolysaccharide-binding protein is a negative regulator of adipose tissue browning in mice and humans. *Diabetologia* **2016**, *59*, 2208-2218, doi:10.1007/s00125-016-4028-y.
120. Ghandour, R.A.; Giroud, M.; Vegiopoulos, A.; Herzig, S.; Ailhaud, G.; Amri, E.Z.; Pisani, D.F. IP-receptor and PPARs trigger the conversion of human white to brite adipocyte induced by carbaprostacyclin. *Biochim Biophys Acta* **2016**, *1861*, 285-293, doi:10.1016/j.bbalip.2016.01.007.
121. Giroud, M.; Pisani, D.F.; Karbiener, M.; Barquissau, V.; Ghandour, R.A.; Tews, D.; Fischer-Posovszky, P.; Chambard, J.C.; Knippschild, U.; Niemi, T.; et al. miR-125b affects mitochondrial biogenesis and impairs brite adipocyte formation and function. *Molecular metabolism* **2016**, *5*, 615-625, doi:10.1016/j.molmet.2016.06.005.
122. Hafner, A.L.; Contet, J.; Ravaud, C.; Yao, X.; Villageois, P.; Suknuntha, K.; Annab, K.; Peraldi, P.; Binetruy, B.; Slukvin, I.I.; et al. Brown-like adipose progenitors derived from human induced pluripotent stem cells: Identification of critical pathways governing their adipogenic capacity. *Scientific Reports* **2016**, *6*, doi:10.1038/srep32490.
123. Hankir, M.K.; Kranz, M.; Gnad, T.; Weiner, J.; Wagner, S.; Deuther-Conrad, W.; Bronisch, F.; Steinhoff, K.; Luthardt, J.; Klöting, N.; et al. A novel thermoregulatory role for PDE10A in mouse and human adipocytes. *EMBO Mol Med* **2016**, *8*, 796-812, doi:10.15252/emmm.201506085.
124. Huttala, O.; Mysore, R.; Sarkanen, J.R.; Heinonen, T.; Olkkonen, V.M.; Ylikomi, T. Differentiation of human adipose stromal cells in vitro into insulin-sensitive adipocytes. *Cell Tissue Res* **2016**, *366*, 63-74, doi:10.1007/s00441-016-2409-7.
125. Laiglesia, L.M.; Lorente-Cebrián, S.; Prieto-Hontoria, P.L.; Fernández-Galilea, M.; Ribeiro, S.M.; Sáinz, N.; Martínez, J.A.; Moreno-Aliaga, M.J. *Eicosapentaenoic acid promotes mitochondrial biogenesis and beige-like features in subcutaneous adipocytes from overweight subjects*, United States, 2016.
126. Pisani, D.F.; Dumortier, O.; Beranger, G.E.; Casamento, V.; Ghandour, R.A.; Giroud, M.; Gautier, N.; Balaguer, T.; Chambard, J.C.; Virtanen, K.A.; et al. Visfatin expression analysis in association with recruitment and activation of human and rodent brown and brite adipocytes. *Adipocyte* **2016**, *5*, 186-195, doi:10.1080/21623945.2015.1122854.

127. Shukla, A.; Slater, J.H.; Culver, J.C.; Dickinson, M.E.; West, J.L. Biomimetic Surface Patterning Promotes Mesenchymal Stem Cell Differentiation. *ACS Appl Mater Interfaces* **2016**, *8*, 21883-21892, doi:10.1021/acsami.5b08978.
128. Zhang, Y.; Xie, C.; Wang, H.; Foss, R.M.; Clare, M.; George, E.V.; Li, S.; Katz, A.; Cheng, H.; Ding, Y.; et al. Irisin exerts dual effects on browning and adipogenesis of human white adipocytes. *Am J Physiol Endocrinol Metab* **2016**, *311*, E530-541, doi:10.1152/ajpendo.00094.2016.
129. Docanto, M.M.; Ham, S.; Corbould, A.; Brown, K.A. Obesity-Associated Inflammatory Cytokines and Prostaglandin E2 Stimulate Glucose Transporter mRNA Expression and Glucose Uptake in Primary Human Adipose Stromal Cells. *J Interferon Cytokine Res* **2015**, *35*, 600-605, doi:10.1089/jir.2014.0194.
130. Fernández-Galilea, M.; Pérez-Matute, P.; Prieto-Hontoria, P.L.; Houssier, M.; Burrell, M.A.; Langin, D.; Martínez, J.A.; Moreno-Aliaga, M.J. α-Lipoic acid treatment increases mitochondrial biogenesis and promotes beige adipose features in subcutaneous adipocytes from overweight/obese subjects. *Biochimica et Biophysica Acta - Molecular and Cell Biology of Lipids* **2015**, *1851*, 273-281, doi:10.1016/j.bbalip.2014.12.013.
131. Guennoun, A.; Kazantzis, M.; Thomas, R.; Wabitsch, M.; Tews, D.; Seetharama Sastry, K.; Abdelkarim, M.; Zilberfarb, V.; Strosberg, A.D.; Chouchane, L. Comprehensive molecular characterization of human adipocytes reveals a transient brown phenotype. *J Transl Med* **2015**, *13*, 135, doi:10.1186/s12967-015-0480-6.
132. Lin, W.C.; Shih, P.H.; Wang, W.; Wu, C.H.; Hsia, S.M.; Wang, H.J.; Hwang, P.A.; Wang, C.Y.; Chen, S.H.; Kuo, Y.T. Inhibitory effects of high stability fucoxanthin on palmitic acid-induced lipid accumulation in human adipose-derived stem cells through modulation of long non-coding RNA. *Food Funct* **2015**, *6*, 2215-2223, doi:10.1039/c5fo00301f.
133. Moisan, A.; Lee, Y.K.; Zhang, J.D.; Hudak, C.S.; Meyer, C.A.; Prummer, M.; Zoffmann, S.; Truong, H.H.; Ebeling, M.; Kiialainen, A.; et al. White-to-brown metabolic conversion of human adipocytes by JAK inhibition. *Nature cell biology* **2015**, *17*, 57-67, doi:10.1038/ncb3075.
134. Moreno-Navarrete, J.M.; Ortega, F.; Moreno, M.; Xifra, G.; Ricart, W.; Fernández-Real, J.M. PRDM16 sustains white fat gene expression profile in human adipocytes in direct relation with insulin action. *Mol Cell Endocrinol* **2015**, *405*, 84-93, doi:10.1016/j.mce.2015.01.042.
135. Nagano, G.; Ohno, H.; Oki, K.; Kobuke, K.; Shiwa, T.; Yoneda, M.; Kohno, N. Activation of classical brown adipocytes in the adult human perirenal depot is highly correlated with PRDM16-EHMT1 complex expression. *PLoS One* **2015**, *10*, e0122584, doi:10.1371/journal.pone.0122584.
136. Shinoda, K.; Luijten, I.H.; Hasegawa, Y.; Hong, H.; Sonne, S.B.; Kim, M.; Xue, R.; Chondronikola, M.; Cypess, A.M.; Tseng, Y.H.; et al. Genetic and functional characterization of clonally derived adult human brown adipocytes. *Nature medicine* **2015**, *21*, 389-394, doi:10.1038/nm.3819.
137. Strong, A.L.; Ohlstein, J.F.; Biagas, B.A.; Rhodes, L.V.; Pei, D.T.; Tucker, H.A.; Llamas, C.; Bowles, A.C.; Dutreil, M.F.; Zhang, S.; et al. Leptin produced by obese adipose stromal/stem cells enhances proliferation and metastasis of estrogen receptor positive breast cancers. *Breast Cancer Res* **2015**, *17*, 112, doi:10.1186/s13058-015-0622-z.
138. Yoneshiro, T.; Saito, M. Activation and recruitment of brown adipose tissue as anti-obesity regimens in humans. *Ann Med* **2015**, *47*, 133-141, doi:10.3109/07853890.2014.911595.
139. Bugge, A.; Dib, L.; Collins, S. Measuring Respiratory Activity of Adipocytes and Adipose Tissues in Real Time. In *METHODS OF ADIPOSE TISSUE BIOLOGY, PT B*; 2014; Volume 538, pp. 233-247.

140. De Sousa, M.; Porras, D.P.; Perry, C.G.; Seale, P.; Scimè, A. p107 is a crucial regulator for determining the adipocyte lineage fate choices of stem cells. *Stem Cells* **2014**, *32*, 1323-1336, doi:10.1002/stem.1637.
141. Lee, Y.K.; Cowan, C.A. Differentiation of white and brown adipocytes from human pluripotent stem cells. *Methods Enzymol* **2014**, *538*, 35-47, doi:10.1016/B978-0-12-800280-3.00003-7.
142. Li, Y.; Fromme, T.; Schweizer, S.; Schöttl, T.; Klingenspor, M. Taking control over intracellular fatty acid levels is essential for the analysis of thermogenic function in cultured primary brown and brite/beige adipocytes. *EMBO Reports* **2014**, *15*, 1069-1076, doi:10.15252/embr.201438775.
143. Mohsen-Kanson, T.; Hafner, A.L.; Wdziekonski, B.; Takashima, Y.; Villageois, P.; Carrière, A.; Svensson, M.; Bagnis, C.; Chignon-Sicard, B.; Svensson, P.A.; et al. Differentiation of human induced pluripotent stem cells into brown and white adipocytes: role of Pax3. *Stem Cells* **2014**, *32*, 1459-1467, doi:10.1002/stem.1607.
144. Obregon, M.J. Changing white into brite adipocytes. Focus on "BMP4 and BMP7 induce the white-to-brown transition of primary human adipose stem cells". *Am J Physiol Cell Physiol* **2014**, *306*, C425-427, doi:10.1152/ajpcell.00015.2014.
145. Aune, U.L.; Ruiz, L.; Kajimura, S. Isolation and differentiation of stromal vascular cells to beige/brite cells. *J Vis Exp* **2013**, doi:10.3791/50191.
146. Beranger, G.E.; Karbiener, M.; Barquissau, V.; Pisani, D.F.; Scheideler, M.; Langin, D.; Amri, E.Z. In vitro brown and "brite"/"beige" adipogenesis: human cellular models and molecular aspects. *Biochim Biophys Acta* **2013**, *1831*, 905-914, doi:10.1016/j.bbalip.2012.11.001.
147. Ahfeldt, T.; Schinzel, R.T.; Lee, Y.K.; Hendrickson, D.; Kaplan, A.; Lum, D.H.; Camahort, R.; Xia, F.; Shay, J.; Rhee, E.P.; et al. Programming human pluripotent stem cells into white and brown adipocytes. *Nature cell biology* **2012**, *14*, 209-219, doi:10.1038/ncb2411.
148. Chung, S.; Okla, M.; Ha, J.H.; Lee, M.A. Brown adipocyte commitment of primary human adipose stem cells in vitro. *FASEB JOURNAL* **2012**, *26*.
149. Elefanty, A.G.; Stanley, E.G. Efficient generation of adipocytes in a dish. *Nature Cell Biology* **2012**, *14*, 126-127, doi:10.1038/ncb2430.
150. Nishio, M.; Yoneshiro, T.; Nakahara, M.; Suzuki, S.; Saeki, K.; Hasegawa, M.; Kawai, Y.; Akutsu, H.; Umezawa, A.; Yasuda, K.; et al. Production of functional classical brown adipocytes from human pluripotent stem cells using specific hemopoietin cocktail without gene transfer. *Cell metabolism* **2012**, *16*, 394-406, doi:10.1016/j.cmet.2012.08.001.
151. Huang, P.I.; Chen, Y.C.; Chen, L.H.; Juan, C.C.; Ku, H.H.; Wang, S.T.; Chiou, S.H.; Chiou, G.Y.; Chi, C.W.; Hsu, C.C.; et al. *PGC-1α mediates differentiation of mesenchymal stem cells to brown adipose cells*, 11; Japan, 2011.
152. Mackay, D.L.; Tesar, P.J.; Liang, L.N.; Haynesworth, S.E. Characterizing medullary and human mesenchymal stem cell-derived adipocytes. *J Cell Physiol* **2006**, *207*, 722-728, doi:10.1002/jcp.20617.
153. Zilberfarb, V.; Piétri-Rouxel, F.; Jockers, R.; Krief, S.; Delouis, C.; Issad, T.; Strosberg, A.D. Human immortalized brown adipocytes express functional β3-adrenoceptor coupled to lipolysis. *Journal of Cell Science* **1997**, *110*, 801-807.

Supplementary Table S6. Risk of bias assessed in the included "*in vitro*" studies (n=XX) by NTP/OHAT Risk of Bias Rating Tool for Human and Animal Studies. Risk of bias was categorized as (++) when definitely low risk of bias; (+) probably Low risk of bias; (-) Probably High risk of bias, (--) definitely high risk of bias, and "NA" not applicable.

Author	Q1	Q2	Q3	Q4	Q5	Q6	Q7	Q8	Q9	Q10	Score / Risk
Batrow et al., 2025	NA	NA	NA	NA	++	+	+	++	++	++	Low
Giroud et al., 2025	NA	NA	NA	NA	NA	NA	+	++	++	++	Low
Desai et al., 2024	NA	NA	NA	NA	++	+	+	++	++	++	Low
Díez-Sainz et al., 2024	NA	NA	NA	NA	++	+	+	++	++	++	Low
Wu et al., 2024	NA	NA	NA	NA	NA	NA	+	++	++	++	Low
Colson et al., 2023	NA	NA	NA	NA	++	+	+	++	++	++	Low
Di Maio et al., 2023	NA	NA	NA	NA	++	+	+	++	++	++	Low
Palani et al., 2023	NA	NA	NA	NA	++	+	+	++	++	++	Low
Shon et al., 2023	NA	NA	NA	NA	++	+	+	++	++	++	Low
Suchacki et al., 2023	NA	NA	NA	NA	++	+	+	++	++	++	Low
Vámos et al., 2023	NA	NA	NA	NA	++	+	+	++	++	++	Low
Vinnai et al., 2023	NA	NA	NA	NA	++	+	+	++	++	++	Low
Wu et al., 2023	NA	NA	NA	NA	NA	NA	+	++	++	++	Low
Ayissi et al., 2022	NA	NA	NA	NA	++	+	++	++	++	++	Low

Farrar et al., 2022	NA	NA	NA	NA	+	+	+	+	++	+	Low
Fu et al., 2022	NA	NA	NA	NA	++	+	++	++	++	++	Low
Guillemet et al., 2022	NA	NA	NA	NA	++	+	++	++	++	++	Low
He et al., 2022	NA	NA	NA	NA	++	+	+	++	++	++	Low
Herbers et al, 2022	NA	NA	NA	NA	++	+	+	++	++	++	Low
Lin et al., 2022	NA	NA	NA	NA	++	+	+	++	++	++	Low
Nagy et al., 2022	NA	NA	NA	NA	++	+	+	++	++	++	Low
Niemann et al., 2022	NA	NA	NA	NA	NA	NA	++	++	++	++	Low
Park et al, 2022	NA	NA	NA	NA	++	+	+	++	++	++	Low
Park et al., 2022	NA	NA	NA	NA	++	+	+	++	++	++	Low
Porras et al, 2022	NA	NA	NA	NA	++	+	+	++	++	++	Low
Takeda et al., 2022	NA	NA	NA	NA	++	+	+	++	++	++	Low
Vámos et al., 2022	NA	NA	NA	NA	++	+	++	++	++	++	Low
Bokhari et al., 2021	NA	NA	NA	NA	++	+	++	++	++	++	Low
Cero et al, 2021	NA	NA	NA	NA	++	+	+	++	++	++	Low
Di Maio et al., 2021	NA	NA	NA	NA	++	+	++	+	++	++	Low
Jiao et al., 2021	NA	NA	NA	NA	+	+	++	++	++	++	Low

Nascimento et al., 2021	NA	NA	NA	NA	++	+	+	+	++	++	Low
Tóth et al., 2021	NA	NA	NA	NA	++	+	++	++	++	++	Low
Tsagkaraki et al., 2021	NA	NA	NA	NA	++	+	++	++	++	++	Low
Xia et al., 2021	NA	NA	NA	NA	++	+	++	++	++	++	Low
Halbgebauer et al., 2020	NA	NA	NA	NA	++	+	+	++	++	++	Low
Kroon et al., 2020	NA	NA	NA	NA	++	+	+	++	++	++	Low
Li et al., 2020	NA	NA	NA	NA	++	+	+	++	++	++	Low
Markan et al., 2020	NA	NA	NA	NA	++	+	+	++	++	++	Low
Michurina et al., 2020	NA	NA	NA	NA	++	+	+	++	++	++	Low
Moon et al., 2020	NA	NA	NA	NA	++	+	+	++	++	++	Low
Nascimento et al., 2020	NA	NA	NA	NA	++	+	+	++	++	++	Low
Qiu et al., 2020	NA	NA	NA	NA	++	+	+	++	++	++	Low
Saha et al., 2020	NA	NA	NA	NA	++	+	+	++	++	++	Low
Singh et al., 2020	NA	NA	NA	NA	++	+	+	++	++	++	Low
Szatmári-Tóth et al., 2020	NA	NA	NA	NA	++	+	+	++	++	++	Low
Tran et al., 2020	NA	NA	NA	NA	++	+	+	++	++	++	Low
Alessio et al, 2019	NA	NA	NA	NA	NA	NA	+	++	++	++	Low

Hedesan et al., 2019	NA	NA	NA	NA	++	+	+	++	++	++	Low
Jash et al., 2019	NA	NA	NA	NA	++	+	+	++	++	++	Low
Jespersen et al., 2019	NA	NA	NA	NA	++	+	+	++	++	++	Low
Kim et al., 2019	NA	NA	NA	NA	++	+	+	++	++	++	Low
Li et al., 2019	NA	NA	NA	NA	++	+	+	++	++	++	Low
Li et al., 2019	NA	NA	NA	NA	++	+	+	++	++	++	Low
Min et al., 2019	NA	NA	NA	NA	++	+	+	++	++	++	Low
Nagy et al., 2019	NA	NA	NA	NA	++	+	+	++	++	++	Low
Velickovic et al., 2019	NA	NA	NA	NA	NA	NA	+	++	++	++	Low
Raajendiran et al., 2019	NA	NA	NA	NA	++	+	+	++	++	++	Low
West et al, 2019	NA	NA	NA	NA	++	+	+	++	++	++	Low
Wu et al., 2019	NA	NA	NA	NA	++	+	+	++	++	++	Low
Berry et al., 2018	NA	NA	NA	NA	++	+	+	++	++	++	Low
Ghandour et al, 2018	NA	NA	NA	NA	++	+	+	++	++	++	Low
Haynes et al., 2018	NA	NA	NA	NA	++	+	+	++	++	++	Low
Khanh et al., 2018	NA	NA	NA	NA	++	+	+	++	++	++	Low
Loh et al., 2018	NA	NA	NA	NA	++	+	+	++	++	++	Low

Nascimento et al., 2018	NA	NA	NA	NA	++	+	+	++	++	++	Low
Pisani et al., 2018	NA	NA	NA	NA	++	+	+	++	++	++	Low
Rashnonejad et al, 2018	NA	NA	NA	NA	++	+	+	++	++	++	Low
Velickovic et al., 2018	NA	NA	NA	NA	++	+	+	++	++	++	Low
Wang et al, 2018	NA	NA	NA	NA	++	+	+	++	++	++	Low
Su et al, 2018	NA	NA	NA	NA	++	+	+	++	++	++	Low
Zhang et al., 2018	NA	NA	NA	NA	++	+	+	++	++	++	Low
Cambria et al, 2017	NA	NA	NA	NA	++	+	+	++	++	++	Low
Jiang et al., 2017	NA	NA	NA	NA	NA	NA	+	++	++	++	Low
Kim et al, 2017	NA	NA	NA	NA	++	+	+	++	++	++	Low
Liczano et al., 2017	NA	NA	NA	NA	++	+	+	++	++	++	Low
Nyman et al., 2017	NA	NA	NA	NA	++	+	+	++	++	++	Low
Pino et al., 2017	NA	NA	NA	NA	++	+	+	++	++	++	Low
Rebello et al., 2017	NA	NA	NA	NA	++	+	+	++	++	++	Low
Yang et al., 2017	NA	NA	NA	NA	++	+	+	++	++	++	Low
Abdul-Rahman et al, 2016	NA	NA	NA	NA	++	+	+	++	++	++	Low
Barbagallo et al, 2016	NA	NA	NA	NA	++	+	+	++	++	++	Low

Barquissau et al, 2016	NA	NA	NA	NA	++	+	+	++	++	++	Low
Fleckenstein-Elsen et al, 2016	NA	NA	NA	NA	++	+	+	++	++	++	Low
Giroud et al., 2016	NA	NA	NA	NA	++	+	+	++	++	++	Low
Keplac et al., 2016	NA	NA	NA	NA	NA	NA	+	++	++	++	Low
Kroistóf et al., 2016	NA	NA	NA	NA	++	+	+	++	++	++	Low
Lee et al, 2016	NA	NA	NA	NA	++	+	+	++	++	++	Low
Min et al, 2016	NA	NA	NA	NA	++	+	+	++	++	++	Low
Okla et al, 2016	NA	NA	NA	NA	++	+	+	++	++	++	Low
Vargas et al, 2016	NA	NA	NA	NA	++	+	+	++	++	++	Low
Wang et al., 2016	NA	NA	NA	NA	++	+	+	++	++	++	Low
Wu et al., 2016	NA	NA	NA	NA	++	+	+	++	++	++	Low
Barclay et al., 2015	NA	NA	NA	NA	++	+	+	++	++	++	Low
Gustafson et al., 2015	NA	NA	NA	NA	++	+	+	++	++	++	Low
Hartig et al, 2015	NA	NA	NA	NA	++	+	+	++	++	++	Low
Kouidhi et al, 2015	NA	NA	NA	NA	++	+	+	++	++	++	Low
Loft et al., 2015	NA	NA	NA	NA	++	+	+	++	++	++	Low
Seiler et al, 2015	NA	NA	NA	NA	++	+	+	++	++	++	Low

van den Beukel et al, 2015	NA	NA	NA	NA	++	+	+	++	++	++	Low
Vargas et al., 2015	NA	NA	NA	NA	NA	NA	+	++	++	++	Low
Xu et al, 2015	NA	NA	NA	NA	++	+	+	++	++	++	Low
Xue et al, 2015	NA	NA	NA	NA	++	+	+	++	++	++	Low
Zhu et al., 2015	NA	NA	NA	NA	++	+	+	++	++	++	Low
Di Franco et al., 2014	NA	NA	NA	NA	++	+	+	++	++	++	Low
Elsen et al, 2014	NA	NA	NA	NA	++	+	+	++	++	++	Low
Karbiener et al., 2014	NA	NA	NA	NA	++	+	+	++	++	++	Low
Kern et al., 2014	NA	NA	NA	NA	++	+	+	++	++	++	Low
Murholm et al., 2013	NA	NA	NA	NA	++	+	+	++	++	++	Low
Silva et al, 2013	NA	NA	NA	NA	++	+	+	++	++	++	Low
Lee et al., 2012	NA	NA	NA	NA	++	+	+	++	++	++	Low
Jo et al., 2011	NA	NA	NA	NA	++	+	+	++	++	++	Low
Lee et al, 2011	NA	NA	NA	NA	++	+	+	++	++	++	Low
Pisani et al, 2011	NA	NA	NA	NA	++	+	+	++	++	++	Low
Bogacka et al, 2005	NA	NA	NA	NA	++	+	+	++	++	++	Low
Tiraby et al., 2003	NA	NA	NA	NA	++	+	+	++	++	++	Low

Digby et al, 1998	NA	NA	NA	NA	++	+	+	++	++	++	Low

1. Was administered dose or exposure level adequately randomized?
2. Was allocation to study groups adequately concealed?
3. Did selection of study participants result in appropriate comparison groups?
4. Did the study design or analysis account for important confounding and modifying variables?
5. Were experimental conditions identical across study groups?
6. Were the research personnel and human subjects blinded to the study group during the study?
7. Were outcome data complete without attrition or exclusion from analysis?
8. Can we be confident in the exposure characterization?
9. Can we be confident in the outcome assessment?
10. Were all measured outcomes reported?
